# Wild bootstrap for counting process-based statistics: a martingale theory-based approach

**DOI:** 10.1007/s10985-025-09659-w

**Published:** 2025-07-28

**Authors:** Marina T. Dietrich, Dennis Dobler, Mathisca C. M. de Gunst

**Affiliations:** 1 Department of Mathematics, Vrije Universiteit Amsterdam, 1081 HV Amsterdam, The Netherlands; 2 Present Address: Department of Mathematics, University Augsburg, 86159 Augsburg, Germany; 3 Present Address: Department of Statistics, TU Dortmund University, 44227 Dortmund, Germany; 4 Present Address: Research Center Trustworthy Data Science and Security, University Alliance Ruhr, Dortmund, Germany

**Keywords:** Counting processes, Martingale theory, Resampling, Statistical inference, Survival analysis, Wild bootstrap

## Abstract

The wild bootstrap is a popular resampling method in the context of time-to-event data analysis. Previous works established the large sample properties of it for applications to different estimators and test statistics. It can be used to justify the accuracy of inference procedures such as hypothesis tests or time-simultaneous confidence bands. This paper provides a general framework for establishing large sample properties in a unified way by using martingale structures. This framework includes most of the well-known parametric, semiparametric and nonparametric statistical methods in time-to-event analysis. Along the way of proving the validity of the wild bootstrap, a new variant of Rebolledo’s martingale central limit theorem for counting process-based martingales is developed as well.

**Supplementary Information:**

The online version contains supplementary material available at 10.1007/s10985-025-09659-w.

## Introduction

In medical studies about, say, the 5-year survival chances of patients following a therapy, not only the point estimate after 5 years is of interest, but also a confidence interval which quantifies the estimation uncertainty. Furthermore, it makes an essential difference for the patient whether the survival chances fall rather swiftly or slowly towards the 5-year survival chance; this has an influence on the expected remaining lifetime. For this reason, it is more instructive to construct *time-simultaneous confidence bands* for the survival curve function. For such, and also for all hypothesis tests, the distribution of the corresponding (test) statistics, which typically involve stochastic processes, are generally unknown and need to be approximated.

A solution to this challenge is the use of resampling techniques, such as random permutation, the bootstrap (Efron 1979), or the wild bootstrap (Chien-Fu 1986). Certain variants of these techniques were also proposed for incomplete, e.g., independent left-truncated or right-censored, time-to-event data. Early references are Efron (1981) and Akritas (1986) for the classical bootstrap (drawing with replacement from the individual data points), Neuhaus (1993) for random permutation (of the censoring indicators), and Lin et al. (1993) for the wild bootstrap.

Because of its popularity, computational efficiency, and flexibility, we focus in the present paper on the wild bootstrap as the method of choice in the context of survival and event history analysis. To mention a few related works, in Lin (1994) and Dobler et al. (2019) the wild bootstrap is applied to Cox models, and in Lin (1997), Beyersmann et al. (2013), and Dobler et al. (2017) to cumulative incidence functions in competing risks models; the latter two works allowed for more general multipliers, compared to the commonly used standard normal ones. Fine and Gray (1999) proposed a multiplier bootstrap in the context of proportional subdistribution hazard models. Spiekerman and Lin (1998) considered multivariate failure time models, Lin et al. (2000) means in semiparametric models, and Scheike and Zhang (2003) Cox-Aalen models, and corresponding resampling options; these three papers focused on more general marginal hazard rate models, rather than intensity models. Bluhmki et al. (2018) and (Bluhmki et al. 2019) analyzed Aalen–Johansen estimators in general Markovian multi-state models and general Nelson–Aalen estimators, respectively. Among the more recent works in survival or event history analysis, the following is an incomplete list of papers which utilize multiplier bootstraps: Hiabu et al. (2021) within excess additive regression models with two survival time scales; Titman and Putter (2022) about test for the Markov property in general multi-state models; Bakoyannis (2021) for confidence bands based on clustered, nonhomogeneous Markovian multi-state processes. In their monograph, Martinussen and Scheike (2006) exemplified various applications of such multiplier bootstraps, also from a practical point of view.

In this paper, we develop a rigorous theory to justify the use of the wild bootstrap under various survival analysis models. The eligible statistic should be linear in counting process-based integrals. Additionally, individual counting processes may have multiple jumps each. The potential models may be nonparametric, semiparametric, or parametric (regression) models as long as the intensity process related to the counting process is modelled. In particular, any multiplicative intensity model is covered in our unified approach. In other words, this paper provides an umbrella theory for a large variety of specific applications of the wild bootstrap in the context of counting processes. As in many of the above-mentioned articles, we employ the wild bootstrap for mimicking the martingale processes related to individual counting processes by replacing the martingale increments with the randomly perturbed counting process increments. The involved multipliers may follow a general, possibly non-normal distribution with zero mean, unit variance, and finite fourth moment. In order to verify the asymptotic validity of the wild bootstrap as an approximation procedure, we show that the asymptotic distribution of the resampled process coincides with that of the statistic of interest. Our proofs rely on weak regularity conditions and, differently from those in the above-mentioned articles, are developed in a novel way based on the martingale theory for counting processes as given in Rebolledo (1980) instead of on the commonly used variant of this theorem presented in Andersen et al. (1993). This more general version of Rebolledos martingale central limit theorem is needed for both the counting process-based statistic, and the wild bootstrap counterpart. In the former case, an underlying martingale assumption does not hold in general. In the latter case, it is needed to also accommodate multipliers that follow a general, possibly asymmetrical distribution. In this way, our approach solves an open problem of handling the Lindeberg condition in a widely applicable manner.

As a rule of thumb, the wild bootstrap considered in this paper is available for resampling survival or event history data if all of the the following criteria are met: (i) the data are i.i.d.; (ii) the intensity process of counting processes is modeled; (iii) the estimators are asymptotically linear and martingale-based. Possible relaxations of these conditions are discussed at the end of this paper.

This paper is organized as follows. In Sect. 2, we introduce the general set-up, the precise form of the counting process-based statistic, and derive its asymptotic distribution. In Sect. 3, we define the wild bootstrap counterpart of the statistic under consideration and study its asymptotic distribution. Furthermore, we illustrate our findings with the Cox model throughout Sects. 2 and 3, and with some additional, well-known examples in Sect. 4. Finally, in Sect. 5, we provide a discussion. All proofs are given in a Supporting Information file available online.

## Notation, model, and convergence of counting process-based estimators

### Basic model and asymptotic representation

Let $N_1(t), \dots , N_n(t)$, $t\in \mathcal {T}$, be independent and identically distributed counting processes. Each individual counting process $N_i$, $i=1, \dots , n$, has in total $n_i = N_i(\tau )$ jumps of size 1 at observable (random) event times $T_{i,1},\ldots ,T_{i,n_i}$, with no two jumps at the same time. Here, $\mathcal {T} = [0,\tau ]$ is a finite interval. Let ${\textbf {N}}=(N_1,\ldots ,N_n)$ denote the *n*-variate counting process. The at-risk indicator for individual *i* is denoted by $Y_i(t)$, $t\in \mathcal {T}$, $i=1, \dots , n$, and the *n*-variate aggregation by ${\textbf {Y}} = (Y_1,\ldots ,Y_n)$. Additionally, an individual *d*-variate, possibly time-dependent covariate vector $\tilde{{\textbf {Z}}}_i(t)$, $t\in \mathcal {T}, i=1, \dots , n$, may also be available. The observable vector of covariates is ${{\textbf {Z}}}_i = \tilde{{\textbf {Z}}}_i Y_i$, $i=1,\ldots ,n$. The collections of all *n* observable *d*-dimensional covariate vectors are denoted by ${{\textbf {Z}}}(t)$, $t\in \mathcal {T}$. The collection of all data is $({{\textbf {N}}}(t),{{\textbf {Y}}}(t),{{\textbf {Z}}} (t), t\in \mathcal {T})$. Let $\varvec{\beta } \in \mathbb {R}^q$, $q \ge d$, be a parametric model component whereof *d* entries specify the influence of ${\textbf {Z}}$ on the jump times of ${\textbf {N}}$ and the remaining $(q-d)$ entries could, for example, be the parameters of the baseline hazard in a parametric regression model. The simplest case of $q=0$ corresponds to a nonparametric model; cf. Examples 1a–1g, 3, and 4 below for some non- and semiparametric settings. Finally, $(\varOmega ,\mathcal {A},\mathbb {P})$ denotes the underlying probability space, and ${\mathop {\longrightarrow }\limits ^{\mathbb {P}}}$, ${\mathop {\longrightarrow }\limits ^{\mathcal {L}}}$ denote convergence in probability and law, respectively. Typically, multivariate quantities are written in bold type and the finiteness of a stochastic quantity is meant almost surely.

We will illustrate the general theory throughout the paper by means of the Cox model.

#### Example 1a

*(Cox model)* The ordinary Cox model is a semiparametric regression model with multiplicative intensity process and at most one event time per individual, that is, $N_i(t) \in \{0,1\}$, $i=1,\ldots , n$. Let $Y_i(t)$ be the at-risk indicator of individual *i*. Given the *d*-variate predictable (with respect to a suitable filtration) and bounded covariate vectors ${{\textbf {Z}}}_i(t)$, $t\in \mathcal {T}$, the intensity process of $N_i$ is

$${\mathbb {E}}(d N_i(t) | Y_i(s), {{\textbf {Z}}}_i(s): s \le t) = \lambda _i(t,{{\textbf {Z}}}_i(t),\varvec{\beta }_0) dt = Y_i(t)\alpha _i(t,{{\textbf {Z}}}_i(t),\varvec{\beta }_0) dt, t\in \mathcal {T},$$

with

$$\alpha _i(t,{{\textbf {Z}}}_i(t),\varvec{\beta }_0) = \alpha _0(t) \exp (\varvec{\beta }_0^\top {{\textbf {Z}}}_i(t) ), \quad t\in \mathcal {T},$$

$i=1,\dots ,n$. Here, $\alpha _0$ is the so-called baseline hazard rate for an individual with the zero covariate vector and $\varvec{\beta }_0$ is the true regression coefficient. In this case, the processes $M_i(t) = N_i(t) - \varLambda _i(t,{{\textbf {Z}}}_i(t),\varvec{\beta }_0)$, $t\in \mathcal {T}$, are martingales with respect to a suitable filtration, where $\varLambda _i(t,{{\textbf {Z}}}_i(t),\varvec{\beta }) = \int _0^t \lambda _i(u,{{\textbf {Z}}}_i(t),\varvec{\beta }) du$. The Breslow estimator for the cumulative baseline hazard function $A_0(t) = \int _0^t \alpha _0(u) du$, $t\in \mathcal {T}$, is given by

$${\hat{A}}_{0,n}(t, \varvec{\hat{\beta}}_n) =\frac{1}{n} \sum _{i=1}^n \int _0^t \frac{nJ(u)}{S^{(0)}_n(u,\varvec{\hat{\beta}}_n)} dN_i(u), \quad t\in \mathcal {T},$$

where $\varvec{\hat{\beta}}_n$ is the solution to the score equation ${{\textbf {U}}}_n(\tau ,\varvec{\beta }) = 0$, with score statistic ${{\textbf {U}}}_n(t,\varvec{\beta }) = \sum _{i=1}^n \int _0^t ({{\textbf {Z}}}_i(u) - {{{\textbf {S}}}^{(1)}_n(u,\varvec{\beta })}/{S^{(0)}_n(u, \varvec{\beta })}) dN_i(u)$. Here, $\tau>0$ is the terminal evaluation time on the treatment time-scale, and

$$S^{(0)}_n(t,\varvec{\beta }) = \sum _{i=1}^n Y_i(t) \exp ({{\textbf {Z}}}_i^\top (t) \varvec{\beta }),$$

$${{\textbf {S}}}^{(m)}_n(t, \varvec{\beta }) = \sum _{i=1}^n Y_i(t) {{\textbf {Z}}}_i(t)^{\otimes m} \exp ({{\textbf {Z}}}_i^\top (t) \varvec{\beta }), \ t\in \mathcal {T},$$

$$m\in \{1,2\}$$, where $${{\textbf {Z}}}_i(t)^{\otimes 1} = {{\textbf {Z}}}_i(t)$$ and $${{\textbf {Z}}}_i(t)^{\otimes 2} = {{\textbf {Z}}}_i(t){{\textbf {Z}}}_i(t)^\top$$.

$$\square$$

In the context of survival analysis, the respective estimator or test statistic, ${\hat{A}}_{0,n}(\cdot , \varvec{\hat{\beta}}_n)$ in Example 1a, is often linear in counting process-based integrals, where the individual integral is formed with respect to some integrand. The form of the integrand depends on the particular estimator or the particular test statistic, while the linear counting process-based structure remains the same. In particular, one is often interested in the estimation of a vector-valued function ${{\textbf {X}}}(t)$, $t\in \mathcal {T}$, of dimension *p* by a counting process-based statistic of the form

1 $$\begin{aligned} {{\textbf {X}}}_n(t) =\frac{1}{n} \sum _{i=1}^n \int _0^t {{\textbf {k}}}_{n, i}(u,\varvec{\hat{\beta}}_n) d N_i(u), \quad t\in \mathcal {T}. \end{aligned}$$

Here, the *p*-dimensional integrands ${{\textbf {k}}}_{n, i}(t, {\varvec{\beta }})$ defined on $\varOmega \times \mathcal {T}\times \mathbb {R}^q$ are stochastic processes that are not necessarily independent, with ${{\textbf {k}}}_{n, i}(\cdot , {\varvec{\beta }})$ uniformly bounded and predictable for ${\varvec{\beta }} = {\varvec{\beta }_0}$, and ${{\textbf {k}}}_{n, i}(t,\cdot )$ almost surely continuously differentiable in $\varvec{\beta }$, $i=1,\ldots ,n$. We assume that $\varvec{\hat{\beta}}_n$ is a consistent estimator of the true model parameter $\varvec{\beta }_0$ with

2 $$\begin{aligned} \varvec{\hat{\beta}}_n - \varvec{\beta }_0 = O_p(n^{-1/2}). \end{aligned}$$

Additionally, we impose an assumption on the asymptotic representation of $\sqrt{n}(\varvec{\hat{\beta}}_n-\varvec{\beta }_0)$ for $n\rightarrow \infty$, which will be specified later in this section.

#### Example 1b

*(Cox model continued)* In the case of the Cox model, we have $X(t) = A_0(t)$ and $X_n(t)= {\hat{A}}_{0,n}(t, \varvec{\hat{\beta}}_n)$ with integrand $k_{n}(t,\varvec{\beta }_0) = {\displaystyle {\frac{n J(t)}{S^{(0)}_n(t,\varvec{\beta }_0)}}}$, $t\in \mathcal {T}$. In particular, $k_{n}(\cdot ,\varvec{\beta }_0)$ as a function in *t* is bounded by *J* on ${\mathcal {T}}$ and predictable due to the predictability of $Y_i$ and ${\textbf {Z}}_i$, $i=1,\ldots , n$. $\square$

In other contexts, one may be interested in employing univariate test statistics of the form (1) to test a null hypothesis *H* against an alternative hypothesis *K*. Obviously, useful estimation of ${{\textbf {X}}}$ is only achievable if the distribution of ${{\textbf {X}}}_n - {{\textbf {X}}}$ is appropriately analyzed, and approximated if necessary. Likewise for the null distribution of a test statistic $X_n$ in the case of testing.

In the following, we focus on the common situation that the exact distribution of ${{\textbf {X}}}_n - {{\textbf {X}}}$ is unknown. The goal of this section is to determine the asymptotic distribution of the stochastic process $\sqrt{n}\big ({{\textbf {X}}}_n - {{\textbf {X}}}\big )$ for $n \rightarrow \infty$. A special feature of such counting process-based statistics is that they have a strong connection to martingales, and martingale theory can be used to analyze the asymptotic distribution. The connection to martingale theory is established by means of the Doob-Meyer decomposition, which links the $N_i$ uniquely to the martingale

3 $$\begin{aligned} M_i(t) = N_i(t) - \varLambda _i(t,\varvec{\beta }_0),\quad t\in \mathcal {T}, \end{aligned}$$

with respect to the filtration ${\mathcal{F}}_{1}(t) = \sigma\{ N_{i}(u), Y_{i}(u), {\textbf{Z}}_{i}(u), 0 \leq u \leq t, i=1,\ldots,n\}, t \in {\mathcal{T}}.$ The cumulative intensity process $\varLambda _i(t, \varvec{\beta }_0)$ as introduced in (3) is the compensator of $N_i(t)$, $t \in \mathcal {T}$; it is a non-decreasing predictable function in *t* with $\varLambda _i(0, \varvec{\beta }_0) = 0$, $i=1,\ldots ,n$. Additionally, we assume $\varLambda _i(t, \varvec{\beta }_0)$ to be absolutely continuous with intensity process $\lambda _i = {\displaystyle \frac{d}{dt}}\varLambda _i$ and expected value ${{\mathbb {E}}}(\varLambda _i(\tau , \varvec{\beta }_0)) < \infty$.

Note that we consider models for the intensity process which could also be expressed as ${\mathbb {E}}(d N_i (t) | \mathcal {F}_1(t-) ) = \lambda _i(t) dt$. We explicitly do not consider so-called rate (or marginal) models ${\mathbb {E}}(d N_i (t) | N_i(t-), Y_i(t), \varvec{Z}_i(u)) = \gamma _i(t) dt$, because they typically do not result in martingale structures; see, e.g., Scheike and Zhang (2003). In Sect. 5, we will address rate models in some more detail.

Some event times may be unobservable due to independent right-censoring, left-truncation, or more general incomplete data patterns such as independent censoring on intervals in the sense of [Andersen et al. 1993, Chapter III]. Technically, the covariate processes could also be expressed with the help of censored marked point processes; cf. [Andersen et al. (1993), Section III.5]. To mention one particular example: in the case of independent left-truncation and right-censoring, the underlying probability measure should be conditional on (some) event times being bigger than the study entry (or left-truncation) time. The censoring mechanisms are captured by the at-risk function $Y_i$, $i=1,\ldots ,n,$ and incorporated in the structure of the intensity process by assuming the multiplicative intensity model: $\lambda _i(t,\varvec{\beta }_0) = Y_i(t) \alpha _i(t,\varvec{\beta }_0), t\in \mathcal {T},$ where $\alpha _i ( \cdot ,\varvec{\beta }_0)$ is the hazard rate related to the events registered by the counting process $N_i$, and it does not depend on the censoring or the truncation. If a parametric or a semi-parametric model is chosen for the hazard rate, the hazard rate $\alpha _i ( t,\varvec{\beta }_0)$ could, for example, take the form $\alpha _0(t,\varvec{\beta }_{1;0} ) r(\varvec{\beta }_{2;0}^\top {{\textbf {Z}}}_i(t))$ or $\alpha _0(t ) r(\varvec{\beta }_0^\top {{\textbf {Z}}}_i(t))$, $t\in \mathcal {T}$, respectively, with $\varvec{\beta }_0 = (\varvec{\beta }_{1;0},\varvec{\beta }_{2;0})$ for parametric models. Here, $r(\cdot )$ is some relative risk function, $\alpha _0(\cdot ,\varvec{\beta }_{1;0})$ is the parametric baseline hazard function, and $\alpha _0(\cdot )$ is the nonparametric baseline hazard function. See Andersen et al. (1993) for a general reference for models based on counting processes.

We wish to find a useful asymptotic representation for $\sqrt{n} ({{\textbf {X}}}_n(t) - {{\textbf {X}}}(t))$. Before the introduction of the general version of this representation, we revisit the particular example of the Cox model.

#### Example 1c

*(Cox model continued)* For the Breslow estimator ${\hat{A}}_{0,n}(t, \varvec{\hat{\beta}}_n)$ it is well-known that, for $t\in \mathcal {T}$, $\sqrt{n} ({\hat{A}}_{0,n}(t, \varvec{\hat{\beta}}_n) - A_0(t))$ equals

4 $$\begin{aligned} \begin{aligned}&\frac{1}{\sqrt{n}} \sum _{i=1}^n \int _0^t \frac{nJ(u)}{S^{(0)}_n(u,\varvec{\beta }_0)} d M_i(u) - \frac{1}{{n}}\sum _{i=1}^n \int _0^t \frac{ nJ(u) {{\textbf {S}}}^{(1)}_n(u, \varvec{\beta }_0)^\top }{S^{(0)}_n(u,\varvec{\beta }_0)^2} dN_i(u)\\&\quad \times {{\textbf {C}}}_n\frac{1}{\sqrt{n}} \Big (\sum _{i=1}^n \int _0^\tau \Big ({{\textbf {Z}}}_i(u) - \frac{{{\textbf {S}}}^{(1)}_n(u, \varvec{\beta }_0)}{S^{(0)}_n(u, \varvec{\beta }_0)}\Big ) dM_i(u)\Big ) +o_p(1) , \end{aligned} \end{aligned}$$

where ${{\textbf {C}}}_n$ is a certain (random) $d\times d$ matrix. Note, in (4) it has been used that, for $t\in \mathcal {T}$, we have

$$\begin{aligned}&\sqrt{n}\Bigg ( \frac{1}{n} \sum _{i=1}^n \int _0^t \frac{nJ(u)}{S^{(0)}_n(u,\varvec{\beta }_0)} d \varLambda _i (u,\varvec{\beta }_0) - A_0(t)\Bigg ) = \sqrt{n} \int _0^t (J(u) - 1) d A_0(u) = o_p(1), \end{aligned}$$

and

$$\begin{aligned} \sqrt{n}(\varvec{\hat{\beta}}_n - \varvec{\beta }_0) = {{\textbf {C}}}_n\frac{1}{\sqrt{n}} \sum _{i=1}^n \int _0^\tau \Bigg ({{\textbf {Z}}}_i(u) - \frac{{{\textbf {S}}}^{(1)}_n(u, \varvec{\beta }_0)}{S^{(0)}_n(u, \varvec{\beta }_0)}\Bigg ) dM_i(u) + o_p(1), \end{aligned}$$

with $$\varvec{\hat{\beta}}_n - {\varvec{\beta }}_0 = O_p(n^{-1/2})$$. $$\square$$

Using Example 1c, we want to draw attention to common structures seen in the asymptotic representation of counting process-based estimators such as the Breslow estimator. That is, the asymptotic representation consists of four components: two scaled sums of martingale integrals, one scaled sum of counting process-based integrals and a matrix, ${{\textbf {C}}}_n$ in Example 1c, where each of the martingale integrals is formed with respect to a different integrand. In view of the similar structure of the two martingale integrals, we introduce the $(p+b)$-dimensional stochastic process ${\textbf {D}}_{n, h} = ({\textbf {D}}_{n, k}^\top ,{\textbf {D}}_{n, g}^\top )^\top$ which we define as

5 $$\begin{aligned} {{\textbf {D}}}_{n, h}(t) = \frac{1}{\sqrt{n}} \sum _{i=1}^n \int _0^t {{\textbf {h}}}_{n, i}(u, \varvec{\beta }_0) d M_i(u),\quad t\in \mathcal {T}, \end{aligned}$$

where the ${{\textbf {h}}}_{n, i}(t,\varvec{\beta } ) = ({{\textbf {k}}}_{n, i} (t,\varvec{\beta } )^\top , {{\textbf {g}}}_{n, i} (t,\varvec{\beta } )^\top )^\top : \varOmega \times \mathcal {T}\times \mathbb {R}^d \rightarrow \mathbb {R}^{p+b}$ are bounded stochastic processes that are predictable for $\varvec{\beta } = \varvec{\beta }_0$, $i=1,\ldots ,n$. Additionally, we define a $(p\times q)$-dimensional counting process-based integral ${{\textbf {B}}}_n(t)$ as

6 $$\begin{aligned} {{\textbf {B}}}_n(t) = \frac{1}{n} \sum _{i=1}^n \int _0^t \text {D} {{\textbf {k}}}_{n,i}(u, \varvec{\beta }_0) d N_i(u), \quad t \in \mathcal {T}, \end{aligned}$$

where $\text {D} {{\textbf {k}}}_{n,i}$ denotes the Jacobian of ${{\textbf {k}}}_{n,i}(t, \varvec{\beta })$ with respect to $\varvec{\beta }$.

In preparation of the presentation of the asymptotic representation of $\sqrt{n}\big ({{\textbf {X}}}_n - {{\textbf {X}}}\big )$, we make the following regularity assumption:

7 $$\begin{aligned} \frac{1}{n} \sum _{i=1}^n \int _0^t {{\textbf {k}}}_{n, i}(u, \varvec{\beta }_0) d \varLambda _i(u, \varvec{\beta }_0) - {{\textbf {X}}}(t) = o_p(n^{-1/2}) \text { for all } t\in \mathcal {T}. \end{aligned}$$

We also assume the following asymptotic representation for $\varvec{\hat{\beta}}_n$:

8 $$\begin{aligned} \sqrt{n}(\varvec{\hat{\beta }}_n-\varvec{\beta }_0) = {{\textbf {C}}}_n \frac{1}{\sqrt{n}} \sum _{i=1}^n \int _0^\tau {{\textbf {g}}}_{n, i}(u, \varvec{\beta }_0) d M_i(u) + o_p(1), \end{aligned}$$

where ${{\textbf {C}}}_n$ is some $(q\times b)$-dimensional random matrix and ${{\textbf {g}}}_{n, i}:\varOmega \times \mathcal {T}\times \mathbb {R}^q \rightarrow \mathbb {R}^b$ are bounded stochastic processes that are predictable for $\varvec{\beta } = \varvec{\beta }_0$, $i=1,\ldots ,n$. In Remark 1 at the end of this subsection, we illustrate why (8) is in general a natural condition for parametric models.

Now, we are ready to formulate the desired asymptotic representation for $\sqrt{n} ({{\textbf {X}}}_n(t) - {{\textbf {X}}}(t))$.

#### Lemma 1

If (2), (7), and (8) hold, then

9 $$\begin{aligned} \sqrt{n} ({{\textbf {X}}}_n(t) - {{\textbf {X}}}(t)) = {\textbf {D}}_{n, k}(t) +{{\textbf {B}}}_n(t) {{\textbf {C}}}_n{\textbf {D}}_{n, g}(\tau ) + o_p(1), \quad t\in \mathcal {T}. \end{aligned}$$

#### Example 1d

*(Cox model continued)* Using the notation introduced above, we can rewrite the asymptotic respresentation of the Breslow estimator given in (4) as $\sqrt{n} ({\hat{A}}_{0,n}(\cdot , \varvec{\hat{\beta}}_n) - A_0(\cdot )) = D_{n,k}(\cdot ) + {\textbf {B}}_n(\cdot ){{\textbf {C}}}_n {{\textbf {D}}}_{n,g}(\tau ) +o_p(1)$, where the martingale integrals of the first term, $D_{n,k}$, are formulated in terms of $k_{n}(t,\varvec{\beta }) = {\displaystyle {\frac{n J(t)}{S^{(0)}_n(t,\varvec{\beta })}}}$ at $\varvec{\beta }= \varvec{\beta }_0$, the martingale integrals involved in the second term, ${{\textbf {D}}}_{n,g}$, are formulated in terms of ${{\textbf {g}}}_{n,i}(t,\varvec{\beta }) ={\displaystyle { {{\textbf {Z}}}_i(t) - \frac{{{\textbf {S}}}^{(1)}_n(t,\varvec{\beta })}{S^{(0)}_n(t,\varvec{\beta })} }}$ at $\varvec{\beta }= \varvec{\beta }_0$, and the counting process-based integrals of the second term, ${\textbf {B}}_n$, involve $D k_{n}(t,\varvec{\beta }) = {\displaystyle - \frac{n J(t) {{\textbf {S}}}^{(1)}_n(t, \varvec{\beta })}{S^{(0)}_n(t,\varvec{\beta })^2}}$ at $\varvec{\beta }= \varvec{\beta }_0$. Note that the integrands ${{\textbf {g}}}_{n,i}(\cdot ,\varvec{\beta }_0)$ of ${{\textbf {D}}}_{n,g}$ as functions in *t* are bounded, because $S^{(0)}_n(\cdot ,\varvec{\beta }_0)^{-1}$ is bounded by $S^{(1)}_n(\cdot ,\varvec{\beta }_0)$ on ${\mathcal {T}}$ and $S^{(1)}_n(\cdot ,\varvec{\beta }_0)$ is bounded due to the boundedness of ${\textbf {Z}}_i$. Additionally, the integrands ${{\textbf {g}}}_{n,i}$ are predictable due to the predictability of $Y_i$ and ${\textbf {Z}}_i$, $i=1,\ldots , n$.$\square$

#### Remark 1

To illustrate that (8) is a natural condition, we note that, for parametric models, it is common practice to take the maximum likelihood estimator as the estimator $\varvec{\hat{\beta}}_n$ for estimating the true parameter $\varvec{\beta }_0$. In Borgan (1984), parametric survival models are considered, where for *n*-variate counting processes $(N_1, \dots , N_n)$ the likelihood equations are

$$\begin{aligned} \sum _{i=1}^n \int _0^\tau \nabla \alpha _i(u,\varvec{\beta }) \alpha _i(u,\varvec{\beta })^{-1} dN_i(u) - \sum _{i=1}^n \int _0^\tau \nabla \alpha _i(u,\varvec{\beta }) Y_i(u) du = 0, \end{aligned}$$

for some parametric functions $\alpha _i$, $i=1,\dots , n$, where $\nabla \alpha _i$ denotes the gradient of $\alpha _i$ with respect to $\varvec{\beta }$. Denote the left-hand side of the likelihood equations above by ${{\textbf {U}}}_n(\varvec{\beta },\tau )$. Then

$$\begin{aligned} {{\textbf {U}}}_n(\varvec{\beta }_0,t ) = \sum _{i=1}^n \int _0^t \frac{\nabla \alpha _i(u,\varvec{\beta }_0)}{\alpha _i(u,\varvec{\beta }_0)} dM_i(u), \quad t \in \mathcal {T} \end{aligned}$$

defines a square integrable martingale, as $\alpha _i(t,\varvec{\beta }_0) Y_i(t) dt = d\varLambda _i(t,\varvec{\beta }_0)$ is the compensator of $dN_i(t)$. Under regularity conditions a Taylor expansion of ${{\textbf {U}}}_n(\varvec{\hat{\beta}}_n,\tau )$ around $\varvec{\beta }_0$ yields

$$\sqrt{n}(\varvec{\hat{\beta}}_n - \varvec{\beta }_0 ) = -\Big (\frac{1}{n} D{{\textbf {U}}}_n(\varvec{\beta }_0,\tau )\Big )^{-1} \frac{1}{\sqrt{n}}{{\textbf {U}}}_n(\varvec{\beta }_0,\tau ) + o_p(1).$$

Thus, (8) holds where ${\displaystyle {{\textbf {g}}}_{n, i}(u, \varvec{\beta }_0) = \nabla \alpha _i(u,\varvec{\beta }_0) \alpha _i(u,\varvec{\beta }_0)^{-1}}$,

$$\displaystyle {{\textbf {C}}}_n = -\big (\frac{1}{n} D{{\textbf {U}}}_n(\varvec{\beta }_0,\tau )\big )^{-1},$$

with

$$D{{\textbf {U}}}_n(\varvec{\beta }_0,\tau ) = \sum _{i=1}^n \int _0^\tau \nabla ^2\log (\alpha _i(u,\varvec{\beta }_0)) dN_i(u) - \sum _{i=1}^n \int _0^\tau \nabla ^2 \alpha _i(u,\varvec{\beta }_0) Y_i(u) d u.$$

Note that $-\frac{1}{n} D{{\textbf {U}}}_n(\varvec{\beta }_0,\tau )$ is asymptotically equivalent to the optional covariation process $- \frac{1}{n} [{{\textbf {U}}}_n(\varvec{{\beta }}_0, \cdot )]$ of $-\frac{1}{\sqrt{n}}{{\textbf {U}}}_n(\varvec{{\beta }}_0, \cdot )$ at $\tau$, which will be of use in Remark 3. $\square$

In Sect. 2.3, we will focus on the derivation of the asymptotic distribution of the right-hand side of (9). Special attention is given to the martingale integrals ${\textbf {D}}_{n, h} = ({\textbf {D}}_{n, k}^\top ,{\textbf {D}}_{n, g}^\top )^\top$. According to Proposition II.4.1 of Andersen et al. (1993), ${{\textbf {D}}}_{n, h}$ is a square integrable martingale with respect to $\mathcal {F}_1$. Using this property, we will discuss in Sect. 2.2 an appropriate technique to proof the convergence in law of ${{\textbf {D}}}_{n, h}$ on $(D(\mathcal {T}))^{p+b}$, as $n\rightarrow \infty$. Here, $(D(\mathcal {T}))^{p+b}$ is the space of càdlàg functions in ${\mathbb {R}}^{p+b}$ equipped with the product Skorohod topology. Additionally, given a multi-dimensional vector of square integrable martingales ${{\textbf {H}}}_n(t), t \in \mathcal {T}$, the corresponding predictable and optional covariation processes are denoted by $\langle {{\textbf {H}}}_n \rangle (t)$ and $[{{\textbf {H}}}_n](t)$, respectively. Furthermore, we denote $\varvec{v}^{\otimes 2} = \varvec{v} \cdot \varvec{v}^\top \in \mathbb {R}^{l\times l}$ for some $\varvec{v}\in {\mathbb {R}}^{l}$, $\Vert \cdot \Vert$ will denote the Euclidean norm, and $\mathcal {B}$ a neighborhood of $\varvec{\beta }_0$.

### Rebolledo’s central limit theorem

To establish weak convergence results for the martingale components ${\textbf {D}}_{n,k}$ and ${\textbf {D}}_{n,g}$ of the martingale representation of $\varvec{X}_n$ given in (9), the use of a central limit theorem for martingales by Rebolledo (1980) seems the obvious choice. A variant of it, coined for applications in survival and event history analysis, is commonly used and propagated in textbooks, e.g., Andersen et al. (1993), Section II.5.1, and Aalen et al. (2008), Section 2.3.3. The stated requirements for the weak convergence of the martingales are the convergence of either of the predictable or optional variation processes. Additionally, it requires the convergence to zero of the predictable variation related to the process which only contains the jumps of the considered martingale that exceed an arbitrarily chosen $\epsilon> 0$. Here, the underlying assumption is that this $\epsilon$-jump process is a martingale. However, as we will now see, this underlying assumption does not hold. This is due to the integrand in the $\epsilon$-jump process which is not predictable.

#### Remark 2

To make this more explicit, consider the square-integrable zero-mean martingale $\varvec{D}_{n,\varvec{h}}(t) = \frac{1}{\sqrt{n}} \sum _{i=1}^n \int _0^t \varvec{h}_{n,i}(u, \varvec{\beta }_0) d M_i(u)$.

Let us for simplicity focus on any component of this multivariate martingale, say, ${D}_{n,{h}}(t) = \frac{1}{\sqrt{n}} \sum _{i=1}^n \int _0^t {h}_{n,i}(u, \varvec{\beta }_0) d M_i(u)$. Then the textbooks consider the stochastic process

$$D_{n,h}^\epsilon (t) = \int _0^t \mathbbm {1}\{|\varDelta D_{n,h}(u)|> \epsilon \} d D_{n,h}(u), \quad t \in {\mathcal {T}},$$

and its predictable variation process ought to converge to zero in probability. However, one may simplify the process as follows:

$$\begin{aligned} D_{n,h}^\epsilon (t) = \frac{1}{\sqrt{n}} \sum _{i=1}^n \int _0^t \mathbbm {1}\{| h_{n,i}(u)|> \sqrt{n} \epsilon \} h_{n,i}(u)dN_i(u), \quad t \in \mathcal {T}, \end{aligned}$$

since jumps are only due to jumps of the counting process and there are jumps of size 1 only. In contrast, the first display on p. 84 of Andersen et al. (1993) also involves a centering term in the integrator by subtracting the intensity process. However, our previous display reveals that $D_{n,h}^\epsilon (t)$ is not of the martingale form which is due to the non-predictability of the integrand $\mathbbm {1}\{|\varDelta D_{n,h}(u)|> \epsilon \}$. Consequently, it does not make sense to speak of the *predictable variation process* of this stochastic process. $\square$

The non-applicability of the variant of Rebolledo’s theorem as stated in the mentioned textbooks constitutes a gap in the literature that needs to be filled. In particular, the Lindeberg condition must be established with the help of another technique. To this end, we revisit the Lindeberg condition in (Rebolledo 1980) which requires the squared $\epsilon$-jump process to converge to zero in $\text {L}_1$, as $n\rightarrow \infty$. We combine this easily accessible Lindeberg condition with Rebolledo’s theorem for square integrable martingales by using the Lindeberg condition as a replacement for the rather technical ARJ(2) condition of that theorem; see also Proposition 1.5 of the same reference. For the sake of completeness, we now state this version of Rebolledo’s theorem.

#### Theorem 1

(Rebolledo’s martingale central limit theorem, Theorem V.1 of Rebolledo 1980) Let $H_n$ be a square integrable zero-mean martingale which satisfies the Lindeberg condition, i.e., for each $\epsilon>0$ and $t\in {\mathcal {T}}$,

10 $$\begin{aligned} \mathbb {E}(\sigma ^\epsilon [H_n](t)) = {\mathbb {E}}\Bigg ( \sum _{s\le t} (\varDelta H_n(s))^2 \mathbbm {1}\{|\varDelta H_n (s)|> \epsilon \}\Bigg )\rightarrow 0, \quad \text {as} \; n\rightarrow \infty . \end{aligned}$$

Consider the two following relations.

- $\langle H_n \rangle (t) {\mathop {\longrightarrow }\limits ^{\mathbb {P}}} V(t)$, as $n\rightarrow \infty$, for all $t \in \mathcal {T}$,
- $[ H_n ](t){\mathop {\longrightarrow }\limits ^{\mathbb {P}}} V(t)$, as $n\rightarrow \infty$, for all $t\in \mathcal {T}$.

If (a) (respectively (b)) holds, then relation (b) (respectively (a)) is also valid and

$$\begin{aligned} H_n {\mathop{\longrightarrow}\limits^{\mathcal{L}}} H, {\text{ in }} D({\mathcal{T}}), {\text{ as }} n\rightarrow \infty . \end{aligned}$$

Here, *H* denotes the 1-dimensional Gaussian centered continuous martingale with covariance function $\varSigma (s,t) = V(s \wedge t)$, $(s,t)\in {\mathcal{T}}^2$, where $V(t) = \langle H \rangle (t)$ is a continuous increasing real function with $V(0) = 0$.

We remark that the previous theorem considers one-dimensional martingales in the aforementioned paper. In contrast, we consider multi-dimensional martingales. To bridge this gap, one can make use of the Cramér–Wold theorem.

Although one may argue in a different way why the $\epsilon$-jumps of the martingale ${\textbf {D}}_{n,h}$ are asymptotically negligible and then draw conclusions for the convergence in law of ${\textbf {D}}_{n,h}$, it is of general interest to have Theorem 1 available as a broadly applicable solution that makes ad hoc workarounds superfluous.

### Regularity assumptions and weak convergence result

We are now ready to present the regularity conditions under which the asymptotic distribution of $\sqrt{n}(\varvec{X}_n-\varvec{X})$ can be established. In fact, the assumptions stated in Assumption 1 are required for the weak convergence of ${\textbf {D}}_{n, h}$ in the space of càdlàg functions, which is formulated in Lemma 5 in the Supporting Information.

#### Assumption 1

For each $i\in {\mathbb {N}}$ there exists a $(p+b)$-dimensional stochastic process $\tilde{{\textbf {h}}}_{i}(t,\varvec{\beta })$ defined on $\varOmega \times \mathcal {T}\times \mathcal {B}$ such that

- $\sup _{t\in \mathcal {T},i\in \{1,\ldots ,n\}}\Vert {\textbf {h}}_{n,i}(t,\check{\varvec{\beta }}_n) - \tilde{{\textbf {h}}}_{i}(t,\varvec{\beta }_0)\Vert {\mathop {\longrightarrow }\limits ^{{\mathbb {P}}}} 0,$ as $n\rightarrow \infty$, for a consistent estimator $\check{\varvec{\beta }}_n$ of $\varvec{\beta }_0$;
- $\tilde{{\textbf {h}}}_{i}(t,\cdot )$ is a continuous function in $\varvec{\beta } \in \mathcal {B}$ and bounded on $\mathcal {T}\times \mathcal {B}$;
- the $(p+b+1)$-tuples $(\tilde{{\textbf {h}}}_{i}(t,\varvec{\beta }_0),\lambda _i(t,\varvec{\beta }_0))$, $i=1,\ldots ,n$, are pairwise independent and identically distributed for all $t\in \mathcal {T}$.

Moreover, the following Assumption 2 is required to show a uniform weak law of large numbers for ${\textbf {B}}_n$; see Lemma 6 in the Supporting Information for details.

#### Assumption 2

For each $i\in {\mathbb {N}}$ there exists a $(p\times q)$-dimensional stochastic process $\tilde{{{\textbf {K}}}}_{i}(t,\varvec{\beta })$ defined on $\varOmega \times \mathcal {T}\times \mathcal {B}$ such that

- $\sup _{ t\in \mathcal {T},i\in \{1,\ldots ,n\}} \Vert \text {D} {{\textbf {k}}}_{n,i}(t,\check{\varvec{\beta }}_n) - \tilde{{{\textbf {K}}}}_{i}(t,\varvec{\beta }_0)\Vert {\mathop {\longrightarrow }\limits ^{\mathbb {P}}} 0$, as $n\rightarrow \infty$, for $\check{\varvec{\beta }}_n$ which is consistent for $\varvec{\beta }_0$;
- $\tilde{{{\textbf {K}}}}_{i} (\cdot ,\varvec{\beta }_0)$ is predictable w.r.t. $\mathcal {F}_1$ and bounded on $\mathcal {T}$;
- the $(p+q+1)$-tuples $(\text {vec}(\tilde{{\textbf {K}}}_{i}(t,\varvec{\beta }_0)),\lambda _i(t,\varvec{\beta }_0))$, $i=1,\ldots ,n$, are pairwise independent and identically distributed for all $t\in \mathcal {T}$.

Lastly, we state the convergence of ${\textbf {C}}_n$ as an additional assumption; the examples in Sect. 4 below demonstrate that this convergence is often implied by the above-stated assumptions.

#### Assumption 3

There exists ${{\textbf {C}}} \in \mathbb {R}^{q \times b}$ such that $\Vert {{\textbf {C}}}_n - {{\textbf {C}}}\Vert {\mathop {\longrightarrow }\limits ^{{\mathbb {P}}}} 0, \text { as } n\rightarrow \infty$.

Based on Lemma 1, the assumptions above, and Lemmas 5 and 6 in the Supporting Information, we finally obtain the desired weak convergence result for $\sqrt{n}(\varvec{X}_n-\varvec{X})$.

#### Theorem 2

If Lemma 1 holds and Assumptions 1, 2, and 3 are fulfilled, then,

$$\sqrt{n} \big ({\textbf {X}}_{n} - {\textbf {X}}\big )= {{\textbf {D}}}_{n, k} + {{\textbf {B}}}_n {{\textbf {C}}}_n {{\textbf {D}}}_{n, g}(\tau ) +o_p(1) {\mathop {\longrightarrow }\limits ^{\mathcal {L}}} {\textbf {D}}_{{\tilde{k}}} + {{\textbf {B}}} {{\textbf {C}}} {{\textbf {D}}}_{{\tilde{g}}}(\tau ), {\text{ in }} (D({\mathcal{T}}))^p,$$

as $n\rightarrow \infty$, with ${\textbf {D}}_{{\tilde{k}}}, {{\textbf {D}}}_{{\tilde{g}}}$, and ${{\textbf {B}}}$ given in Lemmas 5 and 6 in the Supporting Information. The limiting process is Gaussian.

Moreover, the matrix-valued (co)variance function of ${{\textbf {D}}}_{{\tilde{k}}} + {{\textbf {B}}} {{\textbf {C}}} {{\textbf {D}}}_{{\tilde{g}}}(\tau )$ is given as

$$t \mapsto {{\textbf {V}}}_{{\tilde{k}}}(t) + {{\textbf {B}}}(t) {{\textbf {C}}}{{\textbf {V}}}_{{\tilde{g}}}(\tau ) {{\textbf {C}}}^\top {{\textbf {B}}}(t)^\top +{{\textbf {V}}}_{{\tilde{k}},{\tilde{g}}}(t) {{\textbf {C}}}^\top {{\textbf {B}}}( t)^\top + {{\textbf {B}}}(t) {{\textbf {C}}} {{\textbf {V}}}_{{\tilde{g}}, {\tilde{k}}}(t).$$

Here, the covariance matrices ${{\textbf {V}}}_{{\tilde{k}}}(t) \in \mathbb {R}^{p \times p}, {{\textbf {V}}}_{{\tilde{g}}}(t) \in \mathbb {R}^{b \times b}, {{\textbf {V}}}_{{\tilde{k}},{\tilde{g}}}(t) \in \mathbb {R}^{p \times b}$, and ${{\textbf {V}}}_{{\tilde{g}},{\tilde{k}}}(t) \in \mathbb {R}^{b \times p}$, and ${{\textbf {B}}}(t) \in \mathbb {R}^{p \times q}$ are specified in Lemma 5 in the Supporting Information.

## Application of the wild bootstrap and a weak convergence result

As we are faced with the ignorance of the (asymptotic) distribution of ${{\textbf {X}}}_n - {{\textbf {X}}}$ in practical applications, we propose to employ the wild bootstrap as an approximation procedure. The wild bootstrap counterpart of ${{\textbf {X}}}_n$ will be denoted by ${{\textbf {X}}}_n^*$. In order to verify the validity of the approximation procedure, we will prove in Sect. 3.2 that, under regularity conditions, the (conditional) distributions of $\sqrt{n}({{\textbf {X}}}_n - {{\textbf {X}}})$ and $\sqrt{n}({{\textbf {X}}}_n^* - {{\textbf {X}}}_n)$ are asymptotically equivalent.

### The wild bootstrap estimator and its asymptotic representation

In order to define the wild bootstrap estimator ${{\textbf {X}}}_n^*$, we first introduce the core idea of the wild bootstrap. Naturally, the realisations of ${{\textbf {X}}}_n$ vary with the underlying data sets. If we had many data sets and thus many estimates, we could draw conclusions about the distribution of the estimator. The wild bootstrap provides for this: the variation immanent in the estimates arising from different data sets is produced by so-called random multipliers. In particular, the estimate calculated based on the available data set $({{\textbf {N}}}(t),{{\textbf {Y}}} (t),{{\textbf {Z}}} (t), t\in \mathcal {T})$ is perturbed by random multipliers such that for each realization of the random multiplier processes a new estimate is created. Based on these so-called wild bootstrap estimates, the distribution of the estimator can be inferred. Thus, the multiplier processes, denoted by $G_i(t)$, $t\in \mathcal {T}$, with $E(G_i{(t)}) = 0$, $E(G_i^2{(t)})=1$, and finite fourth moment, $i=1,\dots ,n$, lie at the heart of the wild bootstrap. They are random piecewise constant functions that we consider in further detail below. The construction of the wild bootstrap counterpart ${{\textbf {X}}}^*_n$ of ${{\textbf {X}}}_n$, ${{\textbf {B}}}^*_n$ of ${{\textbf {B}}}_n$, ${{\textbf {C}}}^*_n$ of ${{\textbf {C}}}_n$, ${{\textbf {D}}}^*_{n,h}$ of ${{\textbf {D}}}_{n,h}$, or of any of the quantities that arise in this context, can be attributed to the following replacements:

#### Replacement 1

- The square integrable martingale increment $dM_i(t)$ is replaced by the randomly perturbed counting process increment $G_i(t)dN_i(t)$, $i=1,\dots , n$;
- the unknown increments $\varLambda _i(dt,\varvec{\beta }_0)$ are replaced by $dN_i(t)$, $i=1,\ldots ,n$;
- the unknown parameter coefficient $\varvec{\beta }_0$ is replaced by the estimator $\varvec{\hat{\beta}}_n$;
- we set all $o_p(1)$ terms in asymptotic representations to 0.

$$\square$$

Note that the substitution $G_i(t) dN_i(t)$ of $dM_i(t)$, $t\in \mathcal {T}$, in Replacement 1 (a) is a square integrable martingale increment itself, given the data set, cf. Lemma 3 below. Moreover, for a broader applicability, we chose in Replacement 1 (b) the nonparametric estimator $dN_i(t)$ rather than a semiparametric estimator $\hat{\varLambda }_i(dt,\varvec{\hat{\beta}}_n)$, $t\in \mathcal {T}$. As a consequence of Replacement 1, we also replace the counting process increments $dN_i(t)$ and the estimator $\varvec{\hat{\beta}}_n$ in two steps. In case of the counting process increments, we decompose $dN_i(t)$ into $dM_i(t) + d\varLambda _i(t,\varvec{\beta }_0)$ according to the Doob-Meyer decomposition given in (3). Second, Replacement 1 (a) and (b) are applied. Step one and two combined yield

$$\big (G_i(t) + 1 \big ) dN_i(t), t\in \mathcal {T}$$

as the replacement for $dN_i$. Furthermore, we obtain a wild bootstrap counterpart of $\varvec{\hat{\beta}}_n$ via its asymptotic representation given in (8). According to that equation, we have

11 $$\begin{aligned} \varvec{\hat{\beta}}_n = \varvec{\beta }_0 + {{\textbf {C}}}_n\frac{1}{{n}}\sum _{i=1}^n \int _0^\tau {\textbf {g}}_{n,i}(u,{\varvec{\beta }}_0) dM_i(u) \ + \ o_p(1). \end{aligned}$$

In order to define the wild bootstrap counterpart $\varvec{\hat{\beta}}^*_n$ of $\varvec{\hat{\beta}}_n$, we replace ${{\textbf {C}}}_n$ by some $(q\times b)$-dimensional random matrix ${{\textbf {C}}}_n^*$ which is a wild bootstrap counterpart of ${{\textbf {C}}}_n$, and apply Replacement 1 to the other terms on the right hand side of (11). This yields

12 $$\begin{aligned} \varvec{\hat{\beta}}^*_n = \varvec{\hat{\beta}}_n + {{\textbf {C}}}_n^* \frac{1}{{n}} \sum _{i=1}^n \int _0^\tau {\textbf {g}}_{n,i}(u,\varvec{\hat{\beta}}_n) G_i(u)dN_i(u). \end{aligned}$$

Note that ${{\textbf {C}}}_n^*$ could take many different forms as long as it is asymptotically equivalent to ${{\textbf {C}}}_n$, i.e., as long as $\Vert {{\textbf {C}}}_n^* - {{\textbf {C}}}_n \Vert = o_p(1)$ holds for $n\rightarrow \infty$, cf. Assumption 4. When working with a particular model, a natural choice for ${{\textbf {C}}}_n^*$ might be apparent; cf. Remark 3.

We now consider the multiplier processes $G_i(t)$, $t\in \mathcal {T}, i=1,\ldots ,n,$ in more detail. We define $G_i$ as a random, piecewise constant function with jumps simultaneous with $N_i$, i.e., at

13 $$\begin{aligned} \mathcal {T}^\varDelta _{n,i} = \{t \in \mathcal {T}: \varDelta N_i(t) = 1\} = \{T_{i,1},\ldots ,T_{i,n_i}\}. \end{aligned}$$

We note that the number of jumps for the *i*-th process is the random number $n_i = N_i(\tau ) \ge 0$. 

Indeed, addressing a reviewer’s comments, we would like to point out the consequences of using time-constant multiplier processes for resampling Nelson–Aalen estimator $\displaystyle {\hat{A}}_n(t) = \sum \nolimits _{i=1}^n \int _0^t \frac{J(u)dN_i(u)}{Y(u)}$ when $N_i$ may have multiple events per individual, $N_i(\tau ) \in \mathbb {N}$.

Moreover﻿, the multiplier processes $G_i$ are constructed such that at the jump time points $T_{i,j} \in \mathcal {T}^\varDelta _{n,i}$ they take the values of i.i.d. random variables $G_{i,j}$, $j=1,2,\ldots$, that have mean zero, unit variance and finite fourth moment, and that are independent of $\mathcal {F}_1(\tau )$. In particular, $G_i(t)= 0$ for $t < T_{i,1}$ and $G_i(t)= G_{i,j}$ for $T_{i,j} \le t < T_{i,j+1}$, where $T_{i, n_i+1} = \infty$. As an example for possible distributions of $G_{i,j}$, we refer to the simulation studies of related articles in which $G_{i,j}\sim \text {Poi}(1) - 1$, $G_{i,j}\sim \text {Exp}(1) - 1$, and $G_{i,j}\sim \text {N}(0,1)$ have been investigated; see, e.g., Beyersmann et al. (2013) and Dobler et al. (2019). Furthermore, the multiplier processes $G_1(t),\ldots ,G_n(t)$, $t\in \mathcal {T}$, are pairwise independent and identically distributed. Conditionally on $\mathcal {F}_1(\tau )$, however, their jump times are fixed and the identical distribution is lost. See Bluhmki et al. (2018, 2019) for similar approaches.

By applying Replacement 1 to ${\textbf {X}}_n$, we arrive at the wild bootstrap counterpart ${\textbf {X}}_n^*$:

14 $$\begin{aligned} {\textbf {X}}^*_n (t) = \frac{1}{n} \sum _{i=1}^n \int _0^t {\textbf {k}}_{n,i}(u,\varvec{\hat{\beta}}^*_n)\big (G_i(u)+1 \big ) dN_i(u), \quad t\in \mathcal {T}. \end{aligned}$$

The remaining part of this section concerns the asymptotic behaviour of the wild bootstrap estimator ${\textbf {X}}^*_n$ around ${{\textbf {X}}}_n$. In this context, we are interested in a representation of $\sqrt{n} ({{\textbf {X}}}_n^* - {{\textbf {X}}}_n )$ which is analogous to the asymptotic representation of $\sqrt{n} ({{\textbf {X}}}_n - {{\textbf {X}}} )$ given (9). For illustrative purposes, we start by returning to the example of the Cox model.

#### Example 1e

*(Cox model continued)* Recall from Example 1a that the Breslow estimator is given by

$${\hat{A}}_{0,n}(t, \varvec{\hat{\beta}}_n) = \frac{1}{n}\sum _{i=1}^n \int _0^t \frac{nJ(u)}{S^{(0)}_n(u,\varvec{\hat{\beta}}_n)} dN_i(u), t\in \mathcal {T}.$$

According to (14), the wild bootstrap counterpart ${\hat{A}}^*_{0,n}$ of ${\hat{A}}_{0,n}$ is obtained by applying Replacement 1 to the Breslow estimator. That is,

$${\hat{A}}^*_{0,n}(t, \varvec{\hat{\beta}}_n^*) = \frac{1}{n}\sum _{i=1}^n \int _0^t \frac{nJ(u)}{S^{(0)}_n(u,\varvec{\hat{\beta}}_n^*)} (G_i(u) + 1)dN_i(u), t\in \mathcal {T}.$$

A Taylor expansion of $k_{n}(t,\varvec{\hat{\beta}}_n^*)$ around $\varvec{\hat{\beta}}_n$ yields that $\sqrt{n} ({\hat{A}}^*_{0,n}(t, \varvec{\hat{\beta}}^*_n) - {\hat{A}}_{0,n}(t, \varvec{\hat{\beta}}_n))$ equals

15 $$\begin{aligned} \begin{aligned}&\frac{1}{\sqrt{n}} \sum _{i=1}^n \int _0^t \frac{nJ(u) }{S^{(0)}_n(u,\varvec{\hat{\beta}}_n)}G_i(u)\,d N_i(u) \\&\quad - \frac{1}{n} \sum _{i=1}^n\int _0^t \frac{n J(u) {{\textbf {S}}}^{(1)}_n(u, \varvec{\hat{\beta}}_n)}{S^{(0)}_n(u,\varvec{\hat{\beta}}_n)^2}(G_i(u) + 1) dN_i(u)\\&\quad \times {{{\textbf {C}}}}^{*}_n \frac{1}{\sqrt{n}} \Big (\sum _{i=1}^n \int _0^\tau \Big ({{\textbf {Z}}}_i(u) - \frac{{{\textbf {S}}}^{(1)}_n(u, \varvec{\hat{\beta}}_n)}{S^{(0)}_n(u, \varvec{\hat{\beta}}_n)}\Big ) G_i(u)\,dN_i(u)\Big ) +o_p(1), \quad t\in \mathcal {T} \end{aligned} \end{aligned}$$

where $$\sqrt{n}(\varvec{\hat{\beta}}_n^* - \varvec{\hat{\beta}}_n) = {{{\textbf {C}}}}_n^*\frac{1}{\sqrt{n}} \sum _{i=1}^n \int _0^\tau \Big ({{\textbf {Z}}}_i(u) - \frac{{{\textbf {S}}}^{(1)}_n(u, \varvec{\hat{\beta}}_n)}{S^{(0)}_n(u,\varvec{\hat{\beta}}_n)}\Big ) G_i(u)dN_i(u)$$ with $$o_p(\varvec{\hat{\beta}}^*_n - \varvec{\hat{\beta}}_n) = o_p(n^{-1/2})$$. $$\square$$

Using the example of the Cox model, we draw attention to common structures seen in the asymptotic representation of the wild bootstrap counterpart of counting process-based estimators such as ${\hat{A}}^*_{0,n}(\cdot , \varvec{\hat{\beta}}^*_n)$ compared to the asymptotic representation of the counting process-based estimators such as ${\hat{A}}_{0,n}(\cdot , \varvec{\hat{\beta}}_n)$. In particular, Replacement 1 transforms the components given in (4) into their counterparts seen in (15): the two scaled sums of martingale integrals are transformed into scaled sums of integrals with respect to the randomly perturbed counting process increments $G_idN_i$, the scaled sum of counting process-based integrals into the scaled sum of integrals with respect to $(G_i(u) + 1) dN_i(u)$, and the matrix ${{{\textbf {C}}}}_n$ is replaced by a wild bootstrap counterpart denoted by ${{{\textbf {C}}}}_n^*$. Additionally, the integrands of all integrals given in (15) are now evaluated at $\varvec{\hat{\beta}}_n$ instead of at ${\varvec{\beta }_0}$.

As a preparation for the generalization of these observations to the representation of $\sqrt{n} ({{\textbf {X}}}_n^* - {{\textbf {X}}}_n )$, we introduce the wild bootstrap counterpart ${\textbf {D}}^*_{n,h} = ({\textbf {D}}^*_{n,k},{\textbf {D}}^*_{n,g})$ of ${\textbf {D}}_{n,h} = ({\textbf {D}}_{n,k},{\textbf {D}}_{n,g})$. In particular, applying Replacement 1 to ${\textbf {D}}_{n,h}(t) = \frac{1}{\sqrt{n}} \sum _{i=1}^n \int _0^t {{\textbf {h}}}_{n, i}(u, \varvec{\beta }_0) d M_i(u)$, $t\in \mathcal {T}$, yields

16 $$\begin{aligned} {\textbf {D}}^*_{n,h} (t) = \frac{1}{\sqrt{n}}\sum _{i=1}^n \int _0^t {\textbf {h}}_{n,i}(u,\varvec{\hat{\beta}}_n) G_i(u) dN_i(u),\quad t\in \mathcal {T}, \end{aligned}$$

where again ${{\textbf {h}}}_{n,i} = ({{\textbf {k}}}_{n,i}^\top , {{\textbf {g}}}_{n,i}^\top )^\top$. We assume that ${{\textbf {h}}}_{n,i}(t,\varvec{\hat{\beta}}_n)$, $t\in \mathcal {T}$, is a known, $\mathcal {F}_1(\tau )$-measurable $(p+b)$-dimensional function. Additionally, we establish the wild bootstrap version ${{\textbf {B}}}_n^*(t)$ of ${{\textbf {B}}}_n (t) = \frac{1}{n} \sum _{i=1}^n \int _0^t \text {D} {{\textbf {k}}}_{n,i}(u, \varvec{\beta }_0) d N_i(u)$, $t\in \mathcal {T}$, that is,

17 $$\begin{aligned} {{\textbf {B}}}_n^*(t) =\frac{1}{n} \sum _{i=1}^n \int _0^t \text {D} {{\textbf {k}}}_{n,i}(u, \varvec{\hat{\beta}}_n) ( G_i(u) + 1 ) dN_i(u), t\in \mathcal {T}, \end{aligned}$$

which is obtained by applying Replacement 1 to ${{\textbf {B}}}_n$. The following lemma reveals the desired representation for $\sqrt{n}\big ({{\textbf {X}}}_n^*(t) - {{\textbf {X}}}_n(t) \big )$ as a counterpart to the expression given for $\sqrt{n}\big ({{\textbf {X}}}_n(t) - {{\textbf {X}}}(t) \big )$ in Lemma 1.

#### Lemma 2

If (12) holds with $o_p(\varvec{\hat{\beta}}^*_n - \varvec{\hat{\beta}}_n) = o_p(n^{-1/2})$, then

18 $$\begin{aligned} \sqrt{n}\big ({{\textbf {X}}}_n^*(t) - {{\textbf {X}}}_n(t) \big ) = {\textbf {D}}^*_{n,k} (t ) + {{\textbf {B}}}_n^*(t){{\textbf {C}}}_n^* {\textbf {D}}^*_{n,g}(\tau )+ o_p(1), t \in \mathcal {T}. \end{aligned}$$

Indeed, as we will see later, $\varvec{\hat{\beta}}^*_n - \varvec{\hat{\beta}}_n = O_p(n^{-1/2})$. Hence, $o_p(\varvec{\hat{\beta}}^*_n - \varvec{\hat{\beta}}_n) = o_p(n^{-1/2})$.

#### Example 1f

*(Cox model continued)* For the Cox model the scaled and centered wild bootstrap estimator ${\hat{A}}^*_{0,n}$ can be written in the general form presented in Lemma , that is,

$$\sqrt{n} ({\hat{A}}^*_{0,n}(\cdot , \varvec{\hat{\beta}}^*_n) - {\hat{A}}_{0,n}(\cdot , \varvec{\hat{\beta}}_n)) = {\textbf {D}}^*_{n,k} (\cdot ) + {{\textbf {B}}}_n^*(\cdot ){{\textbf {C}}}_n^* {\textbf {D}}^*_{n,g}(\tau )+ o_p(1),$$

where the integrands $k_n(\cdot, \varvec{{\beta}})$, $\textbf g_{n,i}(\cdot,  \varvec{{\beta}})$, and $ D k_{n}(\cdot,\varvec{{\beta}})$ of $\textbf{D}^*_{n,k}$, $\textbf{D}^*_{n,g}$, and $\textbf{B}^*_n$, respectively, each evaluated at $ {\varvec{\beta}} = \varvec{\hat{\beta}}_n$, are given in Example 1d. $$\square$$

In the upcoming Sect. 3.2, we make use of the following notation. Given a multi-dimensional vector of square integrable martingales ${{\textbf {H}}}_n(t), t \in \mathcal {T}$, $\mathcal {L}({{\textbf {H}}}_n)$ and $\mathcal {L}({{\textbf {H}}}_n|\cdot )$ denote the law and the conditional law of ${{\textbf {H}}}_n$, respectively. Additionally, let $d[\cdot ,\cdot ]$ be the Prohorov distance between probability distributions.

### Regularity assumption and weak convergence result

In order to prove that the proposed resampling procedure results in the correct limiting distribution, we wish to exploit martingale theory. This requires the definition of a filtration which captures the evolving randomness due to the multipliers but not due to the original sample. The latter is considered to be fixed from the resampling point of view. In other words, we search a filtration that includes (i) all available data at time zero , that is, $\mathcal {F}_1(\tau )$; (ii) the values of the wild bootstrap multiplier processes $G_i$ during the course of time. This leads us to

$$\mathcal {F}_2(t) =\sigma \{G_i(s), N_i(u), Y_i(u), {{\textbf {Z}}}_{i}(u), 0<s\le t, u\in \mathcal {T}, i=1,\ldots ,n\},$$

$t\in \mathcal {T}.$ Note that $\mathcal {F}_2(0) = \mathcal {F}_1(\tau )$ represents the available data. From now on, the underlying filtered probability space is $(\varOmega ,\mathcal {A}, \mathcal {F}_2{,\mathbb {P}})$. Moreover, we identify ${\textbf {D}}^*_{n,h}$ as a square integrable martingale with respect to the proposed filtration and state its predictable and optional variation process.

#### Lemma 3

${\textbf {D}}_{n,h}^*$ is a square integrable martingale with respect to $\mathcal {F}_2$. Its predictable and optional covariation processes are

$$\begin{aligned} \langle {\textbf {D}}^*_{n,h} \rangle (t)= \frac{1}{n}\sum _{i=1}^n\int _0^t {\textbf {h}}_{n,i}(u,\varvec{\hat{\beta}}_n)^{\otimes 2}\, dN_i(u), \ t\in \mathcal {T}, \end{aligned}$$

and

$$\begin{aligned} \left[{\textbf {D}}^*_{n,h}\right] (t)= \frac{1}{n}\sum _{i=1}^n\int _0^t {\textbf {h}}_{n,i}(u,\varvec{\hat{\beta}}_n)^{\otimes 2} G^2_i(u) \, dN_i(u), \ t\in \mathcal {T}, \end{aligned}$$

respectively.

According to Lemma 3, a martingale central limit theorem could be used to derive the asymptotic distribution of ${\textbf {D}}_{n,h}^*$. Along the lines of Sect. 2.2, we examine the applicability of Rebolledo’s martingale central limit theorem as stated in Theorem II.5.1 of Andersen et al. (1993). Particular attention is paid to the applicability of the corresponding Lindeberg condition. Recall that the process ${\textbf {D}}_{n,h}^*$ is a martingale based on integrals with respect to the randomly perturbed counting processes $G_iN_i$ with general multipliers $G_i$, $i=1,\ldots ,n$. Due to the general, possibly asymmetric multipliers, the Lindeberg condition of Rebolledo’s theorem as stated in the aforementioned textbook is again not applicable. This is, as the following example demonstrates, because the $\epsilon$-jump process of ${\textbf {D}}^*_{n,h}$ is in general not a martingale. As a consequence, we cannot apply Theorem II.5.1 of Andersen et al. (1993), as it only makes sense to speak of the predictable covariation process of a martingale. For symmetrically distributed multipliers, however, the version of Rebolledo’s theorem in Andersen et al. (1993) would still be applicable to the bootstrapped quantities.

#### Example 2

We consider the case where $N_i \le 1$ and a square integrable martingale with integrand $h_{n, i}(t, \hat{{\beta }})\equiv 1$, i.e., $D_{n,h}^*(t) = \frac{1}{\sqrt{n}} \sum _{i=1}^n \int _0^t G_i(u) dN_i(u)$, $t\in \mathcal {T}$. That is, for the $\epsilon$-jump process $D_{n,h}^{\epsilon ,*}(t) = \int _0^t \mathbbm {1}\{|\varDelta D_{n,h}^*(u)|\ge \epsilon \} D_{n,h}^*(du)$,

$$\begin{aligned}&{\mathbb {E}} (D_{n,h}^{\epsilon ,*}(t)|\mathcal {F}_2(s)) \\&\quad = {\mathbb {E}} \Bigg ( \frac{1}{\sqrt{n}} \sum _{i=1}^n \int _0^t \mathbbm {1}\Bigg \{\Bigg |\frac{1}{\sqrt{n}}\sum _{i=1}^n G_i(u) \varDelta N_i(u) \Big |\ge \epsilon \Bigg \} G_i(u) dN_i(u)\Bigg |\mathcal {F}_2(s)\Bigg )\\&\quad = D_{n,h}^{\epsilon ,*}(s) + \frac{1}{\sqrt{n}} \sum _{i=1}^n \int _s^t {\mathbb {E}} \Bigg ( \mathbbm {1}\Bigg \{\Bigg |\frac{1}{\sqrt{n}}\sum _{i=1}^n G_i(u) \varDelta N_i(u)\Bigg |\ge \epsilon \Bigg \} G_i(u) \Bigg |\mathcal {F}_2(s)\Bigg )dN_i(u)\\&\quad = D_{n,h}^{\epsilon ,*}(s) + \frac{1}{\sqrt{n}} \sum _{i=1}^n {\mathbb {E}} \Bigg ( \mathbbm {1}\Bigg \{\Bigg |\frac{1}{\sqrt{n}} G_{i,1} \Bigg |\ge \epsilon \Bigg \} G_{i,1}\Bigg ) (N_i(t) - N_i(s)) , \quad t\in \mathcal {T}, \end{aligned}$$

which is in general not equal to $D_{n,h}^{\epsilon ,*}(s)$ if the zero mean random variables $G_{1,1},\ldots ,G_{n,1}$ follow an asymmetric distribution. Hence, $D_{n,h}^{\epsilon ,*}(t)$, $t\in \mathcal {T}$, does not fulfill the martingale property in the considered setting in which the multiplier processes may follow a general, possibly asymmetric distribution with zero mean, unit variance, and finite fourth moment, such as the centered exponential distribution or the centered Poisson distribution. However, if the multipliers follow a symmetric distribution, the martingale property does hold, as the last expectation in the previous display vanishes. $\square$

In conclusion, to prove the convergence in distribution of ${\textbf {D}}_{n,h}^*$, we have to resort to the widely applicable version of Rebolledo’s martingale central limit theorem as given in Theorem 1, albeit a conditional version thereof. The corresponding weak convergence result for ${\textbf {D}}_{n,h}^*$ is presented in Lemma 8 in the Supporting Information.

#### Assumption 4

Under Assumption 3, we further assume that the $(q\times b)$-dimensional random matrices ${{\textbf {C}}}_n$ and ${{\textbf {C}}}_n^*$ are asymptotically equivalent, $\Vert {{\textbf {C}}}_n^* - {{\textbf {C}}}_n \Vert {\mathop {\longrightarrow }\limits ^{{\mathbb {P}}}} 0$, as $n \rightarrow \infty .$

We are ready to state our main theorem about the asymptotic distribution of $\sqrt{n}({{\textbf {X}}}_n^* - {{\textbf {X}}}_n)$ conditional on the data.

#### Theorem 3

If Lemma 2 holds, Assumptions 1, 2, 3, and 4 imply

$$\begin{aligned} \sqrt{n} \big ({\textbf {X}}^*_{n} - {\textbf {X}}_n\big )= {{\textbf {D}}}^*_{n, k} + {{\textbf {B}}}^*_n {{\textbf {C}}}^*_n {{\textbf {D}}}^*_{n, g}(\tau ) +o_p(1) {\mathop {\longrightarrow }\limits ^{\mathcal {L}}} {\textbf {D}}_{{\tilde{k}}} + {{\textbf {B}}} {{\textbf {C}}} {{\textbf {D}}}_{{\tilde{g}}}(\tau ), \text { in } (D(\mathcal {T}))^p, \end{aligned}$$

conditionally on $\mathcal {F}_2(0)$ in probability, as $n\rightarrow \infty$, with ${\textbf {D}}_{{\tilde{k}}}, {{\textbf {D}}}_{{\tilde{g}}}$, and ${{\textbf {B}}}$ as stated in the Supporting Information, respectively. If also Lemma 1 holds , we have, as $n\rightarrow \infty ,$

$$d[\mathcal {L}(\sqrt{n}({\textbf {X}}_{n}^*-{\textbf {X}}_{n})|\mathcal {F}_2(0)),\mathcal {L}(\sqrt{n}({\textbf {X}}_{n}-{\textbf {X}}))]{\mathop {\longrightarrow }\limits ^{{\mathbb {P}}}} 0.$$

In conclusion, Theorem 3 establishes the asymptotic validity of the wild bootstrap for approximations of the distribution of counting process-based statistics of the form (1).

#### Remark 3

We continue Remark 1 in order to illustrate how to choose the wild bootstrap counterpart ${{\textbf {C}}}_n^*$ of ${{\textbf {C}}}_n$ in parametric survival models such that Assumption 4 holds. In this way, we underline the wild bootstrap as an alternative to the parametric bootstrap. As stated in Remark 1, ${{\textbf {C}}}_n$ is asymptotically related to the optional covariation process $\frac{1}{n}[{{\textbf {U}}}_n(\varvec{\beta }_0, \cdot )]$ of $\frac{1}{\sqrt{n}}{{\textbf {U}}}_n(\varvec{\beta }_0, \cdot )$. Hence, we propose to choose ${{\textbf {C}}}_n^*$ similarly based on the optional covariation process $\frac{1}{n}[{{\textbf {U}}}_n^*(\varvec{\hat{\beta}}_n, \cdot )]$ of the wild bootstrap version $\frac{1}{\sqrt{n}}{{\textbf {U}}}_n^*(\varvec{\hat{\beta}}_n, \cdot )$ of the martingale $\frac{1}{\sqrt{n}}{{\textbf {U}}}_n(\varvec{\beta }_0, \cdot )$. Application of Replacement 1 to $\frac{1}{\sqrt{n}}{{\textbf {U}}}_n(\varvec{\beta }_0, \cdot )$ yields

$${{\textbf {D}}}_{n,g}^*(\tau ) = \frac{1}{\sqrt{n}}{{\textbf {U}}}_n^*(\varvec{\hat{\beta}}_n, \tau ) = \frac{1}{\sqrt{n}}\sum _{i=1}^n \int _0^\tau \frac{\nabla \alpha _i(u,\varvec{\hat{\beta}}_n)}{\alpha _i(u,\varvec{\hat{\beta}}_n)}G_i(u) dN_i(u).$$

According to Lemma 3 we obtain the following structure:

$${{\textbf {C}}}_n^* = \Bigg (-\frac{1}{n} [{{\textbf {U}}}_n^*(\varvec{\hat{\beta}}_n, \cdot )](\tau )\Bigg )^{-1} = -\Bigg (\frac{1}{n} \sum _{i=1}^n \int _0^\tau \frac{(\nabla \alpha _{i}(u,\varvec{\hat{\beta}}_n))^{\otimes 2}}{\alpha _{i}(u,\varvec{\hat{\beta}}_n)^2} G_i^2(u)dN_i(u) \Bigg )^{-1}.$$

This is a natural choice for ${{\textbf {C}}}_n^*$ because the (conditional) distributions of ${{\textbf {D}}}_{n,g}^*$ and ${{\textbf {D}}}_{n,g}=\frac{1}{\sqrt{n}}{{\textbf {U}}}_n(\varvec{\beta }_0, \cdot )$ are asymptotically equivalent and the same holds for their optional covariation processes; cf. the Supporting Information. $\square$

#### Example 1g

*(Cox model, conclusion)*

Lastly, the assumptions required in Theorem 3 are considered in the context of the Cox model and final conclusions are drawn. First, the integrands introduced below Lemma 1 are examined. The uniform limits in probability of $k_{n}$ and ${{\textbf {g}}}_{n,i}$ are $\tilde{k} ={\displaystyle \frac{1}{s^{(0)}}}$ and $\tilde{{{\textbf {g}}}}_i = {{\textbf {Z}}}_i - {\displaystyle \frac{s^{(1)}}{s^{(0)}}}$, respectively, where $s^{(j)}$ are the uniform deterministic limits in probability of $n^{-1} S_n^{(j)}$, $j=0,1$. Under the typically made assumptions (Condition VII.2.1 of Andersen et al. 1993) and under the assumption that the covariate vectors ${{\textbf {Z}}}_i$, $i=1,\ldots ,n$, are pairwise independent and identically distributed, Assumption 1 is fulfilled. Similarly, the uniform limit in probability of $D k_{n}$ is $\tilde{K} = {\displaystyle \frac{s^{(1)}}{(s^{(0)})^2}}$. Next, the matrices ${\textbf {B}}_n$ and ${\textbf {C}}_n$ are considered, where the explicit form of ${\textbf {C}}_n$ for the Cox model is $\big [\frac{1}{n}\sum _{i=1}^n \int _0^\tau \big ( \frac{S^{(2)}_n(u,\varvec{\beta }_0)}{S^{(0)}_n(u,\varvec{\beta }_0)} - \big ( \frac{S^{(1)}_n(u,\varvec{\beta }_0)}{S^{(0)}_n(u,\varvec{\beta }_0)}\big )^{\otimes 2} \big )dN_i(u)\big ]^{-1}.$ Again, under Condition VII.2.1 and (7.2.28) of Andersen et al. (1993), Assumptions 2 and 3 are valid. The wild bootstrap counterpart ${{\textbf {C}}}^*_n$ of ${{\textbf {C}}}_n$ as given in Remark 3 simplifies for the Cox model to ${{\textbf {C}}}^*_n = \big [\frac{1}{n}\sum _{i=1}^n \int _0^\tau \big ( \frac{S^{(2)}_n(u,\varvec{\hat{\beta}})}{S^{(0)}_n(u,\varvec{\hat{\beta}})} - \big ( \frac{S^{(1)}_n(u,\varvec{\hat{\beta}})}{S^{(0)}_n(u,\varvec{\hat{\beta}})}\big )^{\otimes 2} \big ) G_i(u)^2 dN_i(u)\big ]^{-1}.$ Assumption 4 is satisfied as argued in Remark 3. Moreover, Lemmas 1 and 2 hold according to Examples 1c and 1e, respectively. Finally, Theorem 3 can be applied to verify the asymptotic validity of the wild bootstrap for statistical inference on the Breslow estimator. $\square$

## Examples

Additionally to Examples 1a–1g covering the Cox model, we will now present two additional examples which further illustrate specific cases of the general set-up described in Sects. 2 and 3. In particular, it is briefly outlined how the theory developed in this paper can be applied to these models. In a related paper (Dietrich et al. 2023), the present approach is applied to the estimators involved in the Fine–Gray model under censoring-complete data and the details of the wild bootstrap for the corresponding cumulative incidence function is worked out.

### Example 3

*(Nelson–Aalen estimator)* Let $X(t)= A(t) = \int _0^t \alpha (u) du$, $t \in \mathcal {T}$, be the cumulative hazard function of a continuous survival time *T*, i.e., $\alpha (u)du = \mathbb {P}(T \in [u,u + du] | T \ge u)$. Let $N_1(t), \dots , N_n(t)$, $t\in \mathcal {T}$, be the counting processes that are related to *n* independent copies of *T* which possibly involve right-censoring. For ${\hat{X}}_n(t)$, $t \in \mathcal {T}$, we take the Nelson–Aalen estimator ${\hat{A}}_n(t) = {\displaystyle \sum _{i=1}^n \int _0^t } {\displaystyle \frac{J(u) }{Y(u)}}d N_i(u)$, $t\in \mathcal {T}$, Aalen (1978), where $Y_i(t)$ is the at-risk indicator for individual *i* at time *t*, $Y(t)= \sum _{i=1}^n Y_i(t)$, and $J(t) = \mathbbm {1}\{Y(t)> 0 \}$. Thus, the counting process-based estimator ${\hat{A}}_n$ exhibits the general structure stated in (1) with $k_{n}(t) = \tfrac{n J(t)}{Y(t)}$, $t\in \mathcal {T}$. Furthermore, we have for $t\in \mathcal {T}$,

19 $$\begin{aligned} \sqrt{n}({\hat{A}}_n(t) - A(t)) = \sqrt{n}\sum _{i=1}^n \int _0^t \frac{J(u) }{Y(u)}(d N_i(u) - d \varLambda _i(u)) + \sqrt{n}\int _0^t (J(u) -1 ) d A(u), \end{aligned}$$

where $d\varLambda _i = Y_i d A$. As the integrand $k_{n} = \tfrac{n J}{Y}$ is bounded by *J* and predictable due to the predictability of *Y*, the first term on the right-hand side of (19) is a square integrable martingale. This martingale refers to $D_{n,k}$, cf. (5). The second term on the right-hand side of (19) is asymptotically negligible as $n \rightarrow \infty$, because $J(t) {\mathop {\longrightarrow }\limits ^{{\mathbb {P}}}} 1$ as $n \rightarrow \infty$, $t\in \mathcal {T}$. Hence, (7) is satisfied. Furthermore, we make the natural assumption that there exists a deterministic function *y*, which is bounded away from zero on $\mathcal {T}$ and such that

20 $$\begin{aligned} \sup _{t\in \mathcal {T}}\big |\frac{Y(t)}{n} - y(t)\big |= o_p(1). \end{aligned}$$

This weak assumption implies Assumption 1. Moreover, we deal with a nonparametric model and as such we have for $t \in \mathcal {T}$, $D k_{n}(t) \equiv 0$. This implies that Assumption 2 is trivially satisfied and that ${{\textbf {B}}}_n \equiv 0$. Additionally, due to the nonparametric model, the assumption (8) is superfluous and we set ${{\textbf {C}}}_n=0$ and ${{\textbf {D}}}_{n,g}(\tau )= 0$. Therefore, also Assumptions 3 and 4 are redundant. In conclusion, for the normalized Nelson–Aalen process $\sqrt{n}({\hat{A}}_n - A)$ given in (19), the asymptotic representation (9) holds with ${{\textbf {B}}}_n {{\textbf {C}}}_n {{\textbf {D}}}_{n,g}(\tau )\equiv 0$, i.e., $\sqrt{n}({\hat{A}}_n - A) = D_{n,k} + o_p(1)$. According to Replacement 1, the wild bootstrap version of the normalized Nelson–Aalen process is

$$\begin{aligned} \sqrt{n}({\hat{A}}^*_n(t) - {\hat{A}}_n(t)) = \sqrt{n} \sum _{i=1}^n \int _0^t \frac{J(u)}{Y(u)} G_i(u) dN_i(u) , \quad t \in \mathcal {T}, \end{aligned}$$

where the term on the right-hand side of the equation above refers to $D_{n,k}^*$, cf. (16). Thus, also (18) holds with ${{\textbf {B}}}^*_n {{\textbf {C}}}^*_n {{\textbf {D}}}^*_{n,g}(\tau )\equiv 0$ and $o_p(1)$ set to zero, i.e., $\sqrt{n}({\hat{A}}^*_n - {\hat{A}}_n) =D_{n,k}^*$. Finally, Theorem 3 can be used to justify the wild bootstrap for the Nelson–Aalen estimator. In particular, the (conditional) distributions of $\sqrt{n}({\hat{A}}_n(t) - A(t))$ and $\sqrt{n}({\hat{A}}^*_n(t) - {\hat{A}}_n(t))$ are asymptotically equivalent.

Furthermore, similar structures hold for more general multivariate Nelson–Aalen estimators beyond simple survival set-ups; here, the time-dependence of the multiplier processes is crucially important (Bluhmki et al. 2019). Indeed, when time-constant multiplier processes are used instead of time-dependent multiplier processes as defined in Sect. 3, the asymptotic distribution would be incorrect. We illustrate this for the resampling Nelson–Aalen estimator $\displaystyle {\hat{A}}_n(t) = \sum \nolimits _{i=1}^n \int _0^t \frac{J(u)dN_i(u)}{Y(u)}$ when $N_i$ may have multiple events per individual, $N_i(\tau ) \in \mathbb {N}$. The Nelson–Aalen estimator has an asymptotic variance of the form $\displaystyle \int _0^t \tfrac{\alpha (u)}{y(u)} du/n$. The resampling version of the normalized Nelson–Aalen estimator is $\displaystyle \sqrt{n} \sum \nolimits _{i=1}^n \int _0^t \frac{ G_i(u)d N_i(u)}{Y(u)}$, where $G_i(u)$ is defined as previously in the present paper. Considered as a martingale, it is easy to see that it exhibits the predictable variation process $\displaystyle n\sum \nolimits _{i=1}^n \int _0^t \frac{d N_i(u)}{Y^2(u)}, t\in \mathcal {T}$, which converges to the desired asymptotic variance function.

However, if each $G_i$ were a time-constant random variable, then the conditional variance of the bootstrapped normalized Nelson–Aalen estimator equals

$$\begin{aligned} V(t)&:= n \sum _{i=1}^n \Bigg (\int _0^t \frac{d N_i(u)}{Y(u)}\Bigg )^2 \\&\quad = n \sum _{i=1}^n \int _0^t \frac{d N_i(u)}{Y^2(u)} + n \sum _{i=1}^n \int _{u \in (0,t]}\int _{v \in (0,t] \setminus \{u\}} \frac{d N_i(u) dN_i(v)}{Y(u) Y(v)} . \end{aligned}$$

Using the approximation $Y(u)/n \approx y(u)$, $dN_i(u) = dM_i(u) + \alpha (u) du$, as well as the uncorrelated martingale increments, the law of large numbers and straightforward algebra result in

$$V(t) {\mathop {\rightarrow }\limits ^{p}}\int _0^t \frac{\alpha (u)}{y(u)} d u + \int _0^t \int _0^t \frac{y(u \vee y)}{y(u) y(v)} \alpha (u) \alpha (v) du dv, \quad \text {as } n \rightarrow \infty .$$

This reveals that the limiting distribution would be corrupted by time-constant multipliers. Additionally, the time-constant multipliers are counterintuitive, as they would not conserve the martingale property.$\square$

### Example 4

*(Weighted logrank test)* The two-sample weighted logrank statistic is

21 $$\begin{aligned} \begin{aligned} T_{n_1,n_2}(w)&= \sqrt{\frac{n_1+ n_2}{n_1 n_2}} \int _0^\infty w({\hat{S}}_n(t-)) \frac{Y^{(1)}(t) Y^{(2)}(t)}{Y(t)} (d{\hat{A}}^{(1)}_{n}(t) - d {\hat{A}}^{(2)}_{n}(t))\\&= \frac{1}{\sqrt{n_1}} \sum _{i=1}^{n_1}\int _0^\infty \sqrt{\frac{n_1+ n_2}{n_2}} w({\hat{S}}_n(t-)) \frac{Y^{(2)}(t) }{Y(t)}dN_i^{(1)}(t) \\&\quad - \frac{1}{\sqrt{n_2}} \sum _{i=1}^{n_2}\int _0^\infty \sqrt{\frac{n_1+ n_2}{n_1}} w({\hat{S}}_n(t-)) \frac{Y^{(1)}(t) }{Y(t)}dN_i^{(2)}(t) , \end{aligned} \end{aligned}$$

where ${\hat{A}}^{(j)}_{n}$ are the Nelson–Aalen estimators, $N^{(j)}_i$, $i=1,\ldots , n$, the counting processes, and $Y^{(j)}$ the at-risk indicators in samples $j=1,2$, $n_1,n_2$ are the sample sizes, $Y=Y^{(1)} + Y^{(2)}$, *w* is a positive weight function, and ${\hat{S}}_n$ is the Kaplan-Meier estimator Kaplan and Meier (1958) in the pooled sample, cf., e.g., Ditzhaus and Friedrich (2020) who conducted weighted logrank tests as permutation tests and Ditzhaus and Pauly (2019) who used the wild bootstrap. Hence, $T_{n_1,n_2}(w)$ is composed of two counting process-based statistics of a form similar to the one given in (1) evaluated at the upper integration bound $\infty$, that is, $T_{n_1,n_2}(w) = \sqrt{n_1} X^{(1)}_{n_1,n_2}(\infty ) + \sqrt{n_2} X^{(2)}_{n_1,n_2}(\infty )$, where the integrand of $X^{(1)}_{n_1,n_2}(\infty )$ equals $k^{(1)}_{n_1,n_2}(t) = { \displaystyle \sqrt{\frac{n_1+n_2}{n_2}} w({\hat{S}}_n(t-)) \frac{Y^{(2)}(t)}{Y(t)}}$ and the integrand of $X^{(2)}_{n_1,n_2}(\infty )$ equals $k^{(2)}_{n_1,n_2}(t) ={ \displaystyle - \sqrt{\frac{n_1+n_2}{n_1}} w({\hat{S}}_n(t-)) \frac{Y^{(1)}(t)}{Y(t)}}$, $t\ge 0$.

Under the null hypothesis of equal hazards, $H_0: A^{(1)} = A^{(2)}$, we have

22 $$\begin{aligned} \begin{aligned}&Y^{(2)}\sum _{i=1}^{n_1}dN_i^{(1)} - Y^{(1)}\sum _{i=1}^{n_2}dN_i^{(2)}\\&\quad = Y^{(2)}\Bigg (\sum _{i=1}^{n_1}dM_i^{(1)}+Y^{(1)}dA^{(1)}\Bigg ) - Y^{(1)}\Bigg (\sum _{i=1}^{n_2}dM_i^{(2)}+Y^{(2)}dA^{(2)}\Bigg ) \\&\quad {\mathop {=}\limits ^{H_0}} Y^{(2)}\sum _{i=1}^{n_1}dM_i^{(1)} - Y^{(1)}\sum _{i=1}^{n_2}dM_i^{(2)}, \end{aligned} \end{aligned}$$

where we have applied the decomposition (3) in the first step of (22), and $M^{(j)}_{i}$, $i=1,\dots , n_j$, are the sample *j*-specific counting process martingales.

Due to (22), the test statistic $T_{n_1,n_2}(w)$ has the following form

under the null hypothesis:

23 $$\begin{aligned} \begin{aligned} T_{n_1,n_2}(w)&{\mathop {=}\limits ^{H_0}} \frac{1}{\sqrt{n_1} } \sum _{i=1}^{ n_1 } \int _0^\infty \sqrt{\frac{n_1 + n_2}{n_2}}w({\hat{S}}_n(t-)) \frac{Y^{(2)}(t)}{Y(t)} d M^{(1)}_{i}(t) \\&\quad - \frac{1}{\sqrt{n_2} } \sum _{i=1}^{ n_2 } \int _0^\infty \sqrt{\frac{n_1 + n_2}{n_1}}w({\hat{S}}_n(t-)) \frac{Y^{(1)}(t)}{Y(t)} d M^{(2)}_{i}(t). \end{aligned} \end{aligned}$$

Under regularity conditions on the weight function and the sample sizes (${ \displaystyle \frac{n_j}{n_1+n_2}\rightarrow \nu _j }$ as $\min (n_1,n_2) \rightarrow \infty$, with $\nu _j \in (0,1)$, $j=1,2$), the stochastic processes $k^{(j)}_{n_1,n_2}$, $j=1,2$, are uniformly bounded on any interval ${\mathcal {T}} = [0,\tau ]$. Clearly, they are also predictable. Thus, under $H_0$, the test statistic can be written as the sum of two square integrable martingales of a form similar to the one given in (5) evaluated at the upper integration bound $\infty$, i.e., $T_{n_1,n_2}(w) {\mathop {=}\limits ^{H_0}} D_{n_1,n_2,k^{(1)}}(\infty ) + D_{n_1,n_2,k^{(2)}}(\infty )$, where the square integrable martingale $D_{n_1,n_2,k^{(1)}}(t)$, $t\ge 0$, relates to the first term on the right-hand side of (23) and the square integrable martingale $D_{n_1,n_2,k^{(2)}}(t)$, $t\ge 0$, relates to the second term on the right-hand side of (23). In order to obtain a similar structure for $T_{n_1,n_2}(w)$ as given in (9), we consider the 2-dimensional vectors ${{\textbf {M}}}_{n_1,n_2}^\top = (\frac{1}{\sqrt{n_1}}\sum _{i=1}^{ n_1 } M_i^{(1)}, \frac{1}{\sqrt{n_2}}\sum _{i=1}^{ n_2 } M_i^{(2)})^\top$ and ${\textbf {k}}_{n_1,n_2}^\top = ({k}^{(1)}_{n_1,n_2},{k}^{(2)}_{n_1,n_2})^\top$, $t\ge 0$. With this notation we get

24 $$\begin{aligned} T_{n_1,n_2}(w) {\mathop {=}\limits ^{H_0}} \int _0^\infty {\textbf {k}}_{n_1,n_2}(t)^\top d{{\textbf {M}}}_{n_1,n_2}(t), \end{aligned}$$

where the right-hand side of (24) is the multidimensional martingale counterpart of the first term on the right-hand side of (9). With (24) we thus obtained a similar structure for $T_{n_1,n_2}(w)$ as in (9) with the second term on the right-hand side of (9) set to zero due to the nonparametric setting. The wild bootstrap version $T^*_{n_1,n_2}(w)$ of $T_{n_1,n_2}(w)$ under $H_0$ is obtained by applying Replacement 1 to (24):

25 $$\begin{aligned} T^*_{n_1,n_2}(w) {\mathop {=}\limits ^{H_0}} \int _0^\infty {\textbf {k}}^*_{n_1,n_2}(t)^\top d{{\textbf {M}}}^*_{n_1,n_2}(t), \end{aligned}$$

where ${{\textbf {M}}}^{*\top }_{n_1,n_2} = (\frac{1}{\sqrt{n_1}}\sum _{i=1}^{ n_1 } G^{(1)}_i N_i^{(1)}, \frac{1}{\sqrt{n_2}}\sum _{i=1}^{ n_2 } G^{(2)}_i N_i^{(2)})^\top$ is the wild bootstrap counterpart of ${{\textbf {M}}}_{n_1,n_2}$, and ${\textbf {k}}^{*\top }_{n_1,n_2} = ({k}^{*(1)}_{n_1,n_2},{k}^{*(2)}_{n_1,n_2})^\top$ with

$${k}^{*(j)}_{n_1,n_2}(t) = (-1)^{j+1} \sqrt{\frac{n_1 + n_2}{n_{3-j}}} w({\hat{S}}_n^*(t-)) \frac{Y^{(3-j)}(t)}{Y(t)}, \quad t\ge 0, j=1,2,$$

is the wild bootstrap counterpart of ${\textbf {k}}_{n_1,n_2}$. Here, the multiplier processes $G^{(1)}_1,\ldots ,G^{(1)}_{n_1},$ $G^{(2)}_1,\ldots , G^{(2)}_{n_2}$ are pairwise independent and identically distributed. Note that this definition of $T^*_{n_1,n_2}(w)$ deviates slightly from the corresponding definition given in Ditzhaus and Pauly (2019) as it contains the wild bootstrap counterpart ${\hat{S}}_n^*$ of the pooled Kaplan-Meier estimator ${\hat{S}}_n$. In the related paper (Dietrich et al. 2023), an idea of how such a resampling version may be constructed based on a functional relationship between the estimator of interest and Nelson–Aalen estimators is given; this is exemplified by means of cumulative incidence functions in semiparametric models. With (25) we thus obtained a similar structure for $T^*_{n_1,n_2}(w)$ as stated in (18) with ${{\textbf {B}}}^*_n {{\textbf {C}}}^*_n {{\textbf {D}}}^*_{n,g}(\tau )\equiv 0$ due to the nonparametric setting and $o_p(1)$ set to zero. It is left to show that a result as stated in Theorem 3 holds for $T_{n_1,n_2}(w)$ and $T^*_{n_1,n_2}(w)$ under the null hypothesis. For this, one may first argue with respect to any finite upper bound of integration $\tau$. With one additional argument, the remaining integral from $\tau$ to $\infty$ can be shown to be asymptotically negligible for $n \rightarrow \infty$ followed by $\tau \rightarrow \infty$; use for instance Theorem 3.2 in Billingsley (1999). In this way, one obtains a justification of the wild bootstrap for the weighted logrank test within a multidimensional martingale framework which can be seen as an extension of the setting presented in this paper. $\square$

## Discussion

We have proposed and validated a widely applicable wild bootstrap procedure for general nonparametric and (semi-)parametric counting process-based statistics. We gave a step by step description of how to construct the wild bootstrap counterpart of the statistic. In particular, it is crucial to match each individual with one multiplier process. In order to justify the validity of the wild bootstrap, we have studied the (conditional) asymptotic distributions of the statistic of interest and of the wild bootstrap counterpart which turned out to coincide. We have found the wild bootstrapped martingales to be martingales as well. Thus, in the corresponding proof, we made use of a carefully chosen variant of Rebolledo’s martingale central limit theorem. We illustrated the method for several main models in survival analysis.

The model assumptions in this paper are rather weak: they are satisfied under very natural regularity conditions; cf. Examples 1g, 3, and 4. However, Assumption 1 (c) is, for example, not satisfied in shared frailty models, because in these models it is assumed that common unobserved variables influence the intensity processes of multiple individuals. In this regard, the assumption of identically distributed counting processes could also be too strong for particular experimental designs, e.g., if one does sample in a stratified manner instead of randomly. However, the wild bootstrap is known to yield proper estimates for different residual distributions and we expect that, if all regularity conditions of the present paper are adjusted accordingly, the main results will also hold in the non-identically distributed regime.

For the construction of the wild bootstrap counterpart of a given counting process-based statistic we have chosen the nonparametric estimator $G_i dN_i$ for the martingale increment $dM_i$, cf. Replacement 1 (a). This choice guarantees a more general applicability of the proposed wild bootstrap resampling procedure, because no specifications on the form of the cumulative hazard function have to be made. In contrast, Spiekerman and Lin (1998) proposed a semiparametric approach by choosing ${G_i d {\hat{M}}_i =} G_i [dN_i - d{\hat{\varLambda }}_i(\cdot ,\varvec{\hat{\beta}}_n)]$ as the replacement for the martingale increment. Under this semiparametric estimator the information encoded in the parameter $\varvec{\beta }$ is incorporated in the wild bootstrap estimators, which could potentially lead to more accurate results. However, their approach is not as widely applicable as the nonparametric one that we decided to employ. Moreover, in the context of Cox models, Dobler et al. (2019) revealed by means of a substantial simulation study that the difference between the results of the two methods is not significant.

Although the assumed martingale structure for $\varvec{\hat{\beta}}_n$ is satisfied in many models and common estimation strategies, it could be too restrictive for certain applications. For instance, if not the intensity process $\lambda _i(t)dt = {\mathbb {E}}(d N_i(t) | \mathcal {F}_1(t-))$ but only the rate process $\gamma _i(t)dt = {\mathbb {E}}(d N_i(t) | N_i(t-), Y_i(t), \varvec{Z}_i(t))$ is modeled, then ${\tilde{M}}_i(t) = N_i(t) - \int _0^t \gamma _i(u) du, t\in {\mathcal {T}}$, still defines a zero-mean process albeit not necessarily a martingale; cf. Scheike and Zhang (2003) for a similar observation in the context of Cox-Aalen models. The authors of the above mentioned paper proposed to use $G_i d {\hat{M}}_i(t)$ as resampling counterparts of $d{\tilde{M}}_i(t)$, where $d{\hat{M}}_i(t) =d N_i(t) - {\hat{\gamma }}_i(t) dt$ involves the estimated rate function. Thus, whenever $\varvec{\hat{\beta}}_n$ does not have a martingale representation, but still exhibits an asymptotically linear structure in terms of ${\tilde{M}}_i$, the approach considered in the present paper motivates as a first resampling attempt to incorporate time-dependent multipliers, resulting in $G_i(t) d {\hat{M}}_i(t)$ instead of $G_i d {\hat{M}}_i(t)$. It remains to be investigated which of the two approaches is more suitable as a general resampling procedure for rate models. In any case, it will be essential to ensure the correct correlation between the resampled estimators of the parametric and the nonparametric model components.

Finally, we would like to compare the wild bootstrap to Efron’s bootstrap, i.e., drawing *n* times with replacement from the individual data points. A clear advantage of the wild bootstrap over Efron’s bootstrap is the computational efficiency: as the wild bootstrap exploits the asymptotically linear structures of estimators, it also only requires linear operations for the computation. In contrast, Efron’s bootstrap demands a potentially costly re-computation of all (potentially nonlinear) estimators based on the bootstrap samples. However, this feature of the wild bootstrap could also be considered a drawback because the asymptotically linear structure (and martingale representation) of each estimator has to be derived. Moreover, Efron’s bootstrap is widely applicable for i.i.d. data and, according to some heuristic calculations we made, even seems to correctly resample the general Nelson–Aalen estimator for survival data with possibly multiple events per individual. On the other hand, the wild bootstrap has originally been proposed for applications to heteroscedastic data (Chien-Fu 1986) and, in this sense, it is applicable even beyond the i.i.d. setting. Furthermore, it allows for a great flexibility in the choice of multiplier distributions which could result in a better match of the martingale distribution.

In conclusion, the wild bootstrap procedure as presented in this paper is applicable to a wide range of models and simple to implement. By means of this method, one may easily approximate the unknown distribution of a counting process-based statistic around the target quantity. In a connected paper (Dietrich et al. 2023), the results of the present paper are extended to a functional of counting process-based estimators via the functional $\delta$-method. In particular, a weak convergence result for the cumulative incidence function is derived in the context of the Fine–Gray model for censoring-complete data and this result is used to construct time-simultaneous confidence bands for that function. Generalizing the theory of the current paper to functionals of a counting process-based statistic via the functional $\delta$-method constitutes an interesting extension for future research.

## Supporting Information

Web Appendix A: Supporting Information contains all proofs, additional lemmas, and an additional corollary. It is referenced in Sects. 2 and 3 and is available online.

## Supplementary Information

Below is the link to the electronic supplementary material.

Supplementary file 1 (pdf 430 KB)
