# Supplementary material for: Wild bootstrap for counting process-based statistics: a martingale theory-based approach
Source: Lifetime Data Anal. 2025 Jul 28;31(3):631–57. doi: 10.1007/s10985-025-09659-w (PMC12317882; doi:10.1007/s10985-025-09659-w)
Supplement: Supplementary file 1 — (pdf 430 KB) [file 10985_2025_9659_MOESM1_ESM.pdf]

Lifetime Data Analysis manuscript No.
(will be inserted by the editor)

Supporting Information for Wild Bootstrap for Counting Process-Based Statistics: A Martingale Theory-Based Approach

Marina T. Dietrich · Dennis Dobler · Mathisca C. M. de Gunst

Received: 9 September 2024 / Accepted: 22 May 2025

Abstract This Supporting Information contains all proofs of the main manuscript, additional lemmas and an additional corollary.

Keywords Counting processes · martingale theory · resampling · statistical inference · survival analysis · wild bootstrap

Marina T. Dietrich (ORCID: 0009-0008-6882-2568)
Department of Mathematics, Vrije Universiteit Amsterdam, 1081 HV Amsterdam, The Netherlands
E-mail: marina.dietrich@uni-a.de
Present address:
Department of Mathematics, University Augsburg, 86159 Augsburg, Germany

Dennis Dobler (ORCID: 0000-0002-9040-0854)
Department of Mathematics, Vrije Universiteit Amsterdam, 1081 HV Amsterdam, The Netherlands
Present address:
Department of Statistics, TU Dortmund University, Research Center Trustworthy Data Science and Security, University Alliance Metropolis Ruhr, 44227 Dortmund, Germany

Mathisca C. M. de Gunst (ORCID: 0000-0002-8213-6467)
Department of Mathematics, Vrije Universiteit Amsterdam, 1081 HV Amsterdam, The Netherlands

Web Appendix A: Proofs and Lemmas

For the proofs we introduce some additional notation: we write $\|\cdot\|_\infty$ for the maximum norm of a vector $\boldsymbol{v} \in \mathbb{R}^p$ or a matrix $\mathbf{G} \in \mathbb{R}^{p\times p}$, which denotes the largest element in absolute value of $\boldsymbol{v}$ and $\mathbf{G}$, respectively. Moreover, $\mathcal{C}[0,\tau]^m$ denotes the set of all continuous functions with values from $[0,\tau]$ to $\mathbb{R}^m$ for any $m \in \mathbb{N}$.

A.1 Proofs and Lemmas of Section 2

Proof of Lemma 1. To find the asymptotic representation (9), we use the Doob-Meyer decomposition (3) and a Taylor expansion around $\boldsymbol{\beta}_0$:

$$\begin{aligned}
&\sqrt{n}(\mathbf{X}_n(t) - \mathbf{X}(t))\\
&= \sqrt{n}\Big(\frac{1}{n}\sum_{i=1}^n \int_0^t \big[\mathbf{k}_{n,i}(u,\hat{\boldsymbol{\beta}}_n) - \mathbf{k}_{n,i}(u,\boldsymbol{\beta}_0) + \mathbf{k}_{n,i}(u,\boldsymbol{\beta}_0)\big] dN_i(u) - \mathbf{X}(t)\Big)\\
&= \sqrt{n}\Big(\frac{1}{n}\sum_{i=1}^n \int_0^t \mathbf{k}_{n,i}(u,\boldsymbol{\beta}_0) dM_i(u) + \frac{1}{n}\sum_{i=1}^n \int_0^t \mathbf{k}_{n,i}(u,\boldsymbol{\beta}_0) dA_i(u,\beta_0) - \mathbf{X}(t)\\
&\qquad + \big(\frac{1}{n}\sum_{i=1}^n \int_0^t \mathrm{D}\mathbf{k}_{n,i}(u,\boldsymbol{\beta}_0) dN_i(u)\big)(\hat{\boldsymbol{\beta}}_n - \boldsymbol{\beta}_0) + o_p(\hat{\boldsymbol{\beta}}_n - \boldsymbol{\beta}_0)\Big),
\end{aligned} \tag{38}$$

where $\mathrm{D}\mathbf{k}_{n,i}$ is as defined below (6). Combining (2), (7), and (38) reveals that

$$\begin{aligned}
\sqrt{n}(\mathbf{X}_n(t) - \mathbf{X}(t)) &= \frac{1}{\sqrt{n}}\sum_{i=1}^n \int_0^t \mathbf{k}_{n,i}(u,\boldsymbol{\beta}_0) dM_i(u)\\
&\quad + \big(\frac{1}{n}\sum_{i=1}^n \int_0^t \mathrm{D}\mathbf{k}_{n,i}(u,\boldsymbol{\beta}_0) dN_i(u)\big)\sqrt{n}(\hat{\boldsymbol{\beta}}_n - \boldsymbol{\beta}_0) + o_p(1),
\end{aligned} \tag{39}$$

where the $(p \times q)$-dimensional counting process integral in (39) refers to $\mathbf{B}_n(t)$, $t \in [0,\tau]$, defined in (6). Finally, (5), (6), (8), and (39) combined yield the desired asymptotic representation (9).

Lemma 4 *Under the general model assumptions, i.e., the i.i.d. structure, the finiteness of* $\mathbb{E}(A_i(\tau,\boldsymbol{\beta}_0))$, *and the assumed uniform boundedness of the integrands* $h_{n,i}(s,\boldsymbol{\beta}_0), i = 1,\dots,n; s \in (0,\tau]$, *the Lindeberg condition of Theorem 1 is satisfied.*

Proof of Lemma 4. A two-fold application of Hölder's inequality with $p, q > 1$ such that $1/p + 1/q = 1$ yields

$$\mathbb{E}(\sigma^\epsilon[D_{n,h}](t)) = \mathbb{E}\Big(\sum_{s\le t}(\Delta D_{n,h}(s))^2 \mathbb{1}\{|\Delta D_{n,h}(s)| > \epsilon\}\Big)$$

$$
\begin{aligned}
&= \sum_{i=1}^{n} \mathbb{E}\Big(\sum_{s \le t} \frac{h^2_{n,i}(s,\boldsymbol{\beta}_0)}{n} \Delta N_i(s) \mathbb{1}\{|h_{n,i}(s,\boldsymbol{\beta}_0)| > \sqrt{n}\epsilon\} \Big) \\
&\le \sum_{i=1}^{n} \mathbb{E}\Big(\Big(\sum_{s \le t} \frac{|h_{n,i}|^{2p}(s,\boldsymbol{\beta}_0)}{n^p} \Delta N_i(s) \Big)^{1/p} \\
&\quad \cdot \Big(\sum_{s \le t} \mathbb{1}\{|h_{n,i}(s,\boldsymbol{\beta}_0)| > \sqrt{n}\epsilon\} \Delta N_i(s) \Big)^{1/q} \Big) \\
&\le \sum_{i=1}^{n} \mathbb{E}\Big(\int_0^t \frac{|h_{n,i}|^{2p}(s,\boldsymbol{\beta}_0)}{n^p} dN_i(s) \Big)^{1/p} \\
&\quad \cdot \mathbb{E}\Big(\int_0^t \mathbb{1}\{|h_{n,i}(s,\boldsymbol{\beta}_0)| > \sqrt{n}\epsilon\} dN_i(s) \Big)^{1/q} \\
&= \mathbb{E}\Big(\int_0^t |h_{n,1}|^{2p}(s,\boldsymbol{\beta}_0) dN_1(s) \Big)^{1/p} \\
&\quad \cdot \mathbb{E}\Big(\int_0^t \mathbb{1}\{|h_{n,1}(s,\boldsymbol{\beta}_0)| > \sqrt{n}\epsilon\} dN_1(s) \Big)^{1/q},
\end{aligned}
$$

where for the last equality we used the i.i.d. data structure. Due to the martingale property and Fubini's theorem, the last terms simplify to

$$
\begin{aligned}
&\Big(\int_0^t \mathbb{E}(|h_{n,1}|^{2p}(s,\boldsymbol{\beta}_0) Y_1(s) \alpha_1(s,\boldsymbol{\beta}_0)) ds \Big)^{1/p} \\
&\cdot \Big(\int_0^t \mathbb{E}(\mathbb{1}\{|h_{n,1}(s,\boldsymbol{\beta}_0)| > \sqrt{n}\epsilon\} Y_1(s) \alpha_1(s,\boldsymbol{\beta}_0)) ds \Big)^{1/q}.
\end{aligned}
$$

Because $h_{n,1}$ is uniformly bounded, there exists a constant $C > 0$ such that the previous expression is bounded above by

$$
\mathbb{1}\{n < C^2/\epsilon^2\} \cdot C^2 \cdot \Big(\int_0^t \mathbb{E}(Y_1(s)) \alpha_1(s,\boldsymbol{\beta}_0)) ds \Big)^{1/p} \Big(\int_0^t \mathbb{E}(Y_1(s) \alpha_1(s,\boldsymbol{\beta}_0)) ds \Big)^{1/q}.
$$

Due to the assumed finiteness of the expectation of the cumulative intensity process, $\mathbb{E}(\Lambda_i(\tau)) < \infty$, the expectation in the previous display is finite and equal to zero for sufficiently large n. □

The following lemma contains a central limit theorem for $\mathbf{D}_{n,h}$ in the space of càdlàg functions:

Lemma 5 *If Assumption 1 holds, then*

$$
\boldsymbol{D}_{n,h} \xrightarrow{\mathcal{L}} \boldsymbol{D}_{\tilde{h}}, \quad \text{in } (D(\mathcal{T}))^{p+b}, \text{ as } n \to \infty,
$$

where $\boldsymbol{D}_{\tilde{h}} = (\boldsymbol{D}_{\tilde{k}}^\top, \boldsymbol{D}_{\tilde{g}}^\top)^\top$ *is a continuous zero-mean Gaussian* $(p+b)$*-dimensional vector martingale with* $\langle \boldsymbol{D}_{\tilde{h}} \rangle(t) = \boldsymbol{V}_{\tilde{h}}(t) = \int_0^t \mathbb{E}(\tilde{\boldsymbol{h}}_1(u,\boldsymbol{\beta}_0)^{\otimes 2} \lambda_1(u,\boldsymbol{\beta}_0)) du$, $t \in \mathcal{T}$.

In particular, $\boldsymbol{V}_{\tilde{h}} = \begin{pmatrix} \boldsymbol{V}_{\tilde{k}} & \boldsymbol{V}_{\tilde{k},\tilde{g}} \\ \boldsymbol{V}_{\tilde{g},\tilde{k}} & \boldsymbol{V}_{\tilde{g}} \end{pmatrix}$*, with variances and covariances respectively given by*

$$\boldsymbol{V}_{\tilde{k}}(t) = \langle \boldsymbol{D}_{\tilde{k}} \rangle(t) = \int_0^t \mathbb{E}(\tilde{\boldsymbol{k}}_1(u,\boldsymbol{\beta}_0)^{\otimes 2}\lambda_1(u,\boldsymbol{\beta}_0))du, \quad t \in \mathcal{T},$$
$$\boldsymbol{V}_{\tilde{g}}(t) = \langle \boldsymbol{D}_{\tilde{g}} \rangle(t) = \int_0^t \mathbb{E}(\tilde{\boldsymbol{g}}_1(u,\boldsymbol{\beta}_0)^{\otimes 2}\lambda_1(u,\boldsymbol{\beta}_0))du, \quad t \in \mathcal{T},$$
$$\boldsymbol{V}_{\tilde{k},\tilde{g}}(t) = \boldsymbol{V}_{\tilde{g},\tilde{k}}(t)^\top = \langle \boldsymbol{D}_{\tilde{k}}, \boldsymbol{D}_{\tilde{g}} \rangle(t) = \int_0^t \mathbb{E}(\tilde{\boldsymbol{k}}_1(u,\boldsymbol{\beta}_0)\tilde{\boldsymbol{g}}_1(u,\boldsymbol{\beta}_0)^\top\lambda_1(u,\boldsymbol{\beta}_0))du, \quad t \in \mathcal{T}.$$

We note that $\mathbf{V}_{\tilde{h}}(t)$, $t \in \mathcal{T}$, in Lemma 5 is by construction a continuous, deterministic and positive semidefinite matrix-valued function with $\mathbf{V}_{\tilde{h}}(0) = 0$.

Proof of Lemma 5.
Since the integrand $\mathbf{h}_{n,i}(t,\boldsymbol{\beta}_0) = (\mathbf{k}_{n,i}(t,\boldsymbol{\beta}_0)^\top, \mathbf{g}_{n,i}(t,\boldsymbol{\beta}_0)^\top)^\top$ is a locally bounded and predictable stochastic process, $\mathbf{D}_{n,h}$ is a local square integrable counting process martingale. Thus, we may apply Rebolledo's martingale central limit theorem (Theorem 1). In view of Lemma 4 above, applied to each dimension of the multivariate martingale, it remains to prove the convergence of the predictable (or optional) covariation process. Then the desired result follows from the univariate martingale central limit theorem (Theorem 1) by means of the Cramér-Wold device.

Let us consider the predictable covariation process $\langle \mathbf{D}_{n,h} \rangle(t)$, $t \in \mathcal{T}$. According to Proposition II.4.1 of Andersen et al. [1993], we have

$$\begin{aligned}\langle \mathbf{D}_{n,h} \rangle(t) &= \frac{1}{n}\sum_{i=1}^n \int_0^t \mathbf{h}_{n,i}(u,\boldsymbol{\beta}_0)^{\otimes 2} d\Lambda_i(u,\boldsymbol{\beta}_0) \\ &= \frac{1}{n}\sum_{i=1}^n \int_0^t [\mathbf{h}_{n,i}(u,\boldsymbol{\beta}_0)^{\otimes 2} - \tilde{\mathbf{h}}_i(u,\boldsymbol{\beta}_0)^{\otimes 2}] d\Lambda_i(u,\boldsymbol{\beta}_0) \\ &\quad + \frac{1}{n}\sum_{i=1}^n \int_0^t \tilde{\mathbf{h}}_i(u,\boldsymbol{\beta}_0)^{\otimes 2} d\Lambda_i(u,\boldsymbol{\beta}_0).\end{aligned} \tag{40}$$

We start with focusing on the first term of the second step of (40). We want to show that

$$\frac{1}{n}\sum_{i=1}^n \int_0^t [\mathbf{h}_{n,i}(u,\boldsymbol{\beta}_0)^{\otimes 2} - \tilde{\mathbf{h}}_i(u,\boldsymbol{\beta}_0)^{\otimes 2}] d\Lambda_i(u,\boldsymbol{\beta}_0) = o_p(1), \text{ for all } t \in \mathcal{T}, \text{ as } n \to \infty. \tag{41}$$

For this it suffices to bound its largest component:

$$\begin{aligned}
&\frac{1}{n}\sum_{i=1}^{n}\int_0^t \|\mathbf{h}_{n,i}(u,\boldsymbol{\beta}_0)^{\otimes 2} - \tilde{\mathbf{h}}_i(u,\boldsymbol{\beta}_0)^{\otimes 2}\|_\infty d\Lambda_i(u,\boldsymbol{\beta}_0)\\
&\le \sup_{i\in\{1,\dots,n\},t\in\mathcal{T}} \|\mathbf{h}_{n,i}(t,\boldsymbol{\beta}_0)^{\otimes 2} - \tilde{\mathbf{h}}_i(t,\boldsymbol{\beta}_0)^{\otimes 2}\|_\infty \frac{1}{n}\sum_{i=1}^{n}\Lambda_i(\tau,\boldsymbol{\beta}_0)\\
&\le \Big(\sup_{i\in\{1,\dots,n\},t\in\mathcal{T}} \|(\mathbf{h}_{n,i}(t,\boldsymbol{\beta}_0) - \tilde{\mathbf{h}}_i(t,\boldsymbol{\beta}_0))\mathbf{h}_{n,i}(t,\boldsymbol{\beta}_0)^\top\|_\infty\\
&\qquad + \sup_{i\in\{1,\dots,n\},t\in\mathcal{T}} \|\tilde{\mathbf{h}}_i(t,\boldsymbol{\beta}_0)(\mathbf{h}_{n,i}(t,\boldsymbol{\beta}_0) - \tilde{\mathbf{h}}_i(t,\boldsymbol{\beta}_0))^\top\|_\infty\Big)\frac{1}{n}\sum_{i=1}^{n}\Lambda_i(\tau,\boldsymbol{\beta}_0)
\end{aligned} \tag{42}$$

where the last step is due to the triangle inequality and $\boldsymbol{a}^{\otimes 2} - \boldsymbol{b}^{\otimes 2} = (\boldsymbol{a} - \boldsymbol{b})\boldsymbol{a}^\top + \boldsymbol{b}(\boldsymbol{a}-\boldsymbol{b})^\top$ for two vectors $\boldsymbol{a}, \boldsymbol{b}$. Both terms in brackets converge to zero in probability, as $n \to \infty$, according to Assumption 1 (1), (2), and since $\mathbf{h}_{n,i}(t,\boldsymbol{\beta}_0)$ is locally bounded for $i = 1,\dots,n$. Note that Assumption 1 (1) holds for any consistent estimator $\check{\boldsymbol{\beta}}_n$, in particular for $\boldsymbol{\beta}_0$ itself. From Assumption 1 (3) in combination with the integrability of the cumulative intensities and the law of large numbers, we get $\frac{1}{n}\sum_{i=1}^n \Lambda_i(\tau,\boldsymbol{\beta}_0) \stackrel{\mathbb{P}}{\longrightarrow} \mathbb{E}(\Lambda_1(\tau,\boldsymbol{\beta}_0))$, as $n \to \infty$. Hence, the whole expression converges to zero in probability, as $n \to \infty$, and we conclude that (41) holds.

The subsequent considerations relate to the second term of the second step of (40). According to Assumption 1 (2) it holds that $\sup_{t\in\mathcal{T}}\|\tilde{\mathbf{h}}_1(t,\boldsymbol{\beta}_0)\|_\infty$ is bounded. Moreover, we have $\mathbb{E}(\Lambda_1(\tau,\boldsymbol{\beta}_0)) < \infty$ by assumption. These two statements combined yield for all $t \in \mathcal{T}$,

$$\mathbb{E}\Big(\int_0^t \|\tilde{\mathbf{h}}_1(u,\boldsymbol{\beta}_0)^{\otimes 2}\|_\infty \, d\Lambda_1(u,\boldsymbol{\beta}_0)\Big) \le \mathbb{E}\Big(\sup_{t\in\mathcal{T}}\|\tilde{\mathbf{h}}_1(t,\boldsymbol{\beta}_0)^{\otimes 2}\|_\infty \Lambda_1(t,\boldsymbol{\beta}_0)\Big) < \infty. \tag{43}$$

On the basis of (43) and Assumption 1 (3), we make use of the law of large numbers and get for the second term of the second step of (40)

$$\frac{1}{n}\sum_{i=1}^{n}\int_0^t \tilde{\mathbf{h}}_i(u,\boldsymbol{\beta}_0)^{\otimes 2} d\Lambda_i(u,\boldsymbol{\beta}_0) \stackrel{\mathbb{P}}{\longrightarrow} \mathbb{E}(\int_0^t \tilde{\mathbf{h}}_1(u,\boldsymbol{\beta}_0)^{\otimes 2} d\Lambda_1(u,\boldsymbol{\beta}_0)), \quad n \to \infty,$$

for any fixed $t \in \mathcal{T}$. Note that the integrability of the intensity process $\lambda_1(t,\boldsymbol{\beta}_0)$ follows from the integrability of the cumulative intensity process $\Lambda_1(t,\boldsymbol{\beta}_0)$. Thus, due to the integrability of the cumulative intensities and Assumption 1 (2), we can make use of Fubini's theorem, due to which we can exchange the order of integration. Thus, we have

$$\frac{1}{n}\sum_{i=1}^{n}\int_0^t \tilde{\mathbf{h}}_i(u,\boldsymbol{\beta}_0)^{\otimes 2} d\Lambda_i(u,\boldsymbol{\beta}_0) \stackrel{\mathbb{P}}{\longrightarrow} \int_0^t \mathbb{E}(\tilde{\mathbf{h}}_1(u,\boldsymbol{\beta}_0)^{\otimes 2}\lambda_1(u,\boldsymbol{\beta}_0))du, \tag{44}$$

for all $t \in \mathcal{T}$, as $n \to \infty$. Finally, combining (40) with (41) and (44) yields

$$\langle \mathbf{D}_{n,h} \rangle (t) \xrightarrow{\mathbb{P}} \int_0^t \mathbb{E}(\tilde{\mathbf{h}}_1(u, \boldsymbol{\beta}_0)^{\otimes 2} \lambda_1(u, \boldsymbol{\beta}_0)) du = \mathbf{V}_{\tilde{h}}(t), \text{ for all } t \in \mathcal{T}, \text{ as } n \to \infty.$$

When taking into consideration that we have $\tilde{\mathbf{h}} = (\tilde{\mathbf{k}}, \tilde{\mathbf{g}})$, we can write the covariance matrix in block form

$$\mathbf{V}_{\tilde{h}} = \mathbf{V}_{(\tilde{k},\tilde{g})} = \begin{pmatrix} \mathbf{V}_{\tilde{k}} & \mathbf{V}_{\tilde{k},\tilde{g}} \\ \mathbf{V}_{\tilde{g},\tilde{k}} & \mathbf{V}_{\tilde{g}} \end{pmatrix},$$

where for $t \in \mathcal{T}$,

$$\mathbf{V}_{\tilde{k}}(t) = \langle \mathbf{D}_{\tilde{k}} \rangle (t) = \int_0^t \mathbb{E}(\tilde{\mathbf{k}}_1(u, \boldsymbol{\beta}_0)^{\otimes 2} \lambda_1(u, \boldsymbol{\beta}_0)) du,$$

$$\mathbf{V}_{\tilde{g}}(t) = \langle \mathbf{D}_{\tilde{g}} \rangle (t) = \int_0^t \mathbb{E}(\tilde{\mathbf{g}}_1(u, \boldsymbol{\beta}_0)^{\otimes 2} \lambda_1(u, \boldsymbol{\beta}_0)) du,$$

$$\mathbf{V}_{\tilde{k},\tilde{g}}(t) = \mathbf{V}_{\tilde{g},\tilde{k}}(t) = \langle \mathbf{D}_{\tilde{k}}, \mathbf{D}_{\tilde{g}} \rangle (t) = \int_0^t \mathbb{E}(\tilde{\mathbf{k}}_1(u, \boldsymbol{\beta}_0) \cdot \tilde{\mathbf{g}}_1(u, \boldsymbol{\beta}_0)^\top \lambda_1(u, \boldsymbol{\beta}_0)) du.$$

□

The next lemma describes the limiting behaviour of $\mathbf{B}_n$.

Lemma 6 *If Assumption 2 holds, then* $\sup_{t \in \mathcal{T}} \| \boldsymbol{B}_n(t) - \boldsymbol{B}(t) \| \xrightarrow{\mathbb{P}} 0$, *as* $n \to \infty$, *where* $\boldsymbol{B} : \mathcal{T} \to \mathbb{R}^{p \times q}, t \mapsto \int_0^t \mathbb{E}(\tilde{\boldsymbol{K}}_1(u, \boldsymbol{\beta}_0) \lambda_1(u, \boldsymbol{\beta}_0)) du$ *is continuous and deterministic.*

Proof of Lemma 6.
We wish to show that

$$\sup_{t \in \mathcal{T}} \| \mathbf{B}_n(t) - \mathbf{B}(t) \| \xrightarrow{\mathbb{P}} 0, \text{ as } n \to \infty,$$

where $\mathbf{B}_n(t) = \frac{1}{n} \sum_{i=1}^n \int_0^t \mathrm{D}\mathbf{k}_{n,i}(u, \boldsymbol{\beta}_0) dN_i(u)$ and $\mathbf{B}(t) = \int_0^t \mathbb{E}(\tilde{\mathbf{K}}_1(u, \boldsymbol{\beta}_0) \lambda_1(u, \boldsymbol{\beta}_0)) du$, $t \in \mathcal{T}$. For this we point out that the compensator of $\frac{1}{n} \sum_{i=1}^n N_i(\tau)$ is equal to $\frac{1}{n} \sum_{i=1}^n \Lambda_i(\tau, \boldsymbol{\beta}_0)$. From the integrability of the cumulative intensities, Assumption 2 (3), and the law of large numbers, we can conclude that $\frac{1}{n} \sum_{i=1}^n \Lambda_i(\tau, \boldsymbol{\beta}_0) = O_p(1)$. Thus, we get from Lenglart's inequality that

$\frac{1}{n}\sum_{i=1}^{n} N_i(\tau) = O_p(1)$. Combining this argument with Assumption 2 (1) yields

$$\begin{aligned}
&\sup_{t\in\mathcal{T}} \|\mathbf{B}_n(t) - \mathbf{B}(t)\| \\
&\leq \sup_{t\in\mathcal{T}} \Big\| \frac{1}{n}\sum_{i=1}^{n}\int_0^t [\mathrm{D}\mathbf{k}_{n,i}(u,\boldsymbol{\beta}_0) - \tilde{\mathbf{K}}_i(u,\boldsymbol{\beta}_0)]dN_i(u)\Big\| \\
&\quad + \sup_{t\in\mathcal{T}} \Big\| \frac{1}{n}\sum_{i=1}^{n}\int_0^t \tilde{\mathbf{K}}_i(u,\boldsymbol{\beta}_0)dN_i(u) - \int_0^t \mathbb{E}(\tilde{\mathbf{K}}_1(u,\boldsymbol{\beta}_0)\lambda_1(u,\boldsymbol{\beta}_0))du\Big\| \\
&\leq \sup_{t\in\mathcal{T}} \Big\| \frac{1}{n}\sum_{i=1}^{n}\int_0^t \tilde{\mathbf{K}}_i(u,\boldsymbol{\beta}_0)dM_i(u)\Big\| \\
&\quad + \sup_{t\in\mathcal{T}} \Big\| \frac{1}{n}\sum_{i=1}^{n}\int_0^t \tilde{\mathbf{K}}_i(u,\boldsymbol{\beta}_0)d\Lambda_i(u,\boldsymbol{\beta}_0) - \int_0^t \mathbb{E}(\tilde{\mathbf{K}}_1(u,\boldsymbol{\beta}_0)\lambda_1(u,\boldsymbol{\beta}_0))du\Big\| + o_p(1),
\end{aligned} \tag{45}$$

where in the last step the Doob-Meyer decomposition (3) has been applied. With Assumption 2 (2) and Proposition II.4.1 of Andersen et al. [1993] it follows that the integral $\frac{1}{n}\sum_{i=1}^{n}\int_0^t \tilde{\mathbf{K}}_i(u,\boldsymbol{\beta}_0)dM_i(u)$ is a local square integrable martingale. The elements of the corresponding predictable covariation process at τ can be bounded from above by

$$\frac{1}{n^2}\sum_{i=1}^{n}\int_0^\tau \|\tilde{\mathbf{K}}_i(u,\boldsymbol{\beta}_0)\|_\infty^2 d\Lambda_i(u,\boldsymbol{\beta}_0).$$

According to Assumption 2 (2), $\sup_{i\in\{1,\dots,n\},t\in\mathcal{T}}\|\tilde{\mathbf{K}}_i(t,\boldsymbol{\beta}_0)\|_\infty^2$ is bounded for $i \in \mathbb{N}$, and, as stated above, it holds $\frac{1}{n}\sum_{i=1}^{n}\Lambda_i(\tau,\boldsymbol{\beta}_0) = O_p(1)$. Hence, the considered predictable covariation process and further, according to Lenglart's inequality, the first term of the second step on the right-hand side of (45) converges to zero in probability, as $n \to \infty$. It is only left to show that

$$\sup_{t\in\mathcal{T}} \Big\| \frac{1}{n}\sum_{i=1}^{n}\int_0^t \tilde{\mathbf{K}}_i(u,\boldsymbol{\beta}_0)d\Lambda_i(u,\boldsymbol{\beta}_0) - \int_0^t \mathbb{E}(\tilde{\mathbf{K}}_1(u,\boldsymbol{\beta}_0)\lambda_1(u,\boldsymbol{\beta}_0))du\Big\| = o_p(1), \tag{46}$$

as $n \to \infty$. According to the integrability of the cumulative intensities and Assumption 2 (2) it follows that $\mathbb{E}(\int_0^t \|\tilde{\mathbf{K}}_1(u,\boldsymbol{\beta}_0)\|_\infty \lambda_1(u,\boldsymbol{\beta}_0)du) < \infty$. From this argument in combination with Assumption 2 (3) and the law of large numbers, we have that $\frac{1}{n}\sum_{i=1}^{n}\int_0^t \tilde{\mathbf{K}}_i(u,\boldsymbol{\beta}_0)\lambda_i(u,\boldsymbol{\beta}_0)du$ converges almost surely to $\mathbb{E}(\int_0^t \tilde{\mathbf{K}}_1(u,\boldsymbol{\beta}_0)\lambda_1(u,\boldsymbol{\beta}_0)du)$ for any fixed $t \in \mathcal{T}$, as $n \to \infty$. Note that the integrability of the intensity process $\lambda_1(t,\boldsymbol{\beta}_0)$ follows from the integrability of the cumulative intensity process $\Lambda_1(t,\boldsymbol{\beta}_0)$. Thus, due to the integrability of the cumulative intensities and Assumption 2 (2), we can make use of Fubini's

theorem, by which we can exchange the order of integration. We can conclude that

$$\frac{1}{n}\sum_{i=1}^{n}\int_0^t \tilde{\mathbf{K}}_i(u,\boldsymbol{\beta}_0)d\Lambda_i(u,\boldsymbol{\beta}_0) \xrightarrow{\mathbb{P}} \int_0^t \mathbb{E}(\tilde{\mathbf{K}}_1(u,\boldsymbol{\beta}_0)\lambda_1(u,\boldsymbol{\beta}_0))du, \qquad (47)$$

pointwise in $t \in \mathcal{T}$, as $n \to \infty$.

Next, we show the corresponding uniform convergence in probability on $\mathcal{T}$. For this, we divide the interval $\mathcal{T} = [0,\tau]$ into L equidistant subintervals $[t_l, t_{l+1}]$ with $t_0 = 0$, $t_L = \tau$, and $l \in \{0,1,\dots,L-1\}$. The width of a subinterval is chosen such that

$$\int_{t_l}^{t_{l+1}} \mathbb{E}(\|\tilde{\mathbf{K}}_1(u,\boldsymbol{\beta}_0)\|\lambda(u,\boldsymbol{\beta}_0))du \le \delta/2$$

for all $l \in \{0,1,\dots,L-1\}$. For $t \in [0,\tau)$ we denote the lower and upper endpoint of the subinterval containing t by $t_{l(t)} = \max_{l\in\{0,1,\dots,L-1\}}\{t_l : t_l \le t\}$ and $t_{l(t)+1} = \min_{l\in\{1,\dots,L\}}\{t_l : t_l > t\}$, respectively. For $t = \tau$ we choose $t_{l(\tau)} = t_{l(\tau)+1} = \tau$. In the following derivation we make use of (47) and get

$$\begin{aligned}
&\sup_{t\in\mathcal{T}}\Big\|\frac{1}{n}\sum_{i=1}^{n}\int_0^t \tilde{\mathbf{K}}_i(u,\boldsymbol{\beta}_0)\lambda_i(u,\boldsymbol{\beta}_0)du - \int_0^t \mathbb{E}(\tilde{\mathbf{K}}_1(u,\boldsymbol{\beta}_0)\lambda_1(u,\boldsymbol{\beta}_0))du\Big\| \\
&= \sup_{t\in\mathcal{T}}\Big\|\frac{1}{n}\sum_{i=1}^{n}\int_0^t \tilde{\mathbf{K}}_i(u,\boldsymbol{\beta}_0)\lambda_i(u,\boldsymbol{\beta}_0)du - \frac{1}{n}\sum_{i=1}^{n}\int_0^{t_{l(t)}} \tilde{\mathbf{K}}_i(u,\boldsymbol{\beta}_0)\lambda_i(u,\boldsymbol{\beta}_0)du \\
&\quad + \frac{1}{n}\sum_{i=1}^{n}\int_0^{t_{l(t)}} \tilde{\mathbf{K}}_i(u,\boldsymbol{\beta}_0)\lambda_i(u,\boldsymbol{\beta}_0)du - \int_0^{t_{l(t)}} \mathbb{E}(\tilde{\mathbf{K}}_1(u,\boldsymbol{\beta}_0)\lambda_1(u,\boldsymbol{\beta}_0))du \\
&\quad + \int_0^{t_{l(t)}} \mathbb{E}(\tilde{\mathbf{K}}_1(u,\boldsymbol{\beta}_0)\lambda_1(u,\boldsymbol{\beta}_0))du - \int_0^t \mathbb{E}(\tilde{\mathbf{K}}_1(u,\boldsymbol{\beta}_0)\lambda_1(u,\boldsymbol{\beta}_0))du\Big\| \\
&\le \sup_{t\in\mathcal{T}}\Big(\Big\|\frac{1}{n}\sum_{i=1}^{n}\int_{t_{l(t)}}^t \tilde{\mathbf{K}}_i(u,\boldsymbol{\beta}_0)\lambda_i(u,\boldsymbol{\beta}_0)du - \int_{t_{l(t)}}^t \mathbb{E}(\tilde{\mathbf{K}}_1(u,\boldsymbol{\beta}_0)\lambda_1(u,\boldsymbol{\beta}_0))du\Big\|\Big) + o_p(1) \\
&\le \sup_{t\in\mathcal{T}}\Big(\frac{1}{n}\sum_{i=1}^{n}\int_{t_{l(t)}}^t \|\tilde{\mathbf{K}}_i(u,\boldsymbol{\beta}_0)\|\lambda_i(u,\boldsymbol{\beta}_0)du + \int_{t_{l(t)}}^t \mathbb{E}(\|\tilde{\mathbf{K}}_1(u,\boldsymbol{\beta}_0)\|\lambda_1(u,\boldsymbol{\beta}_0))du\Big) + o_p(1) \\
&\le \max_{l\in\{0,\dots,L-1\}}\Big(\frac{1}{n}\sum_{i=1}^{n}\int_{t_l}^{t_{l+1}} \|\tilde{\mathbf{K}}_i(u,\boldsymbol{\beta}_0)\|\lambda_i(u,\boldsymbol{\beta}_0)du \\
&\quad + \int_{t_l}^{t_{l+1}} \mathbb{E}(\|\tilde{\mathbf{K}}_1(u,\boldsymbol{\beta}_0)\|\lambda_1(u,\boldsymbol{\beta}_0))du\Big) + o_p(1) \\
&\xrightarrow{\mathbb{P}} 2\cdot \max_{l\in\{0,\dots,L-1\}}\Big(\int_{t_l}^{t_{l+1}} \mathbb{E}(\|\tilde{\mathbf{K}}_1(u,\boldsymbol{\beta}_0)\|\lambda_1(u,\boldsymbol{\beta}_0))du\Big) \le \delta, \quad n\to\infty.
\end{aligned}$$

The convergence involved in the last step of the considerations above, follows from the same arguments that led to (47). As we can choose the length of the subintervals $[t_l, t_{l+1}]$ such that $\delta > 0$ is arbitrarily small, we obtain (46). $\square$

Proof of Theorem 2.
We aim to derive the limit in law of $\mathbf{D}_{n,k} + \mathbf{B}_n\mathbf{C}_n\mathbf{D}_{n,g}(\tau)$, as $n \to \infty$, where $\mathbf{D}_{n,k}$ and $\mathbf{D}_{n,g}$ are vector-valued local square integrable martingales, $\mathbf{B}_n$ is a matrix-valued stochastic process and $\mathbf{C}_n$ is a random matrix. For this, we first show that the weak limit of $(\mathbf{D}_{n,k}^\top, \mathbf{D}_{n,g}^\top, \text{vec}(\mathbf{B}_n)^\top, \text{vec}(\mathbf{C}_n)^\top)$ is $(\mathbf{D}_{\tilde{k}}^\top, \mathbf{D}_{\tilde{g}}^\top, \text{vec}(\mathbf{B})^\top, \text{vec}(\mathbf{C})^\top)$, as $n \to \infty$. According to Lemma 5, we have

$$(\mathbf{D}_{n,k}^\top, \mathbf{D}_{n,g}^\top)^\top = \mathbf{D}_{n,h} \xrightarrow{\mathcal{L}} \mathbf{D}_{\tilde{h}} = (\mathbf{D}_{\tilde{k}}^\top, \mathbf{D}_{\tilde{g}}^\top)^\top, \quad \text{in } (D(\mathcal{T}))^{p+b}, \text{ as } n \to \infty,$$

where $\mathbf{D}_{\tilde{h}}$ is a continuous zero-mean Gaussian $(p + b)$-dimensional vector martingale with covariance function $\mathbf{V}_{\tilde{h}}(t) = \int_0^t \mathbb{E}(\tilde{\mathbf{h}}_1(u, \boldsymbol{\beta}_0)^{\otimes 2}\lambda_1(u, \boldsymbol{\beta}_0))du$, $t \in \mathcal{T}$. As $\mathbf{D}_{\tilde{h}} \in \mathcal{C}[0, \tau]^{p+b}$, we know that $\mathbf{D}_{\tilde{h}}$ is separable. Furthermore, we have shown in Lemma 6 that there exists a $p \times q$-dimensional continuous, deterministic function $\mathbf{B}(t)$, $t \in \mathcal{T}$, such that $\sup_{t\in\mathcal{T}}\|\mathbf{B}_n(t) - \mathbf{B}(t)\| \xrightarrow{\mathbb{P}} 0$, as $n \to \infty$. In other words, the limit in law $\text{vec}(\mathbf{B})$ of $\text{vec}(\mathbf{B}_n)$ is a constant of the space $\mathcal{C}[0, \tau]^{pq}$. Thus, we conclude with Example 1.4.7 of van der Vaart and Wellner [1996] that

$$(\mathbf{D}_{n,h}^\top, \text{vec}(\mathbf{B}_n)^\top) \xrightarrow{\mathcal{L}} (\mathbf{D}_{\tilde{h}}^\top, \text{vec}(\mathbf{B})^\top), \text{ in } D[0, \tau]^{p+b+pq}, \text{ as } n \to \infty.$$

As the last step of the first part of this proof we argue that

$$(\mathbf{D}_{n,h}^\top, \text{vec}(\mathbf{B}_n)^\top, \text{vec}(\mathbf{C}_n)^\top) \xrightarrow{\mathcal{L}} (\mathbf{D}_{\tilde{h}}^\top, \text{vec}(\mathbf{B})^\top, \text{vec}(\mathbf{C})^\top), \tag{48}$$

in $\mathcal{D}[0, \tau]^{p+b+pq} \times \mathbb{R}^{pq}$, as $n \to \infty$. For this, we point out that $(\mathbf{D}_{\tilde{h}}^\top, \text{vec}(\mathbf{B})^\top) \in \mathcal{C}[0, \tau]^{p+b+pq}$. Thus, $(\mathbf{D}_{\tilde{h}}^\top, \text{vec}(\mathbf{B})^\top)$ is separable. Additionally, we have assumed in Assumption I.3 that the random $q \times p$-dimensional matrix $\mathbf{C}_n$ converges in probability to the deterministic matrix $\mathbf{C}$, as $n \to \infty$. Because $\mathbf{C}_n$ is asymptotically degenerate and $(\mathbf{D}_{\tilde{h}}^\top, \text{vec}(\mathbf{B})^\top)$ is separable, we again use Example 1.4.7 of van der Vaart and Wellner [1996] and infer that (48) holds.

It only remains to apply the continuous mapping theorem to (48) in order to derive the weak limit of $\mathbf{D}_{n,k} + \mathbf{B}_n\mathbf{C}_n\mathbf{D}_{n,g}$, as $n \to \infty$. In particular, we use the following three maps

$$f_1 : (\mathbf{D}_{n,k}^\top, \mathbf{D}_{n,g}(\tau)^\top, \text{vec}(\mathbf{B}_n)^\top, \text{vec}(\mathbf{C}_n)^\top) \mapsto (\mathbf{D}_{n,k}^\top, \mathbf{D}_{n,g}(\tau)^\top, \text{vec}(\mathbf{B}_n\mathbf{C}_n)^\top)$$
$$f_2 : (\mathbf{D}_{n,k}^\top, \mathbf{D}_{n,g}(\tau)^\top, \text{vec}(\mathbf{B}_n\mathbf{C}_n)^\top) \mapsto (\mathbf{D}_{n,k}^\top, (\mathbf{B}_n\mathbf{C}_n\mathbf{D}_{n,g}(\tau))^\top)$$
$$f_3 : (\mathbf{D}_{n,k}^\top, (\mathbf{B}_n\mathbf{C}_n\mathbf{D}_{n,g}(\tau))^\top) \mapsto (\mathbf{D}_{n,k} + \mathbf{B}_n\mathbf{C}_n\mathbf{D}_{n,g}(\tau)).$$

Recall that $(\mathbf{D}_{\tilde{k}}^\top, \mathbf{D}_{\tilde{g}}^\top, \text{vec}(\mathbf{B})^\top, \text{vec}(\mathbf{C})^\top) \in \mathcal{C}[0, \tau]^{p+b+2pq}$. Thus, it follows successively with the continuous mapping theorem and the maps f_1, f_2 and f_3 that

$$\mathbf{D}_{n,k} + \mathbf{B}_n\mathbf{C}_n\mathbf{D}_{n,g}(\tau) \xrightarrow{\mathcal{L}} \mathbf{D}_{\tilde{k}} + \mathbf{B}\mathbf{C}\mathbf{D}_{\tilde{g}}(\tau) \text{ in } D[0, \tau]^p,$$

as $n \to \infty$. Moreover, the covariance function of $\mathbf{D}_{\tilde{k}} + \mathbf{B}\mathbf{C}\mathbf{D}_{\tilde{g}}(\tau)$ at $t \in \mathcal{T}$ maps t to

$$\mathbf{V}_{\tilde{k}}(t) + \mathbf{B}(t)\mathbf{C}\mathbf{V}_{\tilde{g}}(\tau)\mathbf{C}^\top\mathbf{B}(t)^\top + [\mathbf{V}_{\tilde{k},\tilde{g}}(t) + \text{Cov}(\mathbf{D}_{\tilde{k}}(t), \mathbf{D}_{\tilde{g}}(\tau) - \mathbf{D}_{\tilde{g}}(t))]\mathbf{C}^\top\mathbf{B}(t)^\top$$

$$
\begin{aligned}
&+ \mathbf{B}(t)\mathbf{C}[\mathbf{V}_{\tilde g,\tilde k}(t) + \mathrm{Cov}(\mathbf{D}_{\tilde g}(\tau) - \mathbf{D}_{\tilde g}(t), \mathbf{D}_{\tilde k}(t))] \\
&= \mathbf{V}_{\tilde k}(t) + \mathbf{B}(t)\mathbf{C}\mathbf{V}_{\tilde g}(\tau)\mathbf{C}^\top\mathbf{B}(t)^\top + \mathbf{V}_{\tilde k,\tilde g}(t)\mathbf{C}^\top\mathbf{B}(t)^\top + \mathbf{B}(t)\mathbf{C}\mathbf{V}_{\tilde g,\tilde k}(t),
\end{aligned}
$$

where $\mathrm{Cov}(\mathbf{D}_{\tilde k}(t), \mathbf{D}_{\tilde g}(\tau) - \mathbf{D}_{\tilde g}(t)) = \mathrm{Cov}(\mathbf{D}_{\tilde g}(\tau) - \mathbf{D}_{\tilde g}(t), \mathbf{D}_{\tilde k}(t))^\top = 0$, because

$$
\begin{aligned}
\mathbb{E}(\mathbf{D}_{\tilde k}(t)(\mathbf{D}_{\tilde g}(\tau) - \mathbf{D}_{\tilde g}(t))^\top) &= \mathbb{E}(\mathbb{E}(\mathbf{D}_{\tilde k}(t)(\mathbf{D}_{\tilde g}(\tau) - \mathbf{D}_{\tilde g}(t))^\top|\mathcal{F}_1(t))) \\
&= \mathbb{E}(\mathbf{D}_{\tilde k}(t)\mathbb{E}((\mathbf{D}_{\tilde g}(\tau) - \mathbf{D}_{\tilde g}(t))^\top)) \\
&= 0.
\end{aligned}
$$

Here the one but last step holds because $\sigma(\mathbf{D}_{\tilde k}(t)) \in \mathcal{F}_1(t)$ and $\mathbf{D}_{\tilde g}(\tau) - \mathbf{D}_{\tilde g}(t)$ is independent of $\mathcal{F}_1(t)$. In the last step it has been applied that $\mathbb{E}(\mathbf{D}_{\tilde g}(\tau) - \mathbf{D}_{\tilde g}(t)) = 0$. □

A.2 Proofs and Lemmas of Section 3

This section contains the proofs of all wild bootstrap-related statements which are based on martingale theory. We proceed as follows. First we prove Lemma 2. Then we show that the wild bootstrapped statistics form martingales (Lemma 3). Next, we will re-use Rebolledo's martingale central limit theorem to establish conditional weak convergence in probability. Corollary 1 below offers an additional method of proof for the convergence of the optional variation process. Lemma 9 takes care of the convergence of $\mathbf{B}_n^*$ and, finally, the proof of Theorem 3, which is stated in the main manuscript, combines all separate convergences into the one conditional central limit theorem of actual interest.

Proof of Lemma 2.
First, we derive a representation in analogy to (9) presented in Lemma 1: for $t \in \mathcal{T}$,

$$
\begin{aligned}
&\sqrt{n}(\mathbf{X}_n^*(t) - \mathbf{X}_n(t)) \\
&= \sqrt{n}\Big(\frac{1}{n}\sum_{i=1}^n \int_0^t \big[\mathbf{k}_{n,i}(u,\hat{\boldsymbol\beta}_n^*) \pm \mathbf{k}_{n,i}(u,\hat{\boldsymbol\beta}_n)\big]\big(G_i(u)+1\big)dN_i(u) \\
&\qquad - \frac{1}{n}\sum_{i=1}^n \int_0^t \mathbf{k}_{n,i}(u,\hat{\boldsymbol\beta}_n)dN_i(u)\Big) \\
&= \frac{1}{\sqrt{n}}\sum_{i=1}^n \int_0^t \mathbf{k}_{n,i}(u,\hat{\boldsymbol\beta}_n)G_i(u)dN_i(u) \\
&\qquad + \frac{1}{\sqrt{n}}\sum_{i=1}^n \int_0^t \big[\mathbf{k}_{n,i}(u,\hat{\boldsymbol\beta}_n^*) - \mathbf{k}_{n,i}(u,\hat{\boldsymbol\beta}_n)\big](G_i(u)+1)dN_i(u).
\end{aligned}
$$

A Taylor expansion around $\hat{\boldsymbol{\beta}}_n$ of the second term on the right-hand side of the last equality results in:

$$\begin{aligned}\sqrt{n}(\mathbf{X}_n^*(t) - \mathbf{X}_n(t)) = &\frac{1}{\sqrt{n}}\sum_{i=1}^n \int_0^t \mathbf{k}_{n,i}(u,\hat{\boldsymbol{\beta}}_n)G_i(u)dN_i(u)\\ &+ \frac{1}{n}\sum_{i=1}^n \int_0^t \mathrm{D}\mathbf{k}_{n,i}(u,\hat{\boldsymbol{\beta}}_n)(G_i(u)+1)dN_i(u)\sqrt{n}(\hat{\boldsymbol{\beta}}_n^* - \hat{\boldsymbol{\beta}}_n)\\ &+ \sqrt{n}o_p(\hat{\boldsymbol{\beta}}_n^* - \hat{\boldsymbol{\beta}}_n),\end{aligned} \tag{49}$$

for $t \in [0,\tau]$. Using (12), (16), (17), and Theorem 3, according to which $\hat{\boldsymbol{\beta}}_n^* - \hat{\boldsymbol{\beta}}_n = O_p(n^{-1/2})$ and hence, $o_p(\hat{\boldsymbol{\beta}}_n^* - \hat{\boldsymbol{\beta}}_n) = o_p(n^{-1/2})$ holds, (49) can be expressed as stated in Lemma 2, that is,

$$\sqrt{n}\big(\mathbf{X}_n^*(t) - \mathbf{X}_n(t)\big) = \mathbf{D}_{n,k}^*(t) + \mathbf{B}_n^*(t)\mathbf{C}_n^*\mathbf{D}_{n,g}^*(\tau) + o_p(1), t \in \mathcal{T}.$$

□

Proof of Lemma 3.
In the first part of this proof, we show that, conditionally on the initial σ-algebra $\mathcal{F}_2(0)$, the stochastic process $\mathbf{D}_{n,h}^*(t) = (D_{n,h}^{*,1}(t), \dots, D_{n,h}^{*,p+b}(t))$, $t \in \mathcal{T}$, is a $(p+b)$-dimensional vector of square integrable martingales with respect to $\mathcal{F}_2(t)$. Here, the j-th element $D_{n,h}^{*,j}$ of $\mathbf{D}_{n,h}^*$, $j = 1, \dots, p+b$, is given by

$$D_{n,h}^{*,j}(t) = \frac{1}{\sqrt{n}}\sum_{i=1}^n \int_0^t h_{n,i}^j(u,\hat{\boldsymbol{\beta}}_n)G_i(u)\,dN_i(u), \quad t \in \mathcal{T},$$

where $h_{n,i}^j(t,\hat{\boldsymbol{\beta}}_n)$ denotes the j-th element of the $(p+b)$-dimensional function $\mathbf{h}_{n,i}(t,\hat{\boldsymbol{\beta}}_n)$. For later use we write $D_{n,h}^{*,j}$ as the scaled sum over $D^{*,j}(\cdot)_{n,h,i} = \int_0^\cdot h_{n,i}^j(u,\hat{\boldsymbol{\beta}}_n)G_i(u)\,dN_i(u)$, namely $D_{n,h}^{*,j}(t) = \frac{1}{\sqrt{n}}\sum_{i=1}^n D_{n,h,i}^{*,j}(t)$, $t \in \mathcal{T}$. Furthermore, by incorporating the jump time points $T_{i,1}, \dots, T_{i,n_i}$ of the counting process N_i, we can write

$$D_{n,h}^{*,j}(t) = \frac{1}{\sqrt{n}}\sum_{i=1}^n \sum_{r:T_{i,r}\le t} h_{n,i}^j(T_{i,r},\hat{\boldsymbol{\beta}})G_i(T_{i,r}), \quad t \in \mathcal{T}.$$

Clearly, all stochastic processes $D_{n,h}^{*,j}(t)$, $t \in \mathcal{T}$, $j = 1, \dots, p+b$, are adapted to the filtration $\mathcal{F}_2(t)$, $t \in \mathcal{T}$. Moreover, for all $j = 1, \dots, p+b$, $D_{n,h}^{*,j}$ is cadlag, as the same holds for the counting processes N_i, $i = 1, \dots, n$. As we work with a probability space, square integrability implies integrability of a stochastic process. Thus, we directly show that $D_{n,h}^{*,j}$ is square integrable for all $j = 1, \dots, p+b$. For this, we wish to show that

$$\sup_{t\in\mathcal{T}} \mathbb{E}_0(D_{n,h}^{*,j}(t)^2) = \sup_{t\in\mathcal{T}} \mathbb{E}_0\Big(\frac{1}{n}\Big(\sum_{i=1}^n D_{n,h,i}^{*,j}(t)\Big)^2\Big) < \infty,$$

where $\mathbb{E}_0$ denotes the conditional expectation $\mathbb{E}(\cdot|\mathcal{F}_2(0))$. In preparation for this, we state

$$\begin{aligned}\frac{1}{n}\Big(\sum_{i=1}^{n} D^{*,j}_{n,h,i}(t)\Big)^2 &= \frac{1}{n}\sum_{i=1}^{n}\sum_{l=1}^{n} D^{*,j}_{n,h,i}(t)D^{*,j}_{n,h,l}(t)\\ &= \frac{1}{n}\sum_{i=1}^{n}\sum_{l=1}^{n}\sum_{r:T_{i,r}\le t}\sum_{v:T_{i,v}\le t} h^j_{n,i}(T_{i,r},\hat{\boldsymbol{\beta}}_n)h^j_{n,l}(T_{l,v},\hat{\boldsymbol{\beta}}_n)G_i(T_{i,r})G_l(T_{l,v}).\end{aligned} \tag{50}$$

In the next step we use that the functions $\mathbf{h}_{n,i}(t,\hat{\boldsymbol{\beta}}_n)$, $i=1,\dots,n$, are $\mathcal{F}_2(0)$-measurable. Additionally, we apply that the values of the multiplier process $G_i(t)$, $t\in\mathcal{T}^{\Delta}_{n,i}$, are independent of the σ-algebra $\mathcal{F}_2(0)$. Combining these assumptions with (50), we get

$$\begin{aligned}&\mathbb{E}_0(D^{*,j}_{n,h}(t)^2)\\ &= \frac{1}{n}\sum_{i=1}^{n}\sum_{l=1}^{n}\sum_{r:T_{i,r}\le t}\sum_{v:T_{i,v}\le t} h^j_{n,i}(T_{i,r},\hat{\boldsymbol{\beta}}_n)h^j_{n,l}(T_{l,v},\hat{\boldsymbol{\beta}}_n)\mathbb{E}(G_i(T_{i,r})G_l(T_{l,v})).\end{aligned} \tag{51}$$

By construction of the multiplier processes we have for $i\neq l$ or $\{i=l, r\neq v\}$

$$\mathbb{E}(G_i(T_{i,r})G_l(T_{l,v})) = \mathbb{E}(G_i(T_{i,r}))\mathbb{E}(G_l(T_{l,v})) = 0,$$

and for $\{i=l, r=v\}$

$$\mathbb{E}(G_i(T_{i,r})G_l(T_{l,v})) = \mathbb{E}(G_i(T_{i,r})^2) = 1.$$

Thus, (51) simplifies to $\mathbb{E}_0((D^{*,j}_{n,h}(t))^2) = \frac{1}{n}\sum_{i=1}^{n}\sum_{r:T_{i,r}\le t} h^j_{n,i}(T_{i,r},\hat{\boldsymbol{\beta}}_n)^2$. Finally, it holds that

$$\sup_{t\in\mathcal{T}}\mathbb{E}_0(D^{*,j}_{n,h}(t)^2) \le \sup_{t\in\mathcal{T}, i\in\{1,\dots,n\}} h^j_{n,i}(t,\hat{\boldsymbol{\beta}}_n)^2 \cdot \max_{i\in\{1,\dots,n\}} N_i(\tau) < \infty,$$

since $\mathbf{h}_{n,i}(t,\hat{\boldsymbol{\beta}}_n)$ is a known function and hence, all components $h^j_{n,i}(t,\hat{\boldsymbol{\beta}}_n)$, $j=1,\dots,p+b$, are bounded on $\mathcal{T}$. Moreover, the observed number of events within the time frame $\mathcal{T}=[0,\tau]$, $N_i(\tau)$, is finite for all individuals $i=1,\dots,n$. In conclusion, $D^{*,j}_{n,h}(t)$, $t\in\mathcal{T}$, is square integrable for all $j=1,\dots,p+b$, given the initial σ-algebra $\mathcal{F}_2(0)$.

Next, we consider the martingale property for the stochastic process $D^{*,j}_{n,h}(t)$, $t\in\mathcal{T}$. Due to the linearity of the conditional expectation, is suffices to verify the martingale property for the summands $D^{*,j}_{n,h,i}(t)$ of the scaled sum $D^{*,j}_{n,h}(t)$, $i=1,\dots,n$. For this, we recall that the function $\mathbf{h}_{n,i}(t,\hat{\boldsymbol{\beta}}_n)$ and the counting process $N_i(t)$ are $\mathcal{F}_2(0)\subset\mathcal{F}_2(t)$-measurable for $t\in\mathcal{T}$, respectively, $i=1,\dots,n$. Furthermore, for a jump at $u\le s$, the multiplier process $G_i(u)$ is $\mathcal{F}_2(s)$-measurable, and, if u is greater than or equal to the earliest jump time point, say $T_i(s^+)$, of process i in $(s,\tau]$, the values of $G_i(u)$ and the filtration

$\mathcal{F}_2(s)$ are independent, $i = 1, \ldots, n$. Moreover, we use that the multiplier process $G_i(t)$, $t \in \mathcal{T}$, has mean zero. This yields for any $t > s$,

$$
\begin{aligned}
&\mathbb{E}[D^{*,j}_{n,h,i}(t)|\mathcal{F}_2(s)] \\
&= \mathbb{E}\Big[\int_0^t h^j_{n,i}(u, \hat{\boldsymbol{\beta}}_n) G_i(u)\, dN_i(u) | \mathcal{F}_2(s)\Big] \\
&= \mathbb{E}\Big[\int_0^s h^j_{n,i}(u, \hat{\boldsymbol{\beta}}_n) G_i(u)\, dN_i(u) + \int_s^t h^j_{n,i}(u, \hat{\boldsymbol{\beta}}_n) G_i(u)\, dN_i(u) \Big| \mathcal{F}_2(s)\Big] \\
&= D^{*,j}_{n,h,i}(s) + \int_s^t h^j_{n,i}(u, \hat{\boldsymbol{\beta}}_n)\, \mathbb{E}(G_i(u)|\mathcal{F}_2(s))\, dN_i(u) \\
&= D^{*,j}_{n,h,i}(s) + \int_{T_i(s^+)}^t h^j_{n,i}(u, \hat{\boldsymbol{\beta}}_n)\, \mathbb{E}(G_i(u))\, dN_i(u) \\
&= D^{*,j}_{n,h,i}(s).
\end{aligned}
$$

Thus, we have shown that all elements $D^{*,j}_{n,h}$ of $\mathbf{D}^*_{n,h}$, $j = 1, \ldots, p+b$, fulfill the martingale property. In conclusion, the stochastic process $\mathbf{D}^*_{n,h}$ is a $(p+b)$-dimensional vector of square integrable martingales with respect to $\mathcal{F}_2(t)$, $t \in \mathcal{T}$. With this the first part of Lemma 3 has been proven.

In the second part of this proof we derive the predictable covariation process $\langle \mathbf{D}^*_{n,h} \rangle$ and the optional covariation process $[\mathbf{D}^*_{n,h}]$ of $\mathbf{D}^*_{n,h}$. First, we consider the predictable covariation process $\langle \mathbf{D}^*_{n,h} \rangle(t)$:

$$
\begin{aligned}
\langle \mathbf{D}^*_{n,h} \rangle &= \frac{1}{n} \Big\langle \sum_{i=1}^n (D^{*,1}_{n,h,i}, \ldots, D^{*,p+b}_{n,h,i}) \Big\rangle \\
&= \frac{1}{n} \Big(\Big\langle \sum_{i=1}^n D^{*,j}_{n,h,i}, \sum_{i=1}^n D^{*,r}_{n,h,i} \Big\rangle \Big)_{j,r=1}^{p+b} \\
&= \frac{1}{n} \Big(\sum_{i=1}^n \sum_{l=1}^n \langle D^{*,j}_{n,h,i}, D^{*,r}_{n,h,l} \rangle \Big)_{j,r=1}^{p+b} \\
&= \frac{1}{n} \sum_{i=1}^n \sum_{l=i} \big(\langle D^{*,j}_{n,h,i}, D^{*,r}_{n,h,l} \rangle \big)_{j,r=1}^{p+b} \quad + \quad \frac{1}{n} \sum_{i=1}^n \sum_{l \neq i} \big(\langle D^{*,j}_{n,h,i}, D^{*,r}_{n,h,l} \rangle \big)_{j,r=1}^{p+b},
\end{aligned}
\tag{52}
$$

where in the second step of (52) we used that the predictable covariation process of a vector valued martingale is the matrix of the predictable covariation processes of its components. In the following we consider the predictable covariation processes $\langle D^{*,j}_{n,h,i}, D^{*,r}_{n,h,l} \rangle$ for $i = l$ and $i \neq l$ separately. Recall that the functions $\mathbf{h}_{n,i}(t, \hat{\boldsymbol{\beta}}_n)$ and the counting processes N_i are $\mathcal{F}_2(0) \subset \mathcal{F}_2(t)$-measurable, respectively, and that the values of the multiplier processes $G_i(t)$, $t \in \mathcal{T}$, are independent of the σ-algebra $\mathcal{F}_2(t-)$, $i = 1, \ldots, n$. We then get for

$i = l$,

$$\begin{aligned}
&\langle D^{*,j}_{n,h,i}, D^{*,r}_{n,h,i}\rangle(t) \\
&= \int_0^t \operatorname{Cov}\big(dD^{*,j}_{n,h,i}(u), dD^{*,r}_{n,h,i}(u)|\mathcal{F}_2(u-)\big) \\
&= \int_0^t \operatorname{Cov}\big(h^j_{n,i}(u,\hat{\boldsymbol{\beta}}_n)\, G_i(u)\, dN_i(u), h^r_{n,i}(u,\hat{\boldsymbol{\beta}}_n)\, G_i(u)\, dN_i(u)|\mathcal{F}_2(u-)\big) \\
&= \int_0^t h^j_{n,i}(u,\hat{\boldsymbol{\beta}}_n) h^r_{n,i}(u,\hat{\boldsymbol{\beta}}_n) \operatorname{Var}(G_i(u)) dN_i(u) \\
&= \int_0^t h^j_{n,i}(u,\hat{\boldsymbol{\beta}}_n) h^r_{n,i}(u,\hat{\boldsymbol{\beta}}_n) dN_i(u),
\end{aligned} \tag{53}$$

where for the last equation above we have used that the multiplier processes $G_i(t)$, $t \in \mathcal{T}$, have unit variance, $i = 1, \ldots, n$.

For $i \neq l$ it holds that

$$\begin{aligned}
d\langle D^{*,j}_{n,h,i}, D^{*,r}_{n,h,l}\rangle(t) &= \operatorname{Cov}\big(dD^{*,j}_{n,h,i}(u), dD^{*,r}_{n,h,l}(u)|\mathcal{F}_2(u-)\big) \\
&= \operatorname{Cov}\big(h^j_{n,i}(u,\hat{\boldsymbol{\beta}}_n) G_i(u) dN_i(u), h^r_{n,l}(u,\hat{\boldsymbol{\beta}}_n) G_l(u) dN_l(u)|\mathcal{F}_2(u-)\big) \\
&= h^j_{n,i}(u,\hat{\boldsymbol{\beta}}_n) h^r_{n,l}(u,\hat{\boldsymbol{\beta}}_n) \operatorname{Cov}\big(G_i(u), G_l(u)\big) dN_i(u) dN_l(u) \\
&= 0,
\end{aligned} \tag{54}$$

where in the last step we have applied that the multiplier processes $G_1(t), \ldots, G_n(t)$, $t \in \mathcal{T}$, are pairwise independent and no two processes jump simultaneously. Hence, $\langle D^{*,j}_{n,h,i}, D^{*,r}_{n,h,l}\rangle(t) = 0$ for $i \neq l$. Combining (52), (53), and (54), we can state the final form of the predictable covariation process $\langle \mathbf{D}^*_{n,h}\rangle$ of $\mathbf{D}^*_{n,h}$ at $t \in \mathcal{T}$ in matrix notation

$$\langle \mathbf{D}^*_{n,h}\rangle(t) = \frac{1}{n}\sum_{i=1}^n \int_0^t \big(h^j_{n,i}(u,\hat{\boldsymbol{\beta}}_n) h^r_{n,i}(u,\hat{\boldsymbol{\beta}}_n)\big)_{j,r=1}^{p+b} dN_i(u) = \frac{1}{n}\sum_{i=1}^n \int_0^t \mathbf{h}_{n,i}(u,\hat{\boldsymbol{\beta}}_n)^{\otimes 2} dN_i(u),$$

which proves the second part of Lemma 3.

For the optional covariation process $[\mathbf{D}^*_{n,h}]$ of $\mathbf{D}^*_{n,h}$ we can write analogously to (52)

$$[\mathbf{D}^*_{n,h}](t) = \frac{1}{n}\sum_{i=1}^n \sum_{l=i} \big([D^{*,j}_{n,h,i}, D^{*,r}_{n,h,l}](t)\big)_{j,r=1}^{p+b} + \frac{1}{n}\sum_{i=1}^n \sum_{l\neq i} \big([D^{*,j}_{n,h,i}, D^{*,r}_{n,h,l}](t)\big)_{j,r=1}^{p+b}. \tag{55}$$

Again, we consider the optional covariation process $[D^{*,j}_{n,h,i}, D^{*,r}_{n,h,l}]$ for $i = l$ and $i \neq l$ separately. For $i = l$ we get

$$\begin{aligned}
[D^{*,j}_{n,h,i}, D^{*,r}_{n,h,i}](t) &= \sum_{u \le t} \Delta D^{*,j}_{n,h,i}(u) \Delta D^{*,r}_{n,h,i}(u) \\
&= \sum_{u \le t} h^j_{n,i}(u, \hat{\boldsymbol{\beta}}_n)\, G_i(u)\, \Delta N_i(u) h^r_{n,i}(u, \hat{\boldsymbol{\beta}}_n)\, G_i(u)\, \Delta N_i(u) \\
&= \int_0^t h^j_{n,i}(u, \hat{\boldsymbol{\beta}}_n) h^r_{n,i}(u, \hat{\boldsymbol{\beta}}_n)\, G_i^2(u)\, dN_i(u).
\end{aligned} \tag{56}$$

For $i \neq l$ it holds that

$$\begin{aligned}
[D^{*,j}_{n,h,i}, D^{*,r}_{n,h,l}](t) &= \sum_{u \le t} \Delta D^{*,j}_{n,h,i}(u) \Delta D^{*,r}_{n,h,l}(u) \\
&= \sum_{u \le t} h^j_{n,i}(u, \hat{\boldsymbol{\beta}}_n)\, G_i(u)\, \Delta N_i(u) h^r_{n,l}(u, \hat{\boldsymbol{\beta}}_n)\, G_l(u)\, \Delta N_l(u) \\
&= 0,
\end{aligned} \tag{57}$$

where in the last step of the equation above we have used that no two counting processes jump at the same time. Combining (55), (56), and (57), we find for the optional covariation process $[\mathbf{D}^*_{n,h}]$ of $\mathbf{D}^*_{n,h}$ at $t \in \mathcal{T}$ in matrix notation:

$$\begin{aligned}
[\mathbf{D}^*_{n,h}](t) &= \frac{1}{n} \sum_{i=1}^n \int_0^t \left(h^j_{n,i}(u, \hat{\boldsymbol{\beta}}_n) h^r_{n,i}(u, \hat{\boldsymbol{\beta}}_n) \right)_{j,r=1}^{p+b} G_i(u)^2 dN_i(u) \\
&= \frac{1}{n} \sum_{i=1}^n \int_0^t \mathbf{h}_{n,i}(u, \hat{\boldsymbol{\beta}}_n)^{\otimes 2}\, G_i(u)^2 dN_i(u),
\end{aligned}$$

which proves the third part of Lemma 3 and the proof of the lemma is complete. □

The following lemma takes care of the convergence of the predictable covariation process of $\mathbf{D}^*_{n,h}$, as required in Condition 1 of Theorem 1.

Lemma 7 *If Assumption 1 holds, then, conditionally on $\mathcal{F}_2(0)$, $\langle \boldsymbol{D}^*_{n,h} \rangle(t) \xrightarrow{\mathbb{P}} \boldsymbol{V}_{\tilde{h}}(t)$, as $n \to \infty$, for all $t \in \mathcal{T}$, with $\boldsymbol{V}_{\tilde{h}}$ as defined in Lemma 5.*

Proof of Lemma 7.
According to Lemma 3, $\mathbf{D}^*_{n,h}$ is a vector of square integrable martingales and its predictable covariation process takes the form

$$\langle \mathbf{D}^*_{n,h} \rangle(t) = \langle \frac{1}{\sqrt{n}} \sum_{i=1}^n \int_0^{\cdot} \mathbf{h}_{n,i}(u, \hat{\boldsymbol{\beta}}_n) G_i(u)\, dN_i(u) \rangle(t)$$

$$= \frac{1}{n} \sum_{i=1}^{n} \int_0^t \mathbf{h}_{n,i}(u, \hat{\boldsymbol{\beta}}_n)^{\otimes 2} \, dN_i(u)$$
$$= \frac{1}{n} \sum_{i=1}^{n} \int_0^t \mathbf{h}_{n,i}(u, \hat{\boldsymbol{\beta}}_n)^{\otimes 2} \, (dM_i(u) + d\Lambda_i(u, \boldsymbol{\beta}_0)),$$

where in the third step we have used the Doob-Meyer decomposition with M_i a square integrable martingale with respect to $\mathcal{F}_1$ and $\Lambda_i(\cdot, \boldsymbol{\beta}_0)$ its compensator. Note the similarity of the integral with respect to $\Lambda_i(t, \boldsymbol{\beta}_0)$ to that of $\langle \mathbf{D}_{n,h} \rangle(t)$ in (40), the only difference being that the integrand is evaluated at $\hat{\boldsymbol{\beta}}_n$ instead of at $\boldsymbol{\beta}_0$. We make use of the result about $\langle \mathbf{D}_{n,h} \rangle(t)$ and consider

$$\begin{aligned} &\frac{1}{n} \sum_{i=1}^{n} \int_0^t \mathbf{h}_{n,i}(u, \hat{\boldsymbol{\beta}}_n)^{\otimes 2} \, d\Lambda_i(u, \boldsymbol{\beta}_0) - \langle \mathbf{D}_{n,h} \rangle(t) + \langle \mathbf{D}_{n,h} \rangle(t) \\ &= \frac{1}{n} \sum_{i=1}^{n} \int_0^t [\mathbf{h}_{n,i}(u, \hat{\boldsymbol{\beta}}_n)^{\otimes 2} - \mathbf{h}_{n,i}(u, \boldsymbol{\beta}_0)^{\otimes 2}] \, d\Lambda_i(u, \boldsymbol{\beta}_0) + \langle \mathbf{D}_{n,h} \rangle(t), \end{aligned} \tag{58}$$

where the first term on the right-hand side can be bounded from above by considering its largest component.

$$\begin{aligned} &\| \frac{1}{n} \sum_{i=1}^{n} \int_0^t [\mathbf{h}_{n,i}(u, \hat{\boldsymbol{\beta}}_n)^{\otimes 2} - \mathbf{h}_{n,i}(u, \boldsymbol{\beta}_0)^{\otimes 2}] \, d\Lambda_i(u, \boldsymbol{\beta}_0) \|_\infty \\ &\leq \sup_{i \in \{1,\dots,n\}, t \in \mathcal{T}} \| \mathbf{h}_{n,i}(u, \hat{\boldsymbol{\beta}}_n)^{\otimes 2} - \tilde{\mathbf{h}}_i(t, \boldsymbol{\beta}_0)^{\otimes 2} + \tilde{\mathbf{h}}_i(t, \boldsymbol{\beta}_0)^{\otimes 2} - \mathbf{h}_{n,i}(u, \boldsymbol{\beta}_0)^{\otimes 2} \|_\infty \\ &\quad \times \frac{1}{n} \sum_{i=1}^{n} \Lambda_i(t, \boldsymbol{\beta}_0) \\ &\leq \Big(\sup_{i \in \{1,\dots,n\}, t \in \mathcal{T}} \| (\mathbf{h}_{n,i}(t, \hat{\boldsymbol{\beta}}_n) - \tilde{\mathbf{h}}_i(t, \boldsymbol{\beta}_0)) \mathbf{h}_{n,i}(t, \hat{\boldsymbol{\beta}}_n)^\top \|_\infty \\ &\qquad + \sup_{i \in \{1,\dots,n\}, t \in \mathcal{T}} \| \tilde{\mathbf{h}}_i(t, \boldsymbol{\beta}_0) (\mathbf{h}_{n,i}(t, \hat{\boldsymbol{\beta}}_n) - \tilde{\mathbf{h}}_i(t, \boldsymbol{\beta}_0))^\top \|_\infty \\ &\qquad + \sup_{i \in \{1,\dots,n\}, t \in \mathcal{T}} \| (\mathbf{h}_{n,i}(t, \boldsymbol{\beta}_0) - \tilde{\mathbf{h}}_i(t, \boldsymbol{\beta}_0)) \mathbf{h}_{n,i}(t, \boldsymbol{\beta}_0)^\top \|_\infty \\ &\qquad + \sup_{i \in \{1,\dots,n\}, t \in \mathcal{T}} \| \tilde{\mathbf{h}}_i(t, \boldsymbol{\beta}_0) (\mathbf{h}_{n,i}(t, \boldsymbol{\beta}_0) - \tilde{\mathbf{h}}_i(t, \boldsymbol{\beta}_0))^\top \|_\infty \Big) \frac{1}{n} \sum_{i=1}^{n} \Lambda_i(t, \boldsymbol{\beta}_0). \end{aligned}$$

All four terms in brackets converge to zero in probability, as $n \to \infty$, according to Assumption 1 (1), (2), and the fact that $\mathbf{h}_{n,i}(t, \boldsymbol{\beta}_0)$ and $\mathbf{h}_{n,i}(t, \hat{\boldsymbol{\beta}}_n)$ are (locally) bounded. In the following we make use of results of the proof of Lemma 5. For this we note that convergence in probability is equivalent to convergence in conditional probability, cf. Fact 1 of the supplement of Dobler et al. [2019]. As stated in the proof of Lemma 5, $\frac{1}{n} \sum_{i=1}^{n} \Lambda_i(t, \boldsymbol{\beta}_0) = O_p(1)$, according to Assumption 1 (3), the integrability of $\Lambda_i(t, \boldsymbol{\beta}_0)$ and the law of large numbers. Hence, the first term on the right-hand side of (58) converges to zero in probability, as $n \to \infty$. Additionally, according to Assumption 1 (2), (3),

the integrability of $\Lambda_i(t, \boldsymbol{\beta}_0)$ and the law of large numbers, we have shown in the proof of Lemma 5 that

$$\langle \mathbf{D}_{n,h} \rangle(t) \xrightarrow{\mathbb{P}} \int_0^t \mathbb{E}\Big(\tilde{\mathbf{h}}_1(u, \boldsymbol{\beta}_0)^{\otimes 2} \lambda_1(u, \boldsymbol{\beta}_0)\Big) du = \mathbf{V}_{\tilde{h}}(t), \text{ for all } t \in \mathcal{T}, \text{ as } n \to \infty.$$

In particular, $\frac{1}{n}\sum_{i=1}^n \int_0^t \mathbf{h}_{n,i}(u, \hat{\boldsymbol{\beta}}_n)^{\otimes 2}\, d\Lambda_i(u, \boldsymbol{\beta}_0)$ and $\langle \mathbf{D}_{n,h} \rangle(t)$ are asymptotically equivalent.

Next, we consider the integral with respect to the local square integrable martingale M_i, $i = 1, \ldots, n$. As, conditionally on $\mathcal{F}_2(0)$, the integrands $\mathbf{h}_{n,i}(\cdot, \hat{\boldsymbol{\beta}})^{\otimes 2}$, $i = 1, \ldots, n$, are known and, hence, predictable with respect to $\mathcal{F}_2$ and locally bounded, the corresponding integral $\mathbf{W}_n(t) = \frac{1}{n}\sum_{i=1}^n \int_0^t \mathbf{h}_{n,i}(u, \hat{\boldsymbol{\beta}})^{\otimes 2}\, dM_i(u)$ is a local square integrable martingale (Proposition II.4.1, Andersen et al. 1993, p. 78). Hence, we apply Lenglart's inequality in order to show that $\mathbf{W}_n(t)$ converges to zero in probability for all $t \in \mathcal{T}$, as $n \to \infty$. For this purpose, we consider its predictable covariation process

$$\begin{aligned}
\langle \mathrm{vec}(\mathbf{W}_n) \rangle(\tau) &= \langle \frac{1}{n}\sum_{i=1}^n \int_0^{\cdot} \mathrm{vec}(\mathbf{h}_{n,i}(u, \hat{\boldsymbol{\beta}})^{\otimes 2})\, dM_i(u) \rangle(\tau) \\
&= \frac{1}{n^2}\sum_{i=1}^n \int_0^{\tau} \mathrm{vec}(\mathbf{h}_{n,i}(u, \hat{\boldsymbol{\beta}})^{\otimes 2})^{\otimes 2}\, d\Lambda_i(u, \boldsymbol{\beta}_0), \\
&= \frac{1}{n^2}\sum_{i=1}^n \int_0^{\tau} [\mathrm{vec}(\mathbf{h}_{n,i}(u, \hat{\boldsymbol{\beta}})^{\otimes 2})^{\otimes 2} - \mathrm{vec}(\tilde{\mathbf{h}}_i(u, \boldsymbol{\beta}_0)^{\otimes 2})^{\otimes 2}]\, d\Lambda_i(u, \boldsymbol{\beta}_0) \\
&\quad + \frac{1}{n^2}\sum_{i=1}^n \int_0^{\tau} \mathrm{vec}(\tilde{\mathbf{h}}_i(u, \boldsymbol{\beta}_0)^{\otimes 2})^{\otimes 2}\, d\Lambda_i(u, \boldsymbol{\beta}_0),
\end{aligned} \tag{59}$$

where in the second equality it has been used that the martingales $M_1(t), \ldots, M_n(t)$ are independent. We wish to show that the first term on the right-hand side of the third step converges to zero in probability, as $n \to \infty$. For this, it suffices to consider the largest component

$$\begin{aligned}
&\frac{1}{n^2}\sum_{i=1}^n \int_0^{\tau} \|\mathrm{vec}(\mathbf{h}_{n,i}(u, \hat{\boldsymbol{\beta}}_n)^{\otimes 2})^{\otimes 2} - \mathrm{vec}(\tilde{\mathbf{h}}_i(u, \boldsymbol{\beta}_0)^{\otimes 2})^{\otimes 2}\|_\infty\, d\Lambda_i(u, \boldsymbol{\beta}_0) \\
&\le \sup_{i \in \{1\ldots,n\}, t \in \mathcal{T}} \|\mathrm{vec}(\mathbf{h}_{n,i}(t, \hat{\boldsymbol{\beta}}_n)^{\otimes 2})^{\otimes 2} - \mathrm{vec}(\tilde{\mathbf{h}}_i(t, \boldsymbol{\beta}_0)^{\otimes 2})^{\otimes 2}\|_\infty \frac{1}{n^2}\sum_{i=1}^n \Lambda_i(\tau, \boldsymbol{\beta}_0).
\end{aligned}$$

It holds that

$$\begin{aligned}
&\|\mathrm{vec}(\mathbf{h}_{n,i}(t, \hat{\boldsymbol{\beta}}_n)^{\otimes 2})^{\otimes 2} - \mathrm{vec}(\tilde{\mathbf{h}}_i(t, \boldsymbol{\beta}_0)^{\otimes 2})^{\otimes 2}\|_\infty \\
\le\ & \|\mathbf{h}_{n,i}(t, \hat{\boldsymbol{\beta}}_n)\|_\infty^2 \Big[\|\mathbf{h}_{n,i}(t, \hat{\boldsymbol{\beta}}_n) - \tilde{\mathbf{h}}_i(t, \boldsymbol{\beta}_0)\|_\infty \|\mathbf{h}_{n,i}(t, \hat{\boldsymbol{\beta}}_n)\|_\infty \\
&+ \|\tilde{\mathbf{h}}_i(t, \boldsymbol{\beta}_0)\|_\infty \|\mathbf{h}_{n,i}(t, \hat{\boldsymbol{\beta}}_n) - \tilde{\mathbf{h}}_i(t, \boldsymbol{\beta}_0)\|_\infty\Big]
\end{aligned}$$

$$+ \|\tilde{\mathbf{h}}_i(t,\boldsymbol{\beta}_0)\|_\infty^2 \Big[\|\mathbf{h}_{n,i}(t,\hat{\boldsymbol{\beta}}_n) - \tilde{\mathbf{h}}_i(t,\boldsymbol{\beta}_0)\|_\infty \|\mathbf{h}_{n,i}(t,\hat{\boldsymbol{\beta}}_n)\|_\infty$$
$$+ \|\tilde{\mathbf{h}}_i(t,\boldsymbol{\beta}_0)\|_\infty \|\mathbf{h}_{n,i}(t,\hat{\boldsymbol{\beta}}_n) - \tilde{\mathbf{h}}_i(t,\boldsymbol{\beta}_0)\|_\infty\Big],$$

where we used the triangle inequality and applied $\mathbf{a}^{\otimes 2} - \mathbf{b}^{\otimes 2} = (\mathbf{a}-\mathbf{b})\mathbf{a}^\top + \mathbf{b}(\mathbf{a}-\mathbf{b})^\top$ for two vectors $\mathbf{a},\mathbf{b}$ twice, i.e.,

$$\begin{aligned} \mathrm{vec}[\mathbf{a}^{\otimes 2}]^{\otimes 2} - \mathrm{vec}[\mathbf{b}^{\otimes 2}]^{\otimes 2} = \mathrm{vec}[(\mathbf{a}-\mathbf{b})\mathbf{a}^\top + \mathbf{b}(\mathbf{a}-\mathbf{b})^\top]\mathrm{vec}[\mathbf{a}\mathbf{a}^\top]^\top \\ + \mathrm{vec}[\mathbf{b}\mathbf{b}^\top]\mathrm{vec}[(\mathbf{a}-\mathbf{b})\mathbf{a}^\top + \mathbf{b}(\mathbf{a}-\mathbf{b})^\top]^\top. \end{aligned} \tag{60}$$

Hence, according to Assumption 1 (1), (2), and since $\mathbf{h}_{n,i}(t,\hat{\boldsymbol{\beta}}_n)$ is locally bounded for $i = 1,\ldots,n$, it follows that $\sup_{i\in\{1\ldots,n\},t\in\mathcal{T}}\|\mathrm{vec}(\mathbf{h}_{n,i}(u,\hat{\boldsymbol{\beta}}_n)^{\otimes 2})^{\otimes 2} - \mathrm{vec}(\tilde{\mathbf{h}}_i(u,\boldsymbol{\beta}_0)^{\otimes 2})^{\otimes 2}\|_\infty = o_p(1)$. As explained before, we have $\frac{1}{n}\sum_{i=1}^n \Lambda_i(\tau,\boldsymbol{\beta}_0) = O_p(1)$. In conclusion, the first term on the right-hand side of the third step of (59) converges to zero in probability, as $n\to\infty$.

We futher need to show that the corresponding second term vanishes asymptotically. For this we consider the largest component of $\mathbb{E}\Big(\int_0^\tau \mathrm{vec}(\tilde{\mathbf{h}}_1(u,\boldsymbol{\beta}_0)^{\otimes 2})^{\otimes 2}\, d\Lambda_1(u,\boldsymbol{\beta}_0)\Big)$, for which it holds that

$$\mathbb{E}(\int_0^\tau \|\tilde{\mathbf{h}}_1(u,\boldsymbol{\beta}_0)\|_\infty^4\, d\Lambda_1(u,\boldsymbol{\beta}_0)) = \mathbb{E}(\sup_{t\in\mathcal{T}}\|\tilde{\mathbf{h}}_1(t,\boldsymbol{\beta}_0)\|_\infty^4 \Lambda_1(\tau,\boldsymbol{\beta}_0)) < \infty,$$

due to Assumption 1 (2) and the integrability of $\Lambda_i(\tau,\boldsymbol{\beta}_0)$. Combining this with Assumption 1 (3) and the law of large numbers yields

$$\frac{1}{n}\sum_{i=1}^n \int_0^\tau \mathrm{vec}(\tilde{\mathbf{h}}_i(u,\boldsymbol{\beta}_0)^{\otimes 2})^{\otimes 2}\, d\Lambda_i(u,\boldsymbol{\beta}_0) \xrightarrow{\mathbb{P}} \mathbb{E}\Big(\int_0^\tau \mathrm{vec}(\tilde{\mathbf{h}}_1(u,\boldsymbol{\beta}_0)^{\otimes 2})^{\otimes 2}\, d\Lambda_1(u,\boldsymbol{\beta}_0)\Big),$$

as $n\to\infty$. Finally, for the second term on the right-hand side of the third step of (59) we have $\frac{1}{n^2}\sum_{i=1}^n \int_0^\tau \mathrm{vec}(\tilde{\mathbf{h}}_i(t,\boldsymbol{\beta}_0)^{\otimes 2})^{\otimes 2}\, d\Lambda_i(u,\boldsymbol{\beta}_0) = o(1)\cdot O_p(1)$. Thus, $\mathbf{W}_n(t)$ converges to zero in probability for all $t\in\mathcal{T}$, as $n\to\infty$, according to Lenglart's inequality. In conclusion, the predictable covariation process $\langle \mathbf{D}^*_{n,h}\rangle(t)$ of $\mathbf{D}^*_{n,h}$ at t converges to the matrix-valued function $\mathbf{V}_{\tilde{h}}(t) = \int_0^t \mathbb{E}(\tilde{\mathbf{h}}_1(u,\boldsymbol{\beta}_0)^{\otimes 2}\,\lambda_1(u,\boldsymbol{\beta}_0))du$ in probability, as $n\to\infty$, for all $t\in\mathcal{T}$. This completes the proof of Lemma 7. □

Based on the prepared martingale theory, we now study the convergence in law of the process $\mathbf{D}^*_{n,h}$ in the upcoming Lemma 8. From Lemmas 5 and 7, it follows that the predictable variation process $\langle\mathbf{D}^*_{n,h}\rangle$ of $\mathbf{D}^*_{n,h}$ converges to the same matrix-valued function $\mathbf{V}_{\tilde{h}}$ as the predictable variation process $\langle\mathbf{D}_{n,h}\rangle$ of $\mathbf{D}_{n,h}$. This gives rise to the supposition that those two processes converge in distribution to the same Gaussian martingale. In fact, we show that the

conditional distribution of $\mathbf{D}^*_{n,h}$ asymptotically coincides with the distribution of $\mathbf{D}_{n,h}$.

Lemma 8 *If Assumption 1 holds, then, conditionally on $\mathcal{F}_2(0)$, $\boldsymbol{D}^*_{n,h} \xrightarrow{\mathcal{L}} \boldsymbol{D}_{\tilde{h}}$ in $(D(\mathcal{T}))^{p+b}$, as $n \to \infty$ in probability, with $\boldsymbol{D}_{\tilde{h}} = (\boldsymbol{D}_{\tilde{k}}, \boldsymbol{D}_{\tilde{g}})$ as given in Lemma 5.*

Proof of Lemma 8.
We use the modified version of Rebolledo's central limit theorem as stated in Theorem 1 to prove the weak convergence of $\mathbf{D}^*_{n,h}$ to the zero-mean Gaussian martingale $\mathbf{D}_{\tilde{h}}$. For this purpose, we first consider the term $\sigma^\epsilon[\boldsymbol{\lambda}^\top \mathbf{D}^*_{n,h}](\tau)$ for some $\boldsymbol{\lambda} \in S^{p+b-1}$, where S^{p+b-1} denotes the unit $(p+b-1)$-sphere. It can be seen that

$$\begin{aligned}
&\sigma^\epsilon[\boldsymbol{\lambda}^\top \mathbf{D}^*_{n,h}](\tau) \\
&= \sum_{u \le \tau} |\Delta \boldsymbol{\lambda}^\top \mathbf{D}^*_{n,h}(u)|^2 \mathbb{1}\{|\Delta \boldsymbol{\lambda}^\top \mathbf{D}^*_{n,h}(u)| > \epsilon\} \\
&= \sum_{u \le \tau} |\frac{1}{\sqrt{n}} \sum_{i=1}^n \boldsymbol{\lambda}^\top \mathbf{h}_{n,i}(u, \hat{\boldsymbol{\beta}}_n) G_i(u)\, \Delta N_i(u)|^2 \mathbb{1}\{|\frac{1}{\sqrt{n}} \sum_{i=1}^n \boldsymbol{\lambda}^\top \mathbf{h}_{n,i}(u, \hat{\boldsymbol{\beta}}_n) G_i(u)\, \Delta N_i(u)| > \epsilon\} \\
&\le \frac{1}{n} \sum_{u \le \tau} \sum_{i=1}^n |\boldsymbol{\lambda}^\top \mathbf{h}_{n,i}(u, \hat{\boldsymbol{\beta}}_n) G_i(u)\, \Delta N_i(u)|^2 \mathbb{1}\{|\frac{1}{\sqrt{n}} \sum_{i=1}^n \boldsymbol{\lambda}^\top \mathbf{h}_{n,i}(u, \hat{\boldsymbol{\beta}}_n) G_i(u)\, \Delta N_i(u)| > \epsilon\} \\
&= \frac{1}{n} \sum_{i=1}^n \sum_{j: T_{i,j} \in \mathcal{T}^\Delta_{n,i}} (\boldsymbol{\lambda}^\top \mathbf{h}_{n,i}(T_{i,j}, \hat{\boldsymbol{\beta}}_n))^2 G_i^2(T_{i,j}) \mathbb{1}\{|\frac{1}{\sqrt{n}} \boldsymbol{\lambda}^\top \mathbf{h}_{n,i}(T_{i,j}, \hat{\boldsymbol{\beta}}_n) G_i(T_{i,j})| > \epsilon\},
\end{aligned}$$

where in the third step of the derivation above it has been used that no two counting processes jump at the same time, i.e., $\Delta N_i(t) \Delta N_j(t) = 0$, for $i \neq j$. From this it follows that

$$\begin{aligned}
&\mathbb{E}_0(\sigma^\epsilon[\boldsymbol{\lambda}^\top \mathbf{D}^*_{n,h}](\tau)) \\
&\le \mathbb{E}_0(\frac{1}{n} \sum_{i=1}^n \sum_{j: T_{i,j} \in \mathcal{T}^\Delta_{n,i}} (\boldsymbol{\lambda}^\top \mathbf{h}_{n,i}(T_{i,j}, \hat{\boldsymbol{\beta}}_n))^2 G_i^2(T_{i,j}) \mathbb{1}\{|\frac{1}{\sqrt{n}} \boldsymbol{\lambda}^\top \mathbf{h}_{n,i}(T_{i,j}, \hat{\boldsymbol{\beta}}_n) G_i(T_{i,j})| > \epsilon\}) \\
&= \frac{1}{n} \sum_{i=1}^n \sum_{j: T_{i,j} \in \mathcal{T}^\Delta_{n,i}} (\boldsymbol{\lambda}^\top \mathbf{h}_{n,i}(T_{i,j}, \hat{\boldsymbol{\beta}}_n))^2 \mathbb{E}_0(G_i^2(T_{i,j}) \mathbb{1}\{|\frac{1}{\sqrt{n}} \boldsymbol{\lambda}^\top \mathbf{h}_{n,i}(T_{i,j}, \hat{\boldsymbol{\beta}}_n) G_i(T_{i,j})| > \epsilon\}) \\
&\le \frac{1}{n} \sum_{i=1}^n \sum_{j: T_{i,j} \in \mathcal{T}^\Delta_{n,i}} (\boldsymbol{\lambda}^\top \mathbf{h}_{n,i}(T_{i,j}, \hat{\boldsymbol{\beta}}_n))^2 \big(\mathbb{E}(G_{1,1}^4) \mathbb{P}_0(|\frac{1}{\sqrt{n}} \boldsymbol{\lambda}^\top \mathbf{h}_{n,i}(T_{i,j}, \hat{\boldsymbol{\beta}}_n) G_{1,1}| > \epsilon)\big)^{1/2} \\
&\le \sup_{t \in \mathcal{T}, i \in \{1,\dots,n\}} (\boldsymbol{\lambda}^\top \mathbf{h}_{n,i}(t, \hat{\boldsymbol{\beta}}_n))^2 (\mathbb{E}(G_{1,1}^4))^{1/2} [\mathbb{P}_0(\sup_{t \in \mathcal{T}, i \in \{1,\dots,n\}} |\boldsymbol{\lambda}^\top \mathbf{h}_{n,i}(t, \hat{\boldsymbol{\beta}}_n)||G_{1,1}| > \epsilon\sqrt{n})]^{1/2} \\
&\quad \cdot \frac{1}{n} \sum_{i=1}^n N_i(\tau),
\end{aligned}$$

where $\mathbb{E}_0(\cdot)$ and $\mathbb{P}_0(\cdot)$ denote the conditional expectation $\mathbb{E}(\cdot|\mathcal{F}_2(0))$ and the conditional probability $\mathbb{P}(\cdot|\mathcal{F}_2(0))$, respectively, given the initial filtration $\mathcal{F}_2(0)$. In the second step of the equation above we have used that $\mathbf{h}_{n,i}(t,\hat{\boldsymbol{\beta}}) \in \mathcal{F}_2(0)$. In the third step, the Cauchy-Schwarz inequality has been applied. In the same step it has additionally been used that the multiplier processes $G_i(t)$, $t \in \mathcal{T}$, $i = 1,\ldots,n$, are i.i.d. and independent of $\mathcal{F}_2(0)$. As our first goal is to verify the conditional Lindeberg condition in probability, i.e., $\mathbb{E}_0(\sigma^\epsilon[\boldsymbol{\lambda}^\top \mathbf{D}^*_{n,h}](\tau)) \xrightarrow{\mathbb{P}} 0$, $n \to \infty$, we point out that for the terms of the last step of the equation above we have $\mathbb{E}(G_{1,1}^4) < \infty$ and $\frac{1}{n}\sum_{i=1}^n N_i(\tau) = O_p(1)$. The latter holds according to the integrability of $\Lambda_i(\tau,\beta_0)$ and Assumption 1 (3), as explained at the beginning of the proof of Lemma 6 in combination with Fact 1 of the supplement of Dobler et al. [2019]. Furthermore, the limiting function $\tilde{\mathbf{h}}_i(t,\boldsymbol{\beta}_0)$ of $\mathbf{h}_{n,i}(t,\hat{\boldsymbol{\beta}}_n)$ exists and is assumed to be bounded on $\mathcal{T}$ for all $n \in \mathbb{N}$, according to Assumption 1 (1) and (2). Therefore, $\sup_{t\in\mathcal{T},i\in\{1,\ldots,n\}}(\boldsymbol{\lambda}^\top \mathbf{h}_{n,i}(t,\hat{\boldsymbol{\beta}}))^2$ is stochastically bounded:

$$\begin{aligned}
&\sup_{t\in\mathcal{T},i\in\{1,\ldots,n\}} (\boldsymbol{\lambda}^\top \mathbf{h}_{n,i}(t,\hat{\boldsymbol{\beta}}_n))^2\\
&\leq (p+b)^2\|\boldsymbol{\lambda}\|_\infty^2 \sup_{t\in\mathcal{T},i\in\{1,\ldots,n\}} \|\mathbf{h}_{n,i}(t,\hat{\boldsymbol{\beta}}_n)) - \tilde{\mathbf{h}}_i(t,\boldsymbol{\beta}_0)) + \tilde{\mathbf{h}}_i(t,\boldsymbol{\beta}_0))\|_\infty^2\\
&\leq 2(p+b)^2\|\boldsymbol{\lambda}\|_\infty^2\big(\sup_{t\in\mathcal{T},i\in\{1,\ldots,n\}} \|\mathbf{h}_{n,i}(t,\hat{\boldsymbol{\beta}}_n)) - \tilde{\mathbf{h}}_i(t,\boldsymbol{\beta}_0))\|_\infty^2 + \sup_{t\in\mathcal{T},i\in\{1,\ldots,n\}} \|\tilde{\mathbf{h}}_i(t,\boldsymbol{\beta}_0))\|_\infty^2\big)\\
&= 2(p+b)^2\|\boldsymbol{\lambda}\|_\infty^2\big(o_p(1) + \sup_{t\in\mathcal{T},i\in\{1,\ldots,n\}} \|\tilde{\mathbf{h}}_i(t,\boldsymbol{\beta}_0))\|_\infty^2\big)\\
&= O_p(1).
\end{aligned}$$

Hence, it is only left to show that $\mathbb{P}(\sup_{t\in\mathcal{T},i\in\{1,\ldots,n\}}|\boldsymbol{\lambda}^\top \mathbf{h}_{n,i}(t,\hat{\boldsymbol{\beta}}_n)||G_{1,1}| > \epsilon\sqrt{n}|\mathcal{F}_2(0)) = o_p(1)$. For this purpose, recall that $\mathbb{1}\{\sup_{t\in\mathcal{T},i\in\{1,\ldots,n\}}\|\mathbf{h}_{n,i}(t,\hat{\boldsymbol{\beta}}_n) - \tilde{\mathbf{h}}_i(t,\boldsymbol{\beta}_0)\|_\infty < \delta\}$ converges to one in probability, according to Assumption 1 (1). Thus, we can proceed with the following term:

$$\begin{aligned}
&\mathbb{P}_0(\sup_{t\in\mathcal{T},i\in\{1,\ldots,n\}} |\boldsymbol{\lambda}^\top \mathbf{h}_{n,i}(t,\hat{\boldsymbol{\beta}}_n)||G_{1,1}| > \sqrt{n}\epsilon)\mathbb{1}\{\sup_{t\in\mathcal{T},i\in\{1,\ldots,n\}} \|\mathbf{h}_{n,i}(t,\hat{\boldsymbol{\beta}}_n) - \tilde{\mathbf{h}}_i(t,\boldsymbol{\beta}_0)\|_\infty < \delta\}\\
&= \mathbb{P}_0(\sup_{t\in\mathcal{T},i\in\{1,\ldots,n\}} |\boldsymbol{\lambda}^\top \mathbf{h}_{n,i}(t,\hat{\boldsymbol{\beta}}_n) - \boldsymbol{\lambda}^\top \tilde{\mathbf{h}}_i(t,\boldsymbol{\beta}_0) + \boldsymbol{\lambda}^\top \tilde{\mathbf{h}}_i(t,\boldsymbol{\beta}_0)||G_{1,1}| > \sqrt{n}\epsilon,\\
&\qquad \sup_{t\in\mathcal{T},i\in\{1,\ldots,n\}} \|\mathbf{h}_{n,i}(t,\hat{\boldsymbol{\beta}}_n) - \tilde{\mathbf{h}}_i(t,\boldsymbol{\beta}_0)\|_\infty < \delta)\\
&\leq \mathbb{P}_0((p+b)\|\boldsymbol{\lambda}\|_\infty(\delta + \sup_{t\in\mathcal{T},i\in\{1,\ldots,n\}} \|\tilde{\mathbf{h}}_i(t,\boldsymbol{\beta}_0)\|_\infty)|G_{1,1}| > \sqrt{n}\epsilon)\\
&\quad \cdot \mathbb{1}\{\sup_{t\in\mathcal{T},i\in\{1,\ldots,n\}} \|\mathbf{h}_{n,i}(t,\hat{\boldsymbol{\beta}}_n) - \tilde{\mathbf{h}}_i(t,\boldsymbol{\beta}_0)\|_\infty < \delta\}\\
&\leq \mathbb{P}_0\big(|G_{1,1}| > \frac{\sqrt{n}\epsilon}{(p+b)\|\boldsymbol{\lambda}\|_\infty(\delta + \sup_{t\in\mathcal{T},i\in\{1,\ldots,n\}}\|\tilde{\mathbf{h}}_i(t,\boldsymbol{\beta}_0)\|_\infty)}\big)
\end{aligned}$$

$\stackrel{\mathbb{P}}{\longrightarrow} 0, \ n \to \infty.$

Here, the convergence in probability of the conditional probability in the last step holds, because $\tilde{\mathbf{h}}_i(t, \boldsymbol{\beta}_0)$ is bounded on $\mathcal{T}$ for all $i \in \mathbb{N}$, as stated in Assumption 1 (2). We can conclude that $\mathbb{P}(\sup_{t \in \mathcal{T}, i \in \{1,\dots,n\}} |\boldsymbol{\lambda}^\top \mathbf{h}_{n,i}(t, \hat{\boldsymbol{\beta}}_n)||G_{1,1}| > \epsilon\sqrt{n} | \mathcal{F}_2(0)) = o_p(1)$. Thus, the conditional Lindeberg condition in probability is fulfilled for $\boldsymbol{\lambda}^\top \mathbf{D}^*_{n,h}(t)$ with $\boldsymbol{\lambda} \in S^{p+b-1}$. As $\|\boldsymbol{\lambda}\|_\infty \leq 1$, we can get an upper bound independent of $\boldsymbol{\lambda}$, and thus we in fact know that the asserted Lindeberg condition holds for all $\boldsymbol{\lambda} \in S^{p+b-1}$. We would like to point out that the probability space can more conveniently be modelled as a product space $(\Omega, \mathcal{A}, \mathbb{P}) = (\Omega_1 \times \Omega_2, \mathcal{A}_1 \otimes \mathcal{A}_2, \mathbb{P}_1 \otimes \mathbb{P}_2) = (\Omega_1, \mathcal{A}_1, \mathbb{P}_1) \otimes (\Omega_2, \mathcal{A}_2, \mathbb{P}_2)$. In the following we make use of this notation to explicitly refer to the probability space $(\Omega_1, \mathcal{A}_1, \mathbb{P}_1)$ underlying the data sets $\{\mathbf{N}(t), \mathbf{Y}(t), \mathbf{Z}(t), t \in \mathcal{T}\}$, and the probability space $(\Omega_2, \mathcal{A}_2, \mathbb{P}_2)$ underlying the sets of multiplier processes $\{G_1(t), \dots, G_n(t), \ t \in \mathcal{T}\}$. Additionally, we denote by $\stackrel{\mathcal{L}_{\mathbb{P}_2}}{\longrightarrow}$ the convergence in law w.r.t the probability measure $\mathbb{P}_2$. Moreover, for some stochastic quantity $\mathbf{H}_n$, we denote $\mathbf{H}_n$ conditional on a particular data set as $\mathbf{H}_n | \mathcal{F}_2(0)(\omega)$, $\omega \in \Omega_1$. From the conditional Lindeberg condition in probability it follows that there exists for all subsequences n_1 of n a further subsequence n_2 such that $\mathbb{E}_{\mathbb{P}_2}(\sigma^\epsilon[\boldsymbol{\lambda}^\top \mathbf{D}^*_{n_2,h}](\tau)|\mathcal{F}_2(0))(\omega) \longrightarrow 0, \ n \to \infty$, for $\mathbb{P}_1$-almost all $\omega \in \Omega_1$ and for all $\boldsymbol{\lambda} \in S^{p+b-1}$. Here, $\mathbb{E}_{\mathbb{P}_2}(\cdot)$ indicates that the expectation is taken with respect to $\mathbb{P}_2$. Hence, the (unconditional) Lindeberg condition holds along the subsequence n_2 for $\mathbb{P}_1$-almost all data sets.

Next, we consider the predictable covariation process of $\boldsymbol{\lambda}^\top \mathbf{D}^*_{n,h}$ for some $\boldsymbol{\lambda} \in S^{p+b-1}$ and get, conditionally on $\mathcal{F}_2(0)$,

$$\langle \boldsymbol{\lambda}^\top \mathbf{D}^*_{n,h} \rangle(t) = \boldsymbol{\lambda}^\top \langle \mathbf{D}^*_{n,h} \rangle(t) \boldsymbol{\lambda} \stackrel{\mathbb{P}_1 \otimes \mathbb{P}_2}{\longrightarrow} \boldsymbol{\lambda}^\top \mathbf{V}_{\tilde{h}}(t) \boldsymbol{\lambda}, \text{ as } n \to \infty, \text{ for all } t \in \mathcal{T},$$

according to Lemma 7. Furthermore, we have

$$\begin{aligned} \boldsymbol{\lambda}^\top \big((\langle \mathbf{D}^*_{n,h} \rangle - \mathbf{V}_{\tilde{h}})(t) \big) \boldsymbol{\lambda} &= \sum_{j=1}^{p+b} \sum_{l=1}^{p+b} \lambda_j (\langle \mathbf{D}^*_{n,h} \rangle - \mathbf{V}_{\tilde{h}})_{j,l}(t) \lambda_l \\ &\leq (p+b)^2 \|\boldsymbol{\lambda}\|^2_\infty \cdot \|\langle \mathbf{D}^*_{n,h} \rangle(t) - \mathbf{V}_{\tilde{h}}(t)\|_\infty, \end{aligned}$$

where $(\langle \mathbf{D}^*_{n,h} \rangle - \mathbf{V}_{\tilde{h}})_{j,l}$ denotes the (j,l)-th entry of the corresponding matrix. As $\|\boldsymbol{\lambda}\|_\infty \leq 1$ and $\|\langle \mathbf{D}^*_{n,h} \rangle(t) - \mathbf{V}_{\tilde{h}}(t)\|_\infty = o_p(1)$, in view of Lemma 7 we thus obtain

$$\langle \boldsymbol{\lambda}^\top \mathbf{D}^*_{n,h} \rangle(t) \stackrel{\mathbb{P}_1 \otimes \mathbb{P}_2}{\longrightarrow} \boldsymbol{\lambda}^\top \mathbf{V}_{\tilde{h}}(t) \boldsymbol{\lambda}, \text{ as } n \to \infty, \text{ for all } t \in \mathcal{T}, \text{ and all } \boldsymbol{\lambda} \in S^{p+b-1}.$$

Hence, there exists for every subsequenece n_3 of n_2 a further subsequence n_4 such that $\langle \boldsymbol{\lambda}^\top \mathbf{D}^*_{n_4,h} \rangle | \mathcal{F}_2(0)(\omega) \stackrel{\mathbb{P}_2}{\longrightarrow} \boldsymbol{\lambda}^\top \mathbf{V}_{\tilde{h}}(t) \boldsymbol{\lambda}$, as $n \to \infty$, for $\mathbb{P}_1$-almost all $\omega \in \Omega_1$, all $t \in \mathcal{T}$, and all $\boldsymbol{\lambda} \in S^{p+b-1}$. Clearly, it also holds that

$\mathbb{E}_{\mathbb{P}_2}(\sigma^\epsilon[\boldsymbol{\lambda}^\top \mathbf{D}^*_{n_4,h}](\tau)|\mathcal{F}_2(0))(\omega) \to 0$, $n \to \infty$, for $\mathbb{P}_1$-almost all $\omega \in \Omega_1$ and all $\boldsymbol{\lambda} \in S^{p+b-1}$. Thus, with Theorem 1 it follows that

$$\boldsymbol{\lambda}^\top \mathbf{D}^*_{n_4,h}|\mathcal{F}_2(0)(\omega) \xrightarrow{\mathcal{L}_{\mathbb{P}_2}} \boldsymbol{\lambda}^\top \mathbf{D}_{\tilde{h}}, \text{ in } D(\mathcal{T}), \text{ as } n \to \infty,$$

for $\mathbb{P}_1$-almost all $\omega \in \Omega_1$ and all $\boldsymbol{\lambda} \in S^{p+b-1}$. As the weak convergence of $\boldsymbol{\lambda}^\top \mathbf{D}^*_{n_4,h}|\mathcal{F}_2(0)(\omega)$ holds for all $\boldsymbol{\lambda} \in S^{p+b-1}$, the Cramér-Wold device yields $\mathbf{D}^*_{n_4,h}|\mathcal{F}_2(0)(\omega) \xrightarrow{\mathcal{L}_{\mathbb{P}_2}} \mathbf{D}_{\tilde{h}}$, in $D(\mathcal{T})^{p+b}$, as $n \to \infty$, for $\mathbb{P}_1$-almost all $\omega \in \Omega_1$. Finally, we get, conditionally on $\mathcal{F}_2(0)$,

$$\mathbf{D}^*_{n,h} \xrightarrow{\mathcal{L}_{\mathbb{P}_2}} \mathbf{D}_{\tilde{h}}, \text{ in } D(\mathcal{T})^{p+b}, \text{ as } n \to \infty,$$

in $\mathbb{P}_1$-probability. This completes the proof of Lemma 8. □

In the proof of Lemma 8 above, one can see that Assumption 1 implies the Lindeberg condition for the stochastic process $\mathbf{D}^*_{n,h}$. Thus, Corollary 1 below is a direct consequence of Theorem 1 and Lemma 7. However, for didactic reasons, we provide an alternative proof of Corollary 1 that is based on Lenglart's inequality.

Corollary 1 *If Assumption 1 holds, then, conditionally on $\mathcal{F}_2(0)$, $[\boldsymbol{D}^*_{n,h}](t) \xrightarrow{\mathbb{P}} \boldsymbol{V}_{\tilde{h}}(t)$, as $n \to \infty$, for all $t \in \mathcal{T}$, with $\boldsymbol{V}_{\tilde{h}}$ as defined in Lemma 5.*

Proof of Corollary 1.
We relate the optional covariation process $[\mathbf{D}^*_{n,h}](t)$ and the predictable covariation process $\langle \mathbf{D}^*_{n,h} \rangle(t)$ of $\mathbf{D}^*_{n,h}(t)$ to each other by noting the obvious

$$[\mathbf{D}^*_{n,h}](t) = [\mathbf{D}^*_{n,h}](t) - \langle \mathbf{D}^*_{n,h} \rangle(t) + \langle \mathbf{D}^*_{n,h} \rangle(t).$$

Consequently, if the predictable covariation process $\langle \mathbf{D}^*_{n,h} \rangle(t)$ converges in probability to $\mathbf{V}_{\tilde{h}}(t)$, as $n \to \infty$, and it holds that $[\mathbf{D}^*_{n,h}](t) - \langle \mathbf{D}^*_{n,h} \rangle(t) = o_p(1)$, then also the optional covariation $[\mathbf{D}^*_{n,h}](t)$ converges in probability to $\mathbf{V}_{\tilde{h}}(t)$, as $n \to \infty$, and vice versa. Hence, for this proof we assume that Lemma 7 holds and show that the difference between the optional covariation process and the predictable covariation process of $\mathbf{D}^*_{n,h}(t)$ vanishes asymptotically.

Let us consider the vectorized version $\mathbf{Q}_n$ of the difference between the optional covariation process and the predictable covariation process of $\mathbf{D}^*_{n,h}(t)$, $t \in \mathcal{T}$,

$$\begin{aligned}\mathbf{Q}_n(t) &= \text{vec}([\mathbf{D}^*_{n,h}](t) - \langle \mathbf{D}^*_{n,h} \rangle(t)) \\ &= \frac{1}{n} \sum_{i=1}^{n} \int_0^t \text{vec}(\mathbf{h}_{n,i}(u, \hat{\boldsymbol{\beta}}_n)^{\otimes 2})(G_i^2(u) - 1) dN_i(u).\end{aligned}$$

The $\text{vec}(\mathbf{h}_{n,i}(t, \hat{\boldsymbol{\beta}}_n)^{\otimes 2})$ in the integrands are known and locally bounded and predictable. Hence, according to Theorem II.3.1 of Andersen et al. [1993], $\mathbf{Q}_n$ is a vector of local square integrable martingales if $\int_0^\cdot (G_i^2(u) - 1) dN_i(u)$ is a finite variation local square integrable martingale for all $i = 1, \ldots, n$. This is what we show in the following three steps.

1. The finite variation holds, because

$$\int_0^\tau |(G_i^2(u)-1)dN_i(u)| \le (\sup_{t\in\mathcal{T}} G_i^2(t)+1)N_i(\tau),$$

and the term on the right-hand side is almost surely finite as $N_i(\tau)<\infty$, and the supremum is a maximum of almost surely finitely many random variables.

2. It is square integrable, since

$$\begin{aligned}\sup_{t\in\mathcal{T}} \mathbb{E}_0\Big(\Big[\int_0^t (G_i^2(u)-1)dN_i(u)\Big]^2\Big) &= \sup_{t\in\mathcal{T}} \mathbb{E}_0\Big(\Big[\sum_{j:T_{i,j}\le t} (G_{i,j}^2-1)\Big]^2\Big)\\ &\le \sup_{t\in\mathcal{T}} \mathbb{E}_0\Big(\big|\{j:T_{i,j}\le t\}\big| \sum_{j:T_{i,j}\le t}(G_{i,j}^2-1)^2\Big)\\ &\le N_i(\tau)\sum_{j=1}^{n_i}\mathbb{E}(G_{i,j}^4-2G_{i,j}^2+1)\\ &\le N_i(\tau)^2\mathbb{E}(G_{1,1}^4)<\infty,\end{aligned}$$

where $\mathbb{E}_0(\cdot)$ denotes the conditional expectation $\mathbb{E}(\cdot|\mathcal{F}_2(0))$ and $\big|\{j:T_{i,j}\le t\}\big|$ the cardinality of the corresponding set. Moreover, in the third step we have applied that the counting processes $N_i(t)$, $t\in\mathcal{T}$, are $\mathcal{F}_2(0)$-measurable, whereas the values of $G_{i,j}$ and the filtration $\mathcal{F}_2(0)$ are independent for all $j=1,\dots,n_i$ and $i=1,\dots,n$. Additionally, in the fourth step we used that $G_{i,1},\dots,G_{i,n_i}$ are identically distributed with zero mean, unit variance and finite fourth moment for all $i=1,\dots,n$.

3. The martingale property is valid, as

$$\begin{aligned}&\mathbb{E}\big(\int_0^t (G_i^2(u)-1)dN_i(u)|\mathcal{F}_2(s)\big)\\ &=\mathbb{E}\big(\int_0^s (G_i^2(u)-1)dN_i(u)+\int_s^t (G_i^2(u)-1)dN_i(u)|\mathcal{F}_2(s)\big)\\ &=\int_0^s (G_i^2(u)-1)dN_i(u)+\int_s^t \big(\mathbb{E}(G_i^2(u))-1\big)dN_i(u)\\ &=\int_0^s (G_i^2(u)-1)dN_i(u),\end{aligned}$$

where in the second step we have used that the counting process $N_i(t)$ is $\mathcal{F}_2(0)\subset\mathcal{F}_2(t)$-measurable for $t\in\mathcal{T}$, $i=1,\dots,n$. Furthermore, for a jump at $u\le s$, the multiplier process $G_i(u)$ is $\mathcal{F}_2(s)$-measurable, and, if u is greater than or equal to the earliest jump time point, say $T_i(s^+)$, of process N_i in $(s,\tau]$, the values of $G_i(u)$ and the filtration $\mathcal{F}_2(s)$ are independent, $i=1,\dots,n$. In the third step we used that the multiplier processes $G_i(t)$, $t\in\mathcal{T}$, have zero mean and unit variance, $i=1,\dots,n$.

In conclusion, $\mathbf{Q}_n$ is a vector of local square integrable martingales.

Next, we wish to show that $\mathbf{Q}_n(t)$ converges to zero in probability, as $n \to \infty$. For this we apply Lenglart's inequality and consider the predictable covariation process $\langle \mathbf{Q}_n \rangle(\tau)$ of the martingale $\mathbf{Q}_n$ at τ

$$\begin{aligned}\langle \mathbf{Q}_n \rangle(\tau) &= \Big\langle \frac{1}{n} \sum_{i=1}^{n} \int_0^{\cdot} \operatorname{vec}(\mathbf{h}_{n,i}(u, \hat{\boldsymbol{\beta}}_n)^{\otimes 2})(G_i^2(u) - 1) dN_i(u) \Big\rangle(\tau) \\ &= \frac{1}{n^2} \sum_{i=1}^{n} \int_0^{\tau} \operatorname{vec}(\mathbf{h}_{n,i}(u, \hat{\boldsymbol{\beta}}_n)^{\otimes 2})^{\otimes 2} d\Big\langle \int_0^{\cdot} (G_i^2(v) - 1) dN_i(v) \Big\rangle(u) \\ &= \frac{1}{n^2} \sum_{i=1}^{n} \int_0^{\tau} \operatorname{vec}(\mathbf{h}_{n,i}(u, \hat{\boldsymbol{\beta}}_n)^{\otimes 2})^{\otimes 2} (\mathbb{E}(G_i^4(u)) - 1) dN_i(u),\end{aligned}$$

where in the second step we have used that

$$\begin{aligned}& d\Big\langle \int_0^{\cdot} (G_i^2(u) - 1) dN_i(u), \int_0^{\cdot} (G_l^2(u) - 1) dN_l(u) \Big\rangle(t) \\ &= \operatorname{Cov}\big((G_i^2(t) - 1) dN_i(t), (G_l^2(t) - 1) dN_l(t) | \mathcal{F}_{t\text{-}}\big) \\ &= \operatorname{Cov}\big(G_i^2(t), G_l^2(t)\big) dN_i(t) dN_l(t) \\ &= 0,\end{aligned}$$

because $G_1(t), \ldots, G_n(t)$, $t \in \mathcal{T}$, are pairwise independent and no two counting processes jump simultaneously. The third step holds due to

$$\begin{aligned}d\Big\langle \int_0^{\cdot} (G_i^2(u) - 1) dN_i(u) \Big\rangle(t) &= \mathbb{E}\big([(G_i^2(t) - 1) dN_i(t)]^2 | \mathcal{F}_{t\text{-}}\big) \\ &= \big(\mathbb{E}(G_i^4(t)) - 2\mathbb{E}(G_i(t)^2) + 1\big) dN_i(t) \\ &= \big(\mathbb{E}(G_i^4(t)) - 1\big) dN_i(t).\end{aligned}$$

We continue by stating that

$$\begin{aligned}\langle \mathbf{Q}_n \rangle(\tau) = \frac{1}{n^2} \sum_{i=1}^{n} \int_0^{\tau} \operatorname{vec}(\tilde{\mathbf{h}}_i(u, \boldsymbol{\beta}_0)^{\otimes 2})^{\otimes 2} (\mathbb{E}(G_i^4(u)) - 1) dN_i(u) \qquad (61) \\ + \frac{1}{n^2} \sum_{i=1}^{n} \int_0^{\tau} [\operatorname{vec}(\mathbf{h}_{n,i}(u, \hat{\boldsymbol{\beta}}_n)^{\otimes 2})^{\otimes 2} - \operatorname{vec}(\tilde{\mathbf{h}}_i(u, \boldsymbol{\beta}_0)^{\otimes 2})^{\otimes 2}] (\mathbb{E}(G_i^4(u)) - 1) dN_i(u).\end{aligned}$$

For the first term on the right-hand side of (61) we have

$$\begin{aligned}& \frac{1}{n^2} \sum_{i=1}^{n} \int_0^{\tau} \operatorname{vec}(\tilde{\mathbf{h}}_i(u, \boldsymbol{\beta}_0)^{\otimes 2})^{\otimes 2} (\mathbb{E}(G_i^4(u)) - 1) dN_i(u) \\ &\le \frac{1}{n} \sup_{i \in \{1, \ldots, n\}, t \in \mathcal{T}} \| \tilde{\mathbf{h}}_i(t, \boldsymbol{\beta}_0) \|_\infty^4 (\mathbb{E}(G_{1,1}^4) - 1) \frac{1}{n} \sum_{i=1}^{n} N_i(\tau) = o_p(1), \text{ as } n \to \infty, \qquad (62)\end{aligned}$$

since $\mathbb{E}(G_{1,1}^4) < \infty$ according to Assumption 1 (2), and $\frac{1}{n}\sum_{i=1}^n N_i(\tau) = O_p(1)$, as was derived at the beginning of the proof of Lemma 6. Additionally, for the second term on the right-hand side of (61) we find

$$\begin{aligned}
&\|\frac{1}{n^2}\sum_{i=1}^n \int_0^\tau [\mathrm{vec}(\mathbf{h}_{n,i}(u,\hat{\boldsymbol{\beta}}_n)^{\otimes 2})^{\otimes 2} - \mathrm{vec}(\tilde{\mathbf{h}}_i(u,\boldsymbol{\beta}_0)^{\otimes 2})^{\otimes 2}](\mathbb{E}(G_i^4(u)) - 1)dN_i(u)\|_\infty \\
&\leq \frac{1}{n}\sup_{i\in\{1,\dots,n\},t\in\mathcal{T}} \|\mathrm{vec}(\mathbf{h}_{n,i}(t,\hat{\boldsymbol{\beta}}_n)^{\otimes 2})^{\otimes 2} - \mathrm{vec}(\tilde{\mathbf{h}}_i(t,\boldsymbol{\beta}_0)^{\otimes 2})^{\otimes 2}\|_\infty \\
&\quad \cdot (\mathbb{E}(G_{1,1}^4) - 1)\frac{1}{n}\sum_{i=1}^n N_i(\tau) \\
&\leq \sup_{i\in\{1,\dots,n\},t\in\mathcal{T}} \Big(\|\mathbf{h}_{n,i}(t,\hat{\boldsymbol{\beta}}_n)\|_\infty^2 \Big[\|\mathbf{h}_{n,i}(t,\hat{\boldsymbol{\beta}}_n) - \tilde{\mathbf{h}}_i(t,\boldsymbol{\beta}_0)\|_\infty \|\mathbf{h}_{n,i}(t,\hat{\boldsymbol{\beta}}_n)\|_\infty \\
&\quad + \|\tilde{\mathbf{h}}_i(t,\boldsymbol{\beta}_0)\|_\infty \|\mathbf{h}_{n,i}(t,\hat{\boldsymbol{\beta}}_n) - \tilde{\mathbf{h}}_i(t,\boldsymbol{\beta}_0)\|_\infty \Big] \\
&\quad + \|\tilde{\mathbf{h}}_i(t,\boldsymbol{\beta}_0)\|_\infty^2 \Big[\|\mathbf{h}_{n,i}(t,\hat{\boldsymbol{\beta}}_n) - \tilde{\mathbf{h}}_i(t,\boldsymbol{\beta}_0)\|_\infty \|\mathbf{h}_{n,i}(t,\hat{\boldsymbol{\beta}}_n)\|_\infty \\
&\quad + \|\tilde{\mathbf{h}}_i(t,\boldsymbol{\beta}_0)\|_\infty \|\mathbf{h}_{n,i}(t,\hat{\boldsymbol{\beta}}_n) - \tilde{\mathbf{h}}_i(t,\boldsymbol{\beta}_0)\|_\infty \Big] \Big) \\
&\quad \cdot \frac{1}{n}(\mathbb{E}(G_{1,1}^4) - 1)\frac{1}{n}\sum_{i=1}^n N_i(\tau) \\
&= o_p(1), \text{ as } n \to \infty,
\end{aligned} \tag{63}$$

where we used $\mathbb{E}(G_{1,1}^4) < \infty$, $\frac{1}{n}\sum_{i=1}^n N_i(\tau) = O_p(1)$, $\|\mathbf{h}_{n,i}(t,\hat{\boldsymbol{\beta}}_n)\|_\infty < \infty$, Assumption 1 (1), (2), and (60) in combination with the triangle inequality. In particular, the terms in brackets vanish asymptotically, as $n \to \infty$. Combining (61), (62) and (63), we get $\langle \mathbf{Q}_n \rangle(\tau) = o_p(1)$, as $n \to \infty$, and with Lenglart's inequality it follows that

$$\mathbf{Q}_n(t) = \mathrm{vec}\big([\mathbf{D}_{n,h}^*](t) - \langle \mathbf{D}_{n,h}^* \rangle(t)\big) \xrightarrow{\mathbb{P}} 0, \text{ as } n \to \infty, \text{ for all } t \in \mathcal{T}.$$

In combination with Lemma 7, we have $[\mathbf{D}_{n,h}^*](t) \xrightarrow{\mathbb{P}} \mathbf{V}_{\tilde{h}}(t)$, as $n \to \infty$, for all $t \in \mathcal{T}$. This completes the proof of Corollary 1. □

After having assessed the joint convergence in distribution of $\mathbf{D}_{n,h}^* = (\mathbf{D}_{n,k}^*, \mathbf{D}_{n,g}^*)$ by means of Lemma 8, we focus again on the representation of $\sqrt{n}(\mathbf{X}_n^* - \mathbf{X}_n) = \mathbf{D}_{n,k}^* + \mathbf{B}_n^*\mathbf{C}_n^*\mathbf{D}_{n,g}^*(\tau) + o_p(1)$ given in Lemma 2 of the main manuscript. We first address the convergence of the components $\boldsymbol{B}_n^*$ and $\mathbf{C}_n^*$ before we eventually consider the representation as a whole.

Lemma 9 *If Assumption 1 (3) and Assumption 2 hold, then, conditionally on* $\mathcal{F}_2(0)$, $\sup_{t\in\mathcal{T}}\|\boldsymbol{B}_n^*(t) - \boldsymbol{B}(t)\| \xrightarrow{\mathbb{P}} 0$, *as* $n \to \infty$ *with* $\boldsymbol{B}$ *as in Lemma 6.*

Proof of Lemma 9.
Recall that $\mathbf{B}_n^*(t) = \frac{1}{n}\sum_{i=1}^n \int_0^t \mathrm{D}\mathbf{k}_{n,i}(u,\hat{\boldsymbol{\beta}}_n)\big(G_i(u)+1\big)dN_i(u)$. Then, we have

$$\begin{aligned}
&\sup_{t\in\mathcal{T}} \|\mathbf{B}_n^*(t) - \mathbf{B}(t)\| \\
&\le \sup_{t\in\mathcal{T}} \Big\| \frac{1}{n}\sum_{i=1}^n \int_0^t [\mathrm{D}\mathbf{k}_{n,i}(u,\boldsymbol{\beta}_0) - \tilde{\mathbf{K}}_i(u,\boldsymbol{\beta}_0)](G_i(u)+1)dN_i(u)\Big\| \\
&\quad + \sup_{t\in\mathcal{T}} \Big\| \frac{1}{n}\sum_{i=1}^n \int_0^t \tilde{\mathbf{K}}_i(u,\boldsymbol{\beta}_0)(G_i(u)+1)dN_i(u) - \mathbf{B}(t)\Big\| \\
&\le \sup_{t\in\mathcal{T}} \Big\| \frac{1}{n}\sum_{i=1}^n \int_0^t [\mathrm{D}\mathbf{k}_{n,i}(u,\boldsymbol{\beta}_0) - \tilde{\mathbf{K}}_i(u,\boldsymbol{\beta}_0)](G_i(u)+1)dN_i(u)\Big\| \\
&\quad + \sup_{t\in\mathcal{T}} \Big\| \frac{1}{n}\sum_{i=1}^n \int_0^t \tilde{\mathbf{K}}_i(u,\boldsymbol{\beta}_0)G_i(u)dN_i(u)\Big\| \\
&\quad + \sup_{t\in\mathcal{T}} \Big\| \frac{1}{n}\sum_{i=1}^n \int_0^t \tilde{\mathbf{K}}_i(u,\boldsymbol{\beta}_0)dN_i(u) - \mathbf{B}(t)\Big\|.
\end{aligned} \tag{64}$$

We consider the second term on the right-hand side of the second step of (64) first. According to Lemma 3 with $h_{n,i}(t,\hat{\beta}_n) \equiv 1$, $\int_0^t G_i(u)\,dN_i(u)$ is a square integrable martingale w.r.t. $\mathcal{F}_2$. Moreover, it holds that $\int_0^\tau |G_i(u)\,dN_i(u)| \le \max_{j=1,\dots,n_i}|G_{i,j}|N_i(\tau) < \infty$ almost surely, as the maximum is taken over finitely many almost surely finite random variables. Thus, the martingale is also of finite variation. Due to Assumption 2 (2) and with Theorem II.3.1. of Andersen et al. [1993], it follows that $\frac{1}{n}\sum_{i=1}^n \int_0^t \tilde{\mathbf{K}}_i(u,\boldsymbol{\beta}_0)G_i(u)dN_i(u)$ is a local square integrable martingale w.r.t. $\mathcal{F}_2$. Furthermore, its predictable covariation process at τ is given by

$$\begin{aligned}
&\Big\langle \frac{1}{n}\sum_{i=1}^n \int_0^\cdot \tilde{\mathbf{K}}_i(u,\boldsymbol{\beta}_0)G_i(u)dN_i(u)\Big\rangle(\tau) \\
&= \frac{1}{n^2}\sum_{i=1}^n\sum_{j=1}^n \Big\langle \int_0^\cdot \tilde{\mathbf{K}}_i(u,\boldsymbol{\beta}_0)G_i(u)dN_i(u), \int_0^\cdot \tilde{\mathbf{K}}_j(u,\boldsymbol{\beta}_0)G_j(u)dN_j(u)\Big\rangle(\tau) \\
&= \frac{1}{n^2}\sum_{i=1}^n\sum_{j=1}^n \int_0^\tau \tilde{\mathbf{K}}_i(u,\boldsymbol{\beta}_0)\, d\Big\langle \int_0^\cdot G_i(s)dN_i(s), \int_0^\cdot G_j(s)dN_j(s)\Big\rangle(u)\, \tilde{\mathbf{K}}_j(u,\boldsymbol{\beta}_0)^\top \\
&= \frac{1}{n^2}\sum_{i=1}^n \int_0^\tau \tilde{\mathbf{K}}_i(u,\boldsymbol{\beta}_0)^{\otimes 2}\, dN_i(u),
\end{aligned} \tag{65}$$

because $\langle \int_0^\cdot G_i(s)dN_i(s), \int_0^\cdot G_j(s)dN_j(s)\rangle(u) = N_i(u)$, for $i = j$, and zero otherwise, according to Lemma 3. Additionally, in the second step of (65) the aforementioned Theorem II.3.1. has been used. For the remaining part of this proof we use unconditional convergence in probability instead of conditionally on

$\mathcal{F}_2(0)$, because due to Fact 1 of the supplement of Dobler et al. [2019] these two types of convergence are equivalent. We wish to show that the last term on the right-hand side of (65) converges to zero in probability, as $n \to \infty$. For this, we bound that term from above by $\frac{1}{n} \sup_{i\in\{1,...,n\},t\in\mathcal{T}} \|\tilde{\mathbf{K}}_i(t, \boldsymbol{\beta}_0)\|_\infty^2 \frac{1}{n} \sum_{i=1}^n N_i(\tau)$. Recall that $\sup_{i\in\{1,...,n\},t\in\mathcal{T}} \|\tilde{\mathbf{K}}_i(t, \boldsymbol{\beta}_0)\|_\infty^2 < \infty$, by Assumption 2 (2), and $\frac{1}{n} \sum_{i=1}^n N_i(\tau) = O_p(1)$, by the integrability of $\Lambda_i(\tau, \beta_0)$ and Assumption 1 (3), as stated at the beginning of the proof of Lemma 6. Hence, the predictable covariation process of $\frac{1}{n} \sum_{i=1}^n \int_0^t \tilde{\mathbf{K}}_i(u, \boldsymbol{\beta}_0) G_i(u) dN_i(u)$ at τ converges to zero in probability, as $n \to \infty$. With Lenglart's inequality it follows that the corresponding martingale converges to zero in probability, as $n \to \infty$, for all $t \in \mathcal{T}$. In other words, the second term on the right-hand side of the second step of (64) vanishes asymptotically.

Next, we consider the first term on the right-hand side of the second step of (64). For this term we get

$$
\begin{aligned}
&\sup_{t\in\mathcal{T}} \|\frac{1}{n} \sum_{i=1}^n \int_0^t [\mathrm{D}\mathbf{k}_{n,i}(u, \boldsymbol{\beta}_0) - \tilde{\mathbf{K}}_i(u, \boldsymbol{\beta}_0)](G_i(u) + 1) dN_i(u)\| \\
&\leq \sup_{i\in\{1,...,n\},t\in\mathcal{T}} \|\mathrm{D}\mathbf{k}_{n,i}(t, \hat{\boldsymbol{\beta}}) - \tilde{\mathbf{K}}_i(t, \boldsymbol{\beta}_0)\| \frac{1}{n} \sum_{i=1}^n \int_0^\tau |G_i(u) + 1| \, dN_i(u).
\end{aligned}
$$

According to Assumption 2 (1), the first term on the right-hand side of the inequality above converges to zero in probability, as $n \to \infty$. We now address the corresponding second term, which can be rewritten as $\frac{1}{n} \sum_{i=1}^n \sum_{j=1}^{n_i} |G_{i,j} + 1|$. Furthermore, we have

$$
\begin{aligned}
\mathbb{E}\Big(\sum_{j=1}^{n_i} |G_{i,j} + 1|\Big) &= \mathbb{E}\Big(\mathbb{E}\Big(\sum_{j=1}^{n_i} |G_{i,j} + 1| | \mathcal{F}_2(0)\Big)\Big) \\
&= \mathbb{E}\Big(\sum_{j=1}^{n_i} \mathbb{E}(|G_{i,j} + 1|)\Big) \\
&\leq 2\mathbb{E}(N_i(\tau)) < \infty,
\end{aligned} \tag{66}
$$

where in the second step we have used that $N_i(t)$ with $N_i(\tau) = n_i$ is $\mathcal{F}_2(0)$-measurable and $G_i(t)$, $t \in \mathcal{T}$, is independent of $\mathcal{F}_2(0)$. Additionally, in the last step of (66) we employed that $\mathrm{Var}(|G_{i,j}|) = \mathbb{E}(G_{i,j}^2) - \mathbb{E}(|G_{i,j}|)^2 \geq 0$ and $\mathbb{E}(G_{i,j}^2) = 1$ implies $\mathbb{E}(|G_{i,j}|) \leq 1$. As the pairs $(G_i(t), N_i(t))$ are pairwise independent and identically distributed, it follows with (66) and the law of large numbers that $\frac{1}{n} \sum_{i=1}^n \sum_{j=1}^{n_i} |G_{i,j} + 1| \xrightarrow{\mathbb{P}} \mathbb{E}(\sum_{j=1}^{n_1} |G_{1,j} + 1|)$, as $n \to \infty$. Finally, we conclude that $\frac{1}{n} \sum_{i=1}^n \int_0^\tau |G_i(u) + 1| \, dN_i(u) = O_p(1)$, which is why also the first term on the right-hand side of the second step of (64) converges to zero in probability, as $n \to \infty$. It is only left to consider the third term on the right-hand side of the second step of (64). In fact, we have already shown in the proof of Lemma 6 that this term converges to zero in probability, as

$n \to \infty$. Thus, we have proven that all three terms of (64) converge to zero in probability, as $n \to \infty$, which completes the proof of Lemma 9. $\square$

Proof of Theorem 3.
We aim to derive the weak limit of the term $\mathbf{D}^*_{n,k} + \mathbf{B}^*_n \mathbf{C}^*_n \mathbf{D}^*_{n,g}(\tau)$, as $n \to \infty$, where $\mathbf{D}^*_{n,k}$ and $\mathbf{D}^*_{n,g}$ are vector-valued stochastic processes, $\mathbf{B}^*_n$ is a matrix-valued stochastic process and $\mathbf{C}^*_n$ is a random matrix. Recall the notation introduced in the proof of Lemma 8 regarding the product probability space $(\Omega_1, \mathcal{A}_1, \mathbb{P}_1) \otimes (\Omega_2, \mathcal{A}_2, \mathbb{P}_2)$, the convergence in law w.r.t $\mathbb{P}_2$, $\xrightarrow{\mathcal{L}_{\mathbb{P}_2}}$, and $\cdot|\mathcal{F}_2(0)(\omega)$. According to Lemma 8, we have, conditionally on $\mathcal{F}_2(0)$, $({\mathbf{D}^*_{n,k}}^\top, {\mathbf{D}^*_{n,g}}^\top)^\top = \mathbf{D}^*_{n,h} \xrightarrow{\mathcal{L}_{\mathbb{P}_2}} \mathbf{D}_{\tilde{h}}$, in $(D(\mathcal{T}))^{p+b}$, as $n \to \infty$, in $\mathbb{P}_1$-probability, where $\mathbf{D}_{\tilde{h}}$ is given in Theorem 2. Thus, for every subsequence n_1 of n there exists a further subsequence n_2 such that

$$\mathbf{D}^*_{n_2,h}|\mathcal{F}_2(0)(\omega) \xrightarrow{\mathcal{L}_{\mathbb{P}_2}} \mathbf{D}_{\tilde{h}}, \text{ in } (D(\mathcal{T}))^{p+b}, \text{ as } n \to \infty, \tag{67}$$

for $\mathbb{P}_1$-almost all $\omega \in \Omega_1$. Moreover, with Lemma 9 it follows that, conditionally on $\mathcal{F}_2(0)$, $\mathbf{B}^*_{n_2}(t) \xrightarrow{\mathbb{P}_1 \otimes \mathbb{P}_2} \mathbf{B}(t)$ uniformly in $t \in \mathcal{T}$, as $n \to \infty$. Hence, for every subsequence n_3 of n_2 there exists a further subsequence n_4 such that $\mathbf{B}^*_{n_4}(t)|\mathcal{F}_2(0)(\omega) \xrightarrow{\mathbb{P}_2} \mathbf{B}(t)$, as $n \to \infty$, uniformly in $t \in \mathcal{T}$, for $\mathbb{P}_1$-almost all $\omega \in \Omega_1$. Consequently, we have

$$\mathbf{B}^*_{n_4}|\mathcal{F}_2(0)(\omega) \xrightarrow{\mathcal{L}_{\mathbb{P}_2}} \mathbf{B}, \text{ in } (\mathcal{D}(\mathcal{T}))^{pq}, \text{ as } n \to \infty, \tag{68}$$

for $\mathbb{P}_1$-almost all $\omega \in \Omega_1$. Clearly, (67) also holds along the subsequence n_4. Furthermore, we assume that, conditionally on $\mathcal{F}_2(0)$, $\mathbf{C}^*_n$ converges in $\mathbb{P}_1 \otimes \mathbb{P}_2$-probability to $\mathbf{C}$, i.e., the limits of $\mathbf{C}^*_n$ and $\mathbf{C}_n$, given in Section 2, are identical. Thus, for every subsequence n_5 of n_4 there exists a further subsequence n_6 such that $\mathbf{C}^*_{n_6}|\mathcal{F}_2(0)(\omega) \xrightarrow{\mathbb{P}_2} \mathbf{C}$, as $n \to \infty$, for $\mathbb{P}_1$-almost all $\omega \in \Omega_1$. Again, it follows that

$$\mathbf{C}^*_{n_6}|\mathcal{F}_2(0)(\omega) \xrightarrow{\mathcal{L}_{\mathbb{P}_2}} \mathbf{C}, \text{ as } n \to \infty,$$

for $\mathbb{P}_1$-almost all $\omega \in \Omega_1$. Obviously, (67) and (68) also hold along the subsequence n_6. Then,

$$(\mathbf{D}^*_{n_6,h}, \mathbf{B}^*_{n_6}, \mathbf{C}^*_{n_6})|\mathcal{F}_2(0)(\omega) \xrightarrow{\mathcal{L}_{\mathbb{P}_2}} (\mathbf{D}_{\tilde{h}}, \mathbf{B}, \mathbf{C}) \text{ in } \mathcal{D}[0,\tau]^{p+b+pq} \times \mathbb{R}^{pq}, \text{ as } n \to \infty,$$

for $\mathbb{P}_1$-almost all $\omega \in \Omega_1$ follows analogously to the proof of Theorem 2. Eventually, the continuous mapping theorem with, successively, the functions f_1, f_2, and f_3 given in the proof of Theorem 2 are applied to $(\mathbf{D}^*_{n_6,h}, \mathbf{B}^*_{n_6}, \mathbf{C}^*_{n_6})|\mathcal{F}_2(0)(\omega)$. In particular, we get $\mathbf{D}^*_{n_6,k} + \mathbf{B}^*_{n_6} \mathbf{C}^*_{n_6} \mathbf{D}^*_{n_6,g}(\tau)|\mathcal{F}_2(0)(\omega) \xrightarrow{\mathcal{L}_{\mathbb{P}_2}} \mathbf{D}_{\tilde{k}} + \mathbf{B}\mathbf{C}\mathbf{D}_{\tilde{g}}(\tau)$ for $\mathbb{P}_1$-almost all $\omega \in \Omega_1$. Finally, by invoking the help of the subsequence principle again, we can conclude that, conditionally on $\mathcal{F}_2(0)$,

$$\mathbf{D}^*_{n,k} + \mathbf{B}^*_n \mathbf{C}^*_n \mathbf{D}^*_{n,g}(\tau) \xrightarrow{\mathcal{L}_{\mathbb{P}_2}} \mathbf{D}_{\tilde{k}} + \mathbf{B}\mathbf{C}\mathbf{D}_{\tilde{g}}(\tau), \text{ in } \mathcal{D}(\mathcal{T}))^p, \text{ as } n \to \infty,$$

in $\mathbb{P}_1$-probability. Moreover, we can summarize the results of Theorem 2 and Theorem 3 with the following statement:

$$d[\mathcal{L}_{\mathbb{P}_2}(\sqrt{n}(\mathbf{X}_n^* - \mathbf{X}_n)|\mathcal{F}_2(0)), \mathcal{L}_{\mathbb{P}_1}(\sqrt{n}(\mathbf{X}_n - \mathbf{X}))] \xrightarrow{\mathbb{P}_1} 0, \text{ as } n \to \infty.$$

□

Acknowledgements The authors would like to thank the Associate Editor and two reviewers whose comments have significantly improved our paper. Dennis Dobler would like to thank his affiliations, Department of Statistics (TU Dortmund University) and Research Center Trustworthy Data Science and Security (University Alliance Ruhr) where a smaller part of the work has been done.

Conflict of interest

The authors declare that they have no conflict of interest.

References

Per Kragh Andersen, Ørnulf Borgan, Richard D. Gill, and Niels Keiding. Statistical Models Based on Counting Processes. Springer, New York, 1993.

Aad W. van der Vaart and Jon A. Wellner. Weak Convergence and Empirical Processes. With Applications to Statistics. Springer, New York, 1996.

Dennis Dobler, Markus Pauly, and Thomas H. Scheike. Confidence bands for multiplicative hazards models: Flexible resampling approaches. Biometrics, 75(3):906–916, 2019.
